# Photo-induced copper-catalyzed sequential 1,n-HAT enabling the formation of cyclobutanols

**DOI:** 10.1038/s41467-021-26670-5

**Published:** 2021-11-04

**Authors:** Zhusong Cao, Jianye Li, Guozhu Zhang

**Affiliations:** 1 College of Chemistry, Central China Normal University (CCNU), 152 Luoyu Road, Wuhan, Hubei 430079 P. R. China; 2 State Key Laboratory of Organometallic Chemistry, Shanghai Institute of Organic Chemistry, Center for Excellence in Molecular Synthesis, University of Chinese Academy of Sciences, Chinese Academy of Sciences, 345 Lingling Road, Shanghai, 200032 P. R. China

**Keywords:** Synthetic chemistry methodology, Photocatalysis

## Abstract

Cyclobutanols are privileged cyclic skeletons in natural products and synthetic building blocks. C(sp3)−H functionalization is a prolonged challenge in organic synthesis. The synthesis of cyclobutanols through double C(sp3)-H bond functionalization remains elusive. Here we report the efficient synthesis of cyclobutanols through intermolecular radical [3 + 1] cascade cyclization, involving the functionalization of two C − H bonds through sequential hydrogen atom transfer. The copper complex reduces the iodomethylsilyl alcohols efficiently under blue-light irradiation to initiate the tandem transformation. The mild reaction tolerates a broad range of functional groups and allows for the facile generation of elaborate polycyclic structures.

## Abstract (web summary)

Cyclobutanols are difficult structures to access given their inherent ring strain. Here, the authors present a method to form substituted cyclobutanols from a photocatalytic cascade reaction, proceeding via a 1,5- or 1,6-hydrogen atom transfer and subsequent ring closure.

## Introduction

Cyclic structures are fundamental in organic chemistry. Among these structures, cyclobutanols are useful cyclic skeletons frequently observed in natural products^1^ and widely serve as the targets for carbon−carbon bond cleavage chemistry because of the ring strain^2^. New methods for the expedient synthesis of diversified cyclobutanols would offer opportunities to related fields.

Tandem radical cyclizations have become a powerful synthetic method to generate complicated and useful carbon skeletons, featuring the formation of one or more consecutive rings in a single step^3,4^. The utinity of this strategy has been demonstrated in numerous synthesis of complex natural products, including linear- and angular-fused triquinianes^5^, steroids^6^, milbemycin^7^, prostaglandins^8^, (-)-Maoecrystal Z^9^, scholarisine A^10^, barbiturates^11^, and ophiobolin sesterterpene^12^. Visible-light photoredox chemistry is an area seeing tremendous growth as it notably provides mild reaction conditions for the generation of radicals^13–16^, rendering many elegant intramolecular^17,18^ and intermolecular [2 + 2]-^19,20^, [3 + 2]-^21,22^, [4 + 2]-^23,24^, and [2 + 2 + 2]-cyclization^25^ reactions possible using iridium (Ir)- or ruthenium (Ru)-based visible-light sensitizers.

**Fig. 1:**
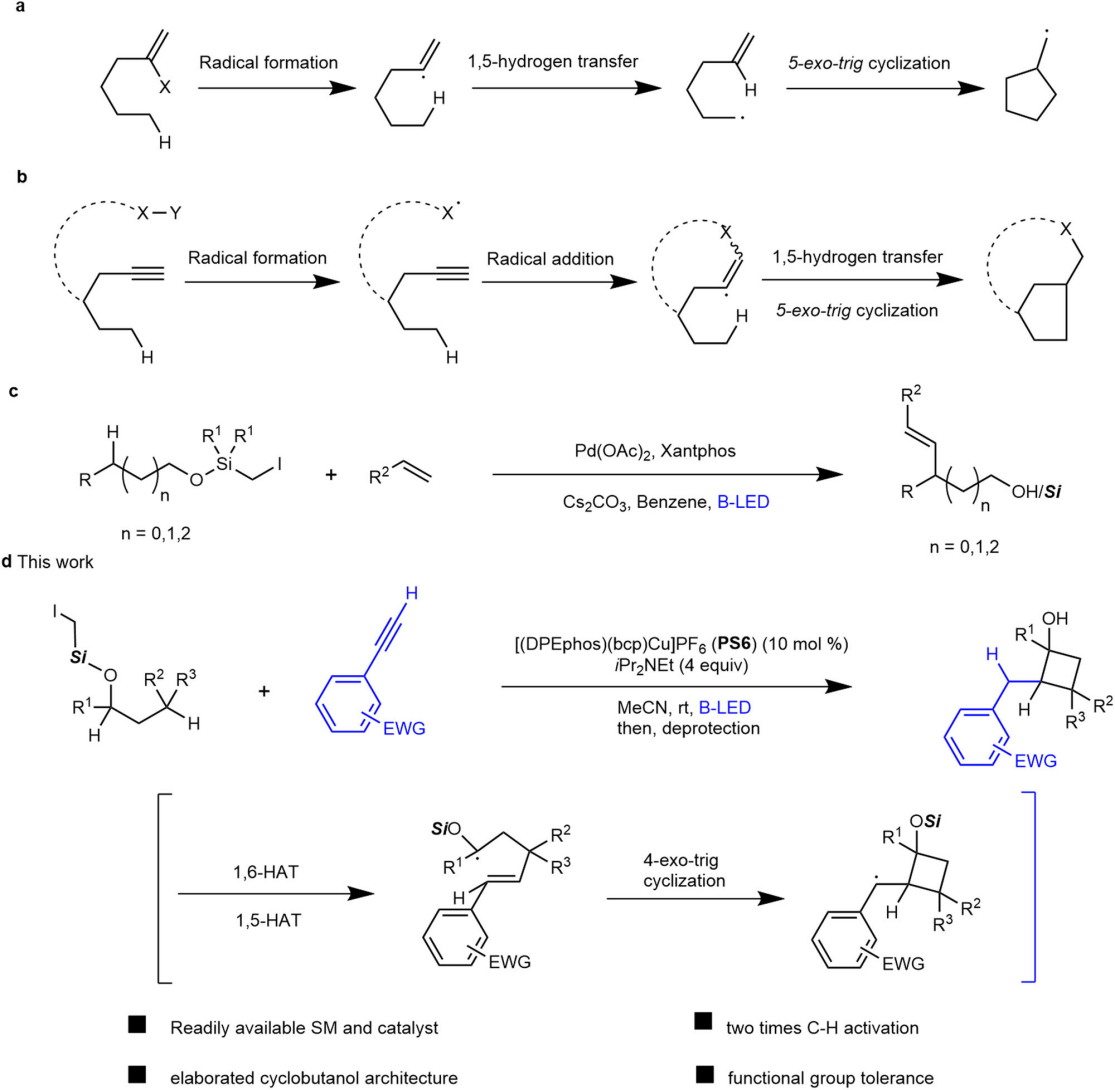
Remote C−H functionalization via HAT. **a** Vinyl radical triggered by activation of C(sp^2^)-X initiated HAT and cyclization. **b** Vinyl radical triggered by radical addition to alkyne initiated HAT and cyclization. **c** Remote C − H functionalization via HAT enabled by an iodomethylsilyl auxiliary. **d** Photoinduced copper-catalyzed sequential 1,n-HAT enabling the formation of cyclobutanols.

Vinyl radical initiated 1,*n*-hydrogen atom transfer (HAT) and cyclization is another powerful way to construct a wide range of five-membered rings, pioneered by the works of Heiba and Dessau^26^, Curran^27^, Parsons^28^, Renaud^29^, and others (Fig. 1a, b)^30–34^. In most cases, however, intramolecular versions have dominated and have necessitated that vinyl halides or pseudohalides usually require multistep synthesis. The generation of cyclic skeletons other than five-membered rings is rare. We envisioned the possibility of the in situ generation of vinyl radicals in an intermolecular manner, and if followed by HAT and cyclization, a different ring structure could potentially be furnished. The success of this hypothesis relied on a new combination of radical precursor and reactant.

For activated C − X bonds, such as polyhalogenated alkanes^35^, α-halocarbonyl compounds^36^, geminal halogenated sugars^37^, and benzyl bromides bearing strong electron-withdrawing groups^38^ have been used to generate radical intermediates under photoredox conditions. The use of substrates containing unactivated halogen−carbon bonds is less studied because of the relatively high reduction potential and putative reactivity of the resulting unstabilized *sp*^3^ carbon radical. We have been interested in copper (Cu)-photocatalysis using aliphatic halide as the radical precursor^39–41^. Cu complexes have displayed highly tunable redox properties in their excited states, diverse ligand types, and the free conversion of multiple oxidation states^42–45^. Taking advantage of the silicon effect for the generation and translocation of α-carbon radicals^27,46^, Gevorgyan recently advanced a series of works on the direct γ-amination and γ-vinylation of alcohol derivatives through 1,6-HAT using (halomethyl)silyl auxiliaries (Fig. 1c)^47–50^. Despite this progress, as far as we know, examples of the use of readily available aliphatic halides as the radical precursor under visible-light photocatalysis for intermolecular cascade cyclization en route to four-membered rings are lacking. During our investigation on photoinduced Cu-catalyzed remote C − H alkynlylation with iodomethylsilyl alcohols^39^, we isolated an unexpected cyclized compound **3a** as a minor product when we employed an electron-deficient aryl alkyne. Notably, **3a** is a product of a HAT/vinyl radical formation/cyclization cascade reaction that involves the functionalization of two C(*sp*^3^)−H bonds.

Herein, we report our preliminary results on the photoinduced Cu-catalyzed intermolecular radical cascade cyclization of iodomethylsilyl alcohols with alkynes leading to synthetically useful cyclobutanol skeletons (Fig. 1d). This method not only constitutes a valuable complement to Norrish Yang–type cyclobutanol formation, which features intramolecular reaction and usually requires an arylketone or diketone for better light absorption^51,52^, but it also contributes an example of double C(*sp*^3^)−H functionalization chemistry^53–57^.

## Results

### Reaction optimization

**Fig. 2:**
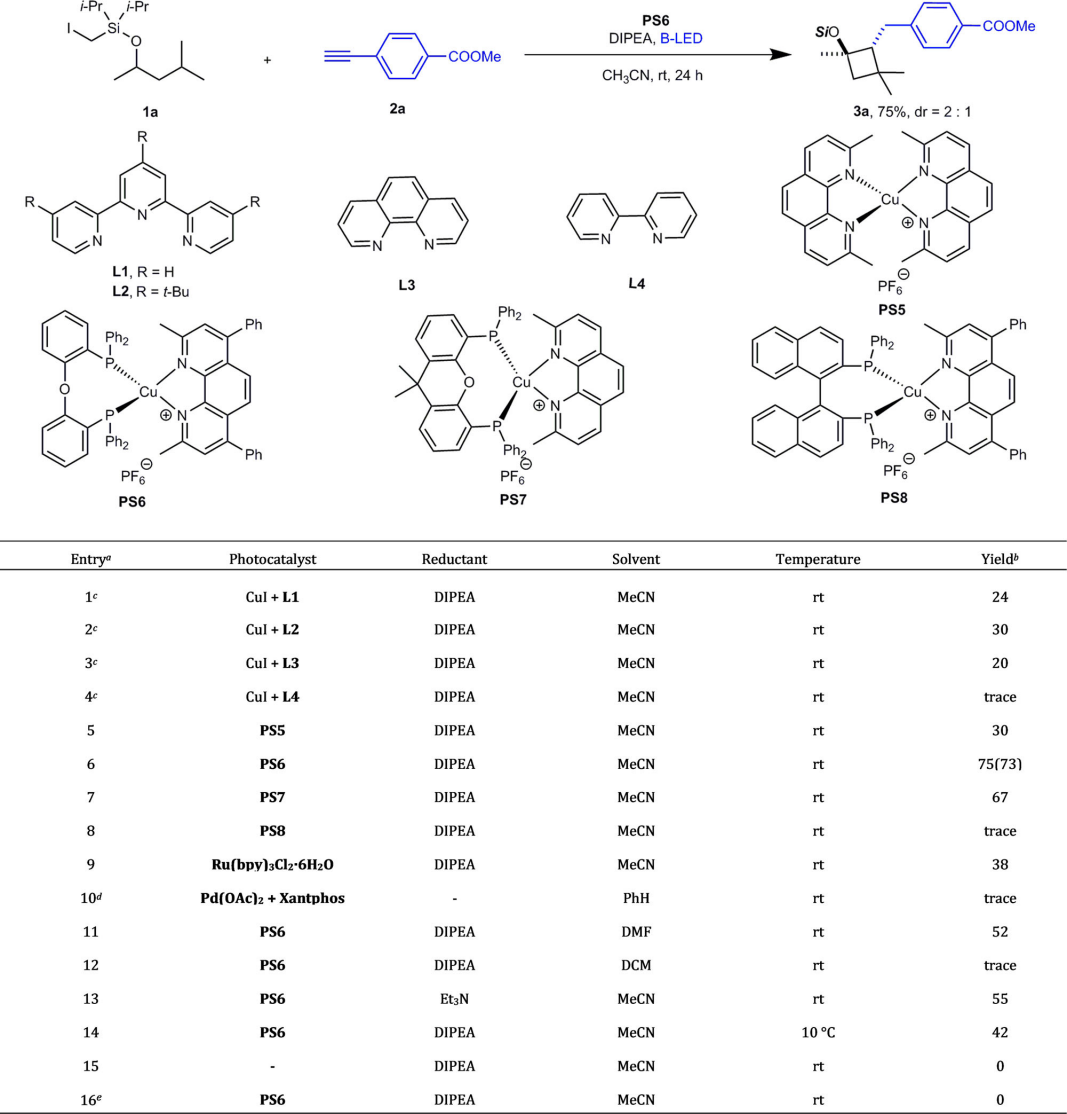
Synthesis of 3a under various reaction conditions. ^a^ **1a** (0.1 mmol), **2a** (0.15 mmol), **PS** (10 mol %), *N*, *N*-Diisopropylethylamine(DIPEA) (4 equiv) in MeCN, under N_2_, rt, B-LED, 24 h. The relative configuration was assigned by NOE analysis. ^b^ Determined by ^1^H NMR analysis with an internal standard (diethyl phthalate). The value in parentheses is the deprotected isolated yield. ^c^ CuI (10 mol%), ligand (20 mol %). ^d^ Pd(OAc)_2_ (10 mol %), Xantphos (20 mol %) and Cs_2_CO_3_ (2 equiv) in PhH, under N_2_, rt, B-LED, 24 h. ^e^ No light.

We optimized this interesting reaction and also, more importantly, explored the reaction mechanism and its scope. The key to improving the reaction was to identify the best photocatalyst and reductant. The complexes of CuI with simple bipyridine, tripyridine, or phenanthroline ligands were less effective for this reaction (Fig. 2, entries 1–4). Photocatalysis using homoleptic or heteroleptic Cu complexes experienced significant growth^42–45^. We thus prepared a series of homoleptic Cu complexes; however, the [(dmp)_2_Cu]PF_6_ complex with different substitutions provided less of the product (Fig. 2, entry 5). We then turned our attention to heteroleptic diamine/bisphosphine Cu ligands with notable features including an easily tunable chromophore and bite angle to influence the photophysical properties and catalytic activity^42,43^. Among all of the heteroleptic complexes evaluated (Fig. 2, entry 6–8), [(DPEphos)(bcp)Cu]PF_6_, originally a photosensitizer for the photocatalytic reduction of water^58^ and carbon dioxide^59^ and was recently applied in carbohalide photoactivation by Evano^60^, proved to be superior. We examined Ru-based photocatalysts for comparison; however, the cyclized product was formed in a relatively lower yield (Fig. 2, entry 9). Palladium was not a suitable catalyst for this cyclization reaction (Fig. 2, entry 10). Further optimization of the sacrificial reductant revealed that Hünig’s base was the best candidate (Fig. 2, entry 13). Lowering the temperature did not improve the product yield (Fig. 2, entry 14), while control experiments demonstrated the strict requirement of both catalyst and light for the reaction to occur (Fig. 2, entries 15 and 16).

### Substrate scope

**Fig. 3:**
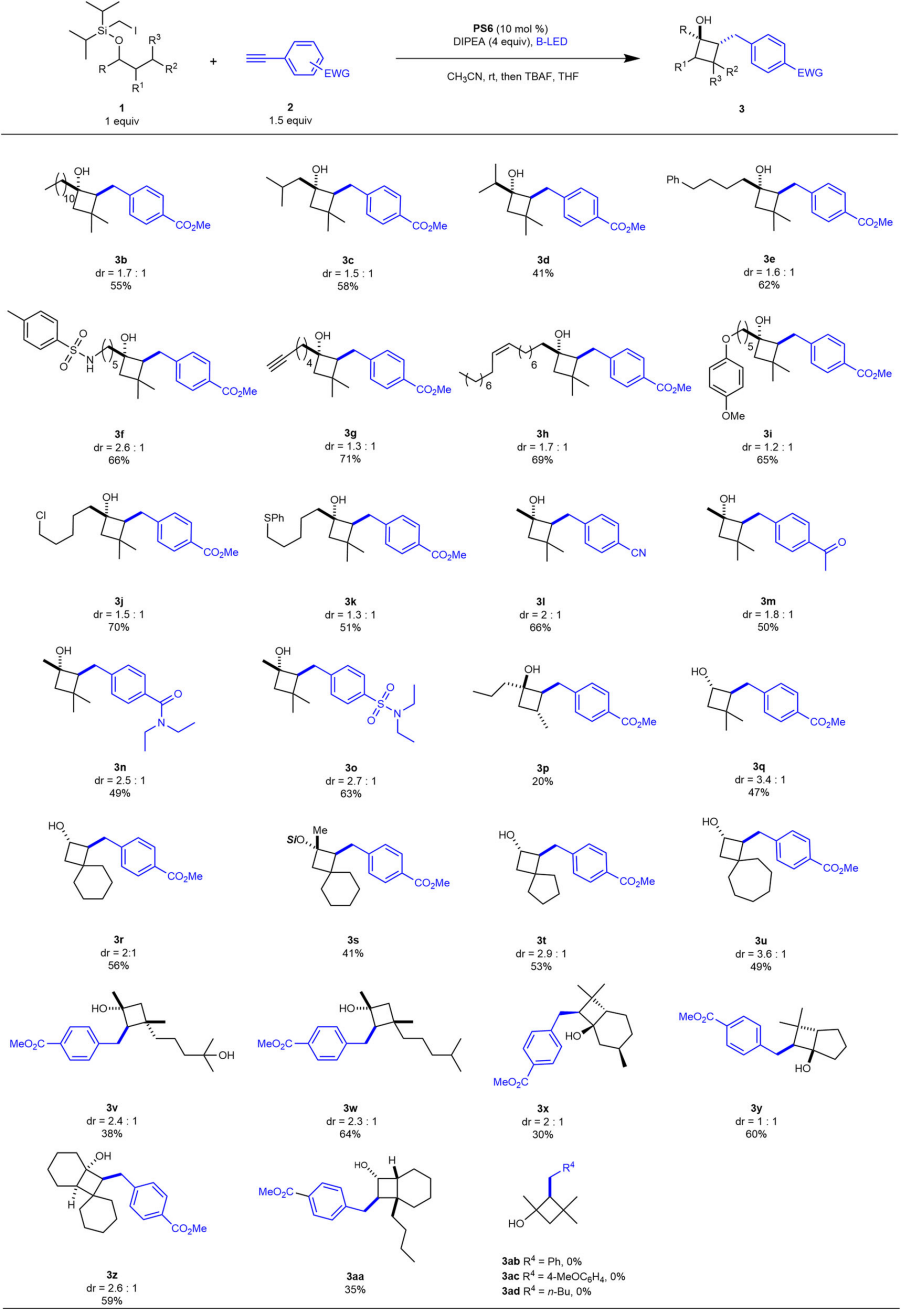
Scope of substrates considered in the study. Reaction conditions: **1** (0.1 mmol), **2** (0.15 mmol), **PS6** (10 mol %), DIPEA (4 equiv) in MeCN, under N_2_, rt, B-LED, 24 h. The relative configurations were assigned by NOE analysis; no deprotection for **3** **s**.

With these optimized conditions in mind (Fig. 2, entry 6), we next considered the scope and limitations of the light-mediated, [(DPEphos)(bcp)Cu]PF_6_-catalyzed radical cascade cyclization of aliphatic silyl iodide and alkyne-possessing representative carbon skeletons and substitution patterns (Fig. 3). Silyl alcohols with various linear or branched carbon chains were efficiently converted into the corresponding products in moderate to good yields (**3b–3d**); a phenyl group could be tolerated (**3e**). The radical nature reaction conditions were compatible with a range of functional groups, including sulfonamide (**3f**) with an acidic proton. Unsaturated C − C bonds, such as triple bonds and double bonds, were suitable at distant positions (**3g–3h**). Substrates with heteroatoms, such as oxygen, sulfur, and chloride, reacted well to provide the cyclized product in moderate yields (**3i–3k**). We also briefly explored the substrate scope with respect to substituents on phenylacetylene. Neutral or electron-donating substituents did not provide the cyclized product. Various electronic-withdrawing groups, including CN, actinium, amide, and sulfonamide, benefited the reaction, leading to the desired products in fare to moderate yields (**3l–3o**). Then, we turned our attention to another challenging aspect of this reaction—that is, the abstraction of a hydrogen atom at less-reactive secondary C(*sp*^3^)−H sites. Gratifyingly, under identical reaction conditions, cyclobutanols with three stereogenic centers formed (**3p**). Silicon-protected primary alcohol was engaged in this tandem radical cyclization, furnishing **3q** in moderate yield, suggesting that the secondary C(*sp*^3^)-H ether site could be functionalized as well. Silicon-tethered alcohols bearing a cyclic ring at the γ position reacted well, providing spiro[3.5]nonan-2-ols in moderate yields (**3r** and **3s**). Moreover, we successfully obtained 3-phenylspiro[4.4]nonan-2-ol and 3-phenylspiro[4.5]decan-2-ol with varied ring structures (**3t** and **3u**). The two substitutions at the γ position could be different (**3v** and **3w**), notably, a free hydroxyl group was compatible with the reaction conditions. We also examined the feasibility of this transformation in a more complex setting. Substrates derived from natural (-)-menthol and *trans*-2-isopropyl-cyclopentanol all furnished the radical relay [3 + 1] cyclized products (**3x**) and (**3y**). Through ingenious substrate design, we obtained more complex ring structures, such as the introduction of an extra spiral ring (**3z**) or different substitution patterns on the 4,6-fused ring system (**3aa**). Unfortunately, neither arylacetylenes with neutral or electron-donating substituents (**3ab** and **3ac**) nor alkyl alkyne (**3ad**) provided the cyclized product under standard conditions; hydrodehalogenation or the remote desaturation side products were observed instead.

### Mechanistic studies

**Fig. 4:**
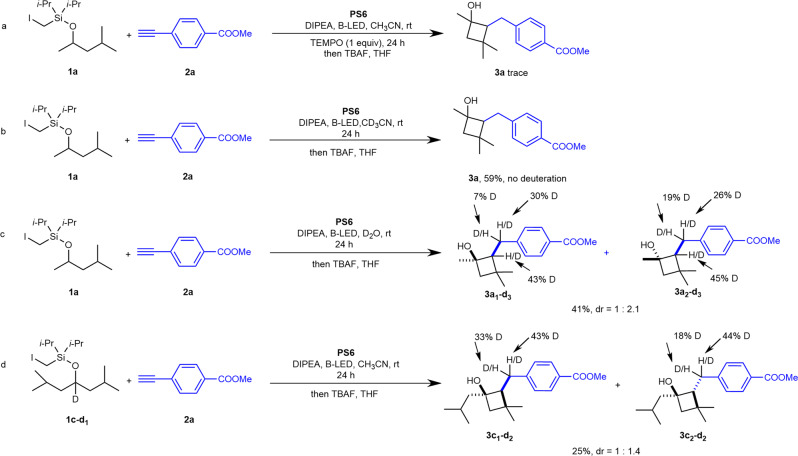
Mechanistic study. **a** Radical trapping experiment with TEMPO. **b** Deuterium experiment with CD_3_CN. **c** Deuterium experiment with D_2_O. **d** Deuterium experiment with **1c-*d1***.

To further investigate the reaction mechanism, we conducted a series of mechanistic experiments (Fig. 4). First, the addition of the radical scavenger TEMPO completely suppressed the reaction (Fig. 4a). Second, a deuterium-labeling experiment profile also supported the radical cascade cyclization hypothesis (Fig. 4b–d).

Thus, we observed 7 + 30% and 19 + 26% deuterium incorporation at the benzylic position of **3a**_**1**_**-*d3*** and **3a**_**2**_**-*d3*** when we added D_2_O instead of CD_3_CN in the reaction, suggesting that water provided the proton needed to quench the reaction. The observed 45 and 43% deuterium enrichment at the 1-positions of **3a**_**1**_**-*d3*** and **3a**_**2**_**-*d3***, respectively, was possibly due to the H/D exchange of the acetylene–hydrogen under basic conditions. Then, **1c-*d1*** with deuterium-labeled at the ether carbon provided products with 33 + 43% and 18 + 44% deuterium enrichment only at the benzylic position of **3c**_**1**_**-*d2*** and **3c**_**2**_**-*d2***, respectively, indicating the intramolecular vinyl radical (1,5-HAT). Photophysical studies have revealed that the Cu complex is a single photo-absorbing species^27^, Stern–Volmer studies demonstrated that both iodide and DIPEA could quench the excited state of the Cu complex, and the latter had a much higher efficiency (see Supplementary Information for details). According to Evano’s study, a DIPEA-intercepted Cu(I)/Cu(I)*/Cu(0) catalytic cycle^60^ might account for the reduction in organic halides to the carbon radical. Together, these results provided evidence for the photoinduced Cu-catalyzed, radical HAT/vinyl radical formation/cyclization cascade reaction process.

**Fig. 5:**
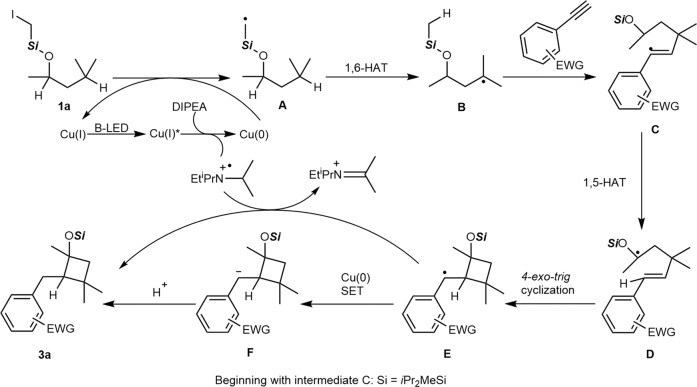
Proposed reaction mechanism. Photoredox-based dehalogenation of **1a** triggers a 1,6-HAT (**A** to **B**)/vinyl radical formation (**C**)/cyclization cascade reaction (**D**, **E**, **F**) involving the functionalization of two C − H bonds to form valuable cyclobutanols (**3a**).

According to the literature^49,60–63^ and on the basis of those findings, we proposed the following mechanism (Fig. 5). The active photoexcited [(DPEphos)(bcp)Cu]PF_6_ was reduced by DIPEA to provide a Cu(0) species, which served as the true reductant to induce the homolysis of the C − I bond of **1a**. The resulting carbon radial (**A**), upon the addition of 1,6-HAT, generated the new alkyl radical (**B**). The addition of a radical to alkyne gave rise to the vinyl radical **C**. The resulting vinyl radical **E** would go through an intramolecular reaction with 1,5-HAT to selectively activate the C (*sp*^3^)−H of ether and to afford a nucleophilic, secondary alkyl radical species **D**. A 4-*exo-trig*-type cyclization took place to afford the cyclized radical species (**E**), which was quenched to deliver the final product **3a**. Moreover, quantum yield (Φ = 0.44%) suggested that a radical-chain process might not be involved (see Supplementary Information for details).

**Fig. 6:**
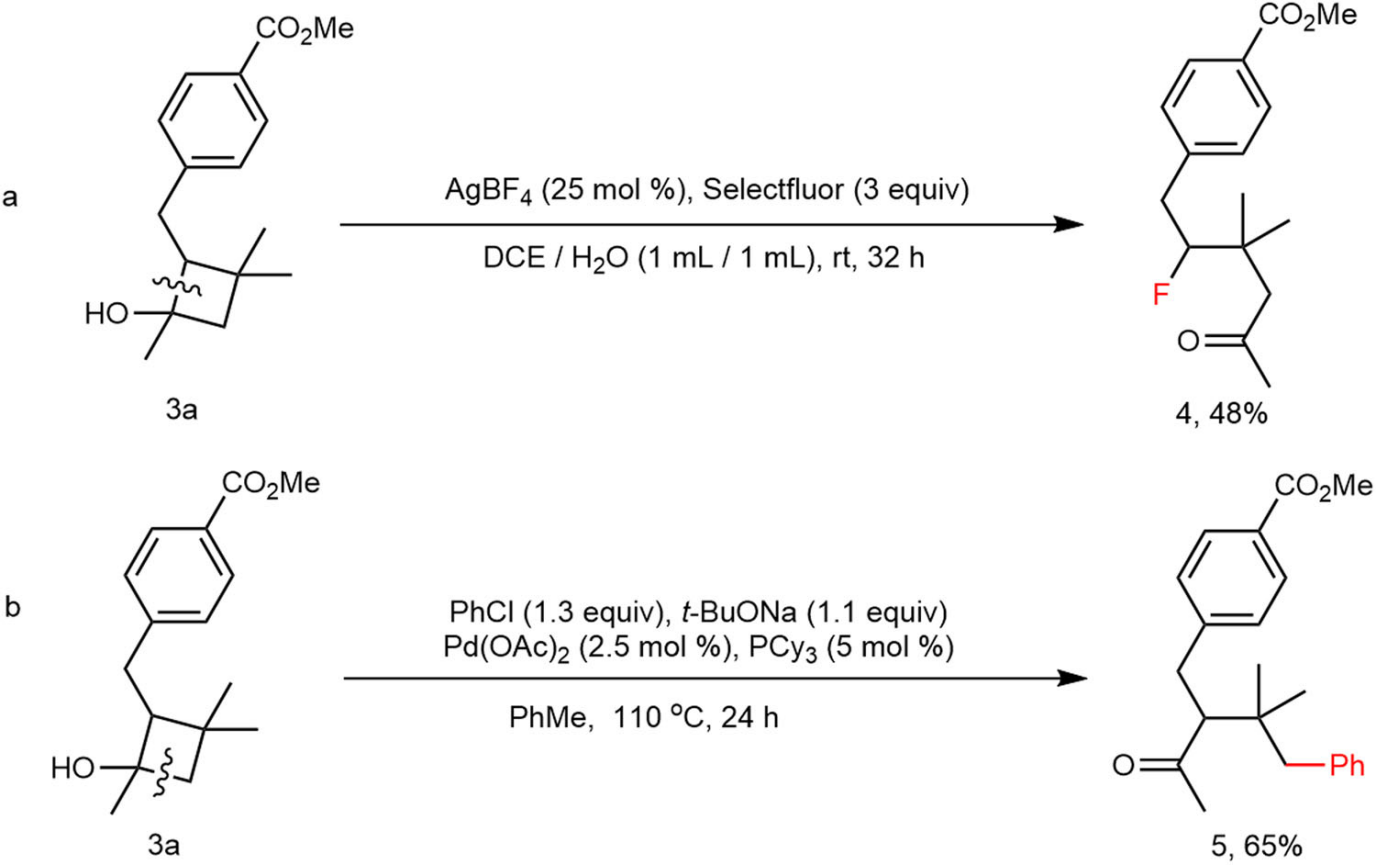
Transformations of cyclobutanols. **a** Synthesis of γ-fluoroketone **4**. **b** Synthesis of γ-arylketone **5**.

To demonstrate the synthetic potential of this methodology and identify the chemical structure of the product, we conducted two derivatizations (Fig. 6). **1a** underwent two types of ring-opening functionalization processes. We generated γ-fluoroketone through a radical pathway with a ring opening on the substituted side (Fig. 6a). In contrast, we isolated γ-arylketone under palladium catalysis with a ring opening on the nonsubstituted side (Fig. 6b).

In summary, we have shown that the exposure of silyl ether iodides to a heteroleptic Cu catalyst and visible light in the absence of exogenous photosensitizers at room temperature leads to the formation of a carbon radical species. This intermediate triggers a 1,6-HAT/vinyl radical formation/cyclization cascade reaction involving the functionalization of two C − H bonds to form valuable cyclobutanols. The radical nature reaction tolerates a broad range of functional groups and could proceed under complex settings, providing elaborate polycyclic molecules. It is expected that the discovered reactivity of intermolecular radical [3 + 1] cascade cyclization under visible-light/copper-catalyzed conditions would support the further study of new methods, and these developed methods would find applications in synthesis.

## Methods

In a dried sealed vial, [(DPEphos)(bcp)Cu]PF_6_ (0.010 mmol, 10 mol %), DIPEA (0.4 mmol, 4.0 equiv), and terminal alkyne (0.15 mmol, 1.5 equiv) were dissolved in anhydrous CH_3_CN (1.0 mL) under N_2_ atmosphere. Then, tethered alcohol (0.1 mmol, 1.0 equiv) was added. The reaction mixture was stirred at room temperature under B-LED for 24 h. The distance between the vial and the lamp was about 1–3 cm. The resulting mixture was filtered and concentrated. After that, the residual oil was dissolved in THF (1.0 mL), followed by the addition of TBAF (2 mL, 1.0 M in THF). The reaction mixture was stirred at room temperature until the reaction was completed, as monitored by TLC analysis. Then the mixture was diluted with EtOAc, washed with NaCl (aq.), and dried over with anhydrous Na_2_SO4. After filtration and concentration, the residue was purified by silica gel chromatography with petroleum ether and ethyl acetate to afford the deprotected products.

## Supplementary information

Supplementary Information

## Data Availability

Supplementary Information is available in the online version of the paper. The experimental procedures and characterization of all new compounds are provided in Supplementary Information and also from the corresponding author upon reasonable request.
