# Supplementary material for: Photo-induced copper-catalyzed sequential 1,n-HAT enabling the formation of cyclobutanols
Source: Nat Commun. 2021 Nov 4;12:6404. doi: 10.1038/s41467-021-26670-5 (PMC8569169; doi:10.1038/s41467-021-26670-5)
Supplement: Supplementary file 1 — Supplementary Information [file 41467_2021_26670_MOESM1_ESM.docx]

Supplementary Information

Photo-Induced Copper-Catalyzed Sequential 1,n-HAT Enabling the Formation of Cyclobutanols

Zhusong Cao^1,2^, Jianye Li^1^, Guozhu Zhang^1,2^*

^1^College of Chemistry, Central China Normal University (CCNU), 152 Luoyu Road, Wuhan, Hubei 430079, P. R. China.

^2^State Key Laboratory of Organometallic Chemistry, Shanghai Institute of Organic Chemistry, Center for Excellence in Molecular Synthesis, University of Chinese Academy of Sciences, Chinese Academy of Sciences, 345 Lingling Road, Shanghai 200032, P. R. China

Table of Contents

[1. Supplementary methods S3](#_Toc79590723)

[1.1 General information S3](#_Toc79590724)

[1.2 Synthesis and characterization of substrates S3](#_Toc79590725)

[1.2.1 General procedure for the synthesis of tethered alcohols S3](#_Toc79590726)

[1.2.2 Characterizations of new substrates S4](#_Toc79590727)

[1.3 Experimental details S7](#_Toc79590728)

[1.3.1 Optimization of the reaction conditions S7](#_Toc79590729)

[1.3.2 Genernal procedures and characterizations of products S8](#_Toc79590730)

[1.3.3 Characterization data of products S9](#_Toc79590731)

[1.4 Synthetic applications S31](#_Toc79590732)

[1.4.1 Synthesis of γ-fluorinated ketone 4 S31](#_Toc79590733)

[1.4.2 Synthesis of γ-Arylation ketone 5 S31](#_Toc79590734)

[1.5 Mechanistic investigations S32](#_Toc79590735)

[1.5.1 Deuterium-labeling Experiments S32](#_Toc79590736)

[1.5.2 Radical scavenger study S37](#_Toc79590737)

[1.5.3 EPR study S37](#_Toc79590738)

[1.5.4 Photophysical studies S37](#_Toc79590739)

[1.5.5 Quantum yield measurement S40](#_Toc79590740)

[1.6 Spectral data S42](#_Toc79590741)

[2. Supplementary references S110](#_Toc79590742)

1. Supplementary methods
   1. General information

NMR spectra were recorded on Varian Inova (400 MHz), Bruker (400 MHz) or Aglient (400 MHz) spectrometer. The ^1^H NMR were calibrated against the peak of tetramethylsilane at 0 ppm). The ^13^C NMR were calibrated against the peak of the CDCl_3_ at 77.16 ppm. The multiplicities ^1^H NMR spectra are abbreviated as follows: s (singlet) d (doublet) t (triplet) q (quartet) and m (multiplet). GC/MS analysis was performed on GCMS-QP2010 Plus. HRMS analyses were performed on Thermo Fisher Scientific LTQ FTICR-MS, JEOL-AccuTOF-GCv4G-GCT MS and Waters premier GC-TOF MS. All IR spectra was processed on Nicolet 380 and the method is denoted in brackets. UV/Vis absorption spectra were recorded on HITACHI U-2910. Fluorescence spectrum was performed on HITACHI F-2700. Materials: Unless stated otherwise, acetonitrile and the substrates were purchased from commercial sources without further purification. B-LED lamps (40W, Kessil A160WE tuna blue) were used for reactions. Reactions requiring inert conditions were carried out in glove box.

- 1. Synthesis and characterization of substrates

All the tethered alcohols were prepared by conventional methods.^1-2^ Alcohol precursors not commercially available were synthesized from the corresponding aldehydes. Chloro(iodomethyl)diisopropylsilane was prepared according to literature procedure.^3^

1.2.1 General procedure for the synthesis of tethered alcohols

Under nitrogen atmosphere, to a 25 mL round bottom flask was added imidazole (272 mg, 4 mmol, 2 equiv) in 5 mL CH_2_Cl_2_, Chloro(iodomethyl)diisopropylsilane (640 mg, 2.2 mmol, 1.1 equiv) and corresponding alcohol. The mixture was stirred until completion of the reaction as judged by GC/MS analysis. The mixture was then concentrated under reduced pressure. The residue was purified by column chromatography in hexanes.

1.2.2 Characterizations of new substrates

**(iodomethyl)diisopropyl((2-methyldec-9-yn-4-yl)oxy)silane**

**1g** was prepared according to general procedure in 82% isolated yield. Colorless oil. ^1^H NMR (400 MHz, CDCl_3_): δ3.98-3.89 (m, 1H), 2.19 (td, *J* = 6.8, 2.6 Hz, 2H), 2.07 (s, 2H), 1.93 (t, *J* = 2.6 Hz, 1H), 1.72-1.61 (m, 1H), 1.58-1.40 (m, 6H), 1.41-1.28 (m, 2H), 1.25-1.14 (m, 2H), 1.07 (dd, *J* = 8.7, 5.1 Hz, 12H), 0.89 (dd, *J* = 6.6, 3.3 Hz, 6H). ^13^C NMR (101 MHz, CDCl_3_): δ 84.6, 71.3, 68.4, 46.3, 36.8, 28.9, 24.8, 23.9, 23.3, 23.1, 18.6, 18.0, 18.0, 17.7, 17.7, 12.9, 12.9. IR (neat) cm^-1^ ṽ: 3312, 2940, 2865, 1463, 1369, 1248, 1141, 1101, 1078, 1043, 882, 721, 680, 627. HRMS (DART) m/z calcd. for C_18_H_36_IOSi [M+H]^+^: 423.1575; found: 423.1568.

**(2-cyclohexylethoxy)(iodomethyl)diisopropylsilane**

**1r** was prepared according to general procedure in 85% isolated yield. Colorless oil. ^1^H NMR (400 MHz, CDCl_3_): δ 3.77 (t, *J* = 6.8 Hz, 2H), 2.07 (s, 2H), 1.75-1.60 (m, 5H), 1.50-1.35 (m, 3H), 1.26-1.14 (m, 5H), 1.10-1.02 (m, 12H), 0.98-0.82 (m, 2H). ^13^C NMR (101 MHz, CDCl_3_): δ 62.0, 40.5, 34.4, 33.5, 26.8, 26.5, 17.9, 17.6, 12.4. IR (neat) cm^-1^ ṽ: 2921, 2852, 1462, 1448, 1379, 1102, 1012, 989, 882, 776, 725, 684. HRMS (FI) m/z calcd. for C_15_H_31_IOSi [M]^+^: 382.1183; found: 382.1181.

**(2-cyclopentylethoxy)(iodomethyl)diisopropylsilane**

**1t** was prepared according to general procedure in 86% isolated yield. Colorless oil. ^1^H NMR (400 MHz, CDCl_3_): δ 3.74 (t, *J* = 6.9 Hz, 2H), 2.08 (s, 2H), 1.90-1.82 (m, 1H), 1.81-1.71 (m, 2H), 1.64-1.54 (m, 4H), 1.53-1.45 (m, 2H), 1.28-1.16 (m, 2H), 1.10-1.06 (m, 2H), 1.12-1.02 (m, 14H). ^13^C NMR (101 MHz, CDCl_3_): δ 77.5, 77.2, 76.8, 63.5, 39.3, 36.7, 32.84, 25.2, 17.8, 17.6, 12.4, 11.8. IR (neat) cm^-1^ ṽ: 2943, 2865, 1462, 1382, 1253, 1099, 1010, 882, 725, 684. HRMS (DART) m/z calcd. for C_14_H_30_IOSi [M+H]^+^: 369.1105; found: 369.1102.

**(2-cycloheptylethoxy)(iodomethyl)diisopropylsilane**

**1u** was prepared according to general procedure in 80% isolated yield. Colorless oil. ^1^H NMR (400 MHz, CDCl_3_): δ 3.75 (t, *J* = 6.8 Hz, 2H), 2.07 (s, 2H), 1.74-1.34 (m, 13H), 1.28-1.12 (m, 4H), 1.10-1.04 (m, 12H). ^13^C NMR (101 MHz, CDCl_3_): δ 62.4, 41.4, 35.8, 34.8, 28.7, 26.6, 17.8, 17.6, 12.4, -20.6. IR (neat) cm^-1^ ṽ: 2920, 2863, 1460, 1381, 1099, 1064, 996, 951, 881, 808, 724, 684, 519, 444. HRMS (DART) m/z calcd. for C_16_H_34_IOSi [M+H] ^+^: 397.1418; found: 397.1412.

**8-(((iodomethyl)diisopropylsilyl)oxy)-2,6-dimethylnonan-2-ol**

**1v** was prepared according to general procedure in 52% isolated yield. Colorless oil.  ^1^H NMR (400 MHz, CDCl_3_): δ1.65-1.24 (m, 1H), 2.06 (s, 2H), 1.65-1.24 (m, 9H), 1.23-1.01 (m, 21H), 1.01-0.93 (m, 2H), 0.90-0.82 (m, 3H). ^13^C NMR (101 MHz, CDCl_3_): δ 71.1, 67.7, 67.5, 47.5, 47.5, 44.7, 44.3, 38.1, 37.9, 29.9, 29.9, 29.4, 29.4, 29.4, 29.3, 24.7, 23.9, 21.9, 21.7, 20.1, 20.0, 18.0, 18.0, 17.9, 17.7, 17., 17.67, 17.6, 12.8, 12.8, 12.7, 12.7. IR (neat) cm^-1^ ṽ: 3384, 2942, 2866, 1719, 1674, 1461, 1383, 1251, 1056, 995, 882, 815, 723, 655, 520, 440. HRMS (ESI) m/z calcd. for C_18_H_39_INaO_2_Si [M+Na]^+^: 465.1656; found: 465.1653.

**((4,8-dimethylnonan-2-yl)oxy)(iodomethyl)diisopropylsilane**

**1w** was prepared according to general procedure in 83% isolated yield. Colorless oil. ^1^H NMR (400 MHz, CDCl_3_): δ 4.11-4.02(m, 1H), 2.08 (s, 2H), 1.67-1.41 (m, 3H), 1.41-0.97 (m, 26H), 0.90-0.84(m, 9H). ^13^C NMR (101 MHz, CDCl_3_): δ 67.8, 67.6, 47.6, 47.6, 39.48, 39.4, 37.9, 37.7, 29.9, 29.4, 28.1, 28.1, 24.9, 24.8, 24.7, 23.9, 22.9, 22.9, 22.8, 22.8, 20.2, 20.1, 18.0, 18.0, 17.9, 17.7, 17.7, 17.7, 12.8, 12.8, 12.7. IR (neat) cm^-1^ ṽ: 2953, 2925, 2896, 2867, 1463, 1377, 1126, 1079, 994, 882, 721, 679. HRMS (FI) m/z calcd. for C_18_H_39_IOSi [M]^+^: 426.1812; found: 426.1812.

**([1,1'-bi(cyclohexan)]-2-yloxy)(iodomethyl)diisopropylsilane**

**1z** was prepared according to general procedure in 66% isolated yield. Colorless oil. ^1^H NMR (400 MHz, CDCl_3_): δ 3.68 (td, *J* = 9.5, 4.2 Hz, 1H), 2.08 (s, 2H), 1.98-1.79 (m, 3H), 1.78-1.54 (m, 9H), 1.46-0.85 (m, 34H). ^13^C NMR (101 MHz, CDCl_3_): δ 68.8, 48.3, 38.3, 34.9, 31.6, 30.7, 26.9, 26.8, 26.6, 26.6, 23.4, 20.4, 18.3, 18.0, 17.9, 17.7, 13.1, 13.0. IR (neat) cm^-1^ ṽ: 2922, 2850, 1462, 1446, 1371, 1090, 1062, 1045, 1018, 882, 724. HRMS (FI) m/z calcd. for C_19_H_37_IOSi [M]^+^: 436.1653; found: 436.1656.

**((2-butylcyclohexyl)methoxy)(iodomethyl)diisopropylsilane**

**1aa** was prepared according to general procedure in 73% isolated yield. Colorless oil. ^1^H NMR (400 MHz, CDCl_3_): δ 3.66 (qd, *J* = 9.8, 7.4 Hz, 2H), 2.08 (s, 2H), 1.84-1.75 (m, 1H), 1.70-1.62 (m, 1H), 1.60-1.15 (m, 16H), 1.11-1.05 (m, 12H), 0.89 (t, *J* = 7.0 Hz, 3H). ^13^C NMR (101 MHz, CDCl_3_): δ 64.4, 42.034, 36.6, 30.1, 29.0, 28.8, 25.9, 24.2, 23.5, 23.2, 17. 9, 17.6, 14.4, 12.4. IR (neat) cm^-1^ ṽ: 2922, 2860, 1461, 1378, 1115, 1086, 992, 881, 799, 725, 686, 520, 443. HRMS (DART) m/z calcd. for C_18_H_38_IOSi [M+H]^+^: 425.1731; found: 425.1727.

- 1. Experimental details
     1. Optimization of the reaction conditions

**Supplementary Table 1.** **Optimization of the reaction conditions.**

| Entry*^a^* | Variation from the "Standard Conditions" | yield %*^b^* |
| --- | --- | --- |
| 1 | none | 75(73) |
| 2*^c^* | CuI, **L1** instead of [(DPEphos)(bcp)Cu]PF_6_ [(DPEphos)(bcp)Cu]PF_6_ | 24 |
| 3*^c^* | CuI, **L2** instead of [(DPEphos)(bcp)Cu]PF_6_ | 30 |
| 4*^c^* | CuI, **L3** instead of [(DPEphos)(bcp)Cu]PF_6_ | 20 |
| 5*^c^* | CuI, **L4** instead of [(DPEphos)(bcp)Cu]PF_6_ | trace |
| 6 | [(dmp)_2_Cu]PF_6_ instead of [(DPEphos)(bcp)Cu]PF_6_ | 30 |
| 7 | [(Xantphos)(bcp)Cu]PF_6_ instead of [(DPEphos)(bcp)Cu]PF_6_ | 67 |
| 8 | [(Binap)(bcp)Cu]PF_6_ instead of [(DPEphos)(bcp)Cu]PF_6_ | trace |
| 9 | Ru(bpy)_3_Cl_2_·6H_2_O instead of [(DPEphos)(bcp)Cu]PF_6_ | 38 |
| 10 | DMF instead of MeCN | 52 |
| 11 | DCM instead of MeCN | trace |
| 12 | Et_3_N instead of *i*Pr_2_NEt | 55 |
| 13 | Reaction was performed on 10 ℃ | 42 |
| 14*^d^* | Pd(OAc)_2_, Xantphos and Cs_2_CO_3_ in PhH | trace |
| 15 | Without light or photocatalyst | 0 |

*^a^* **1a** (0.1 mmol), **2a** (0.15 mmol), PC (10 mol %), *i*Pr_2_NEt (4 equiv) in MeCN, under N_2_, rt, B-LED, 24 h. *^b^* Determined by ^1^H NMR analysis with internal standard (diethyl phthalate), the value in parentheses is the deprotected isolated yield. *^c^* CuI (10 mol %), Ligand (20 mol %). *^d^* Pd(OAc)_2_ (10 mol %), Xantphos (20 mol %) and Cs_2_CO_3_ (2 equiv.) in PhH, under N_2_, rt, B-LED, 24 h.

1.3.2 Genernal procedures and characterizations of products

In a dried sealed vial, [(DPEphos)(bcp)Cu]PF_6_ (0.010 mmol, 10 mol %), DIPEA (0.4 mmol, 4.0 equiv) and terminal alkyne (0.15 mmol, 1.5 equiv) were dissolved in CH_3_CN (1.0 mL) under N_2_ atmosphere. Then, tethered alcohol (0.1 mmol, 1.0 equiv) was added. The reaction mixture was stirred at room temperature under B-LED for 24 h. The distance between the vial and the lamp was about 1–3 cm. The resulting mixture was filtered and concentrated. After that, the residual oil was dissolved in THF (1.0 mL), followed by the addition of TBAF (2 mL, 1.0 M in THF). The reaction mixture was stirring at room temperature until the reaction was completed, as monitored by TLC analysis. Then the mixture was diluted with EtOAc, washed with saturated NaCl (aq.) and dried over with anhydrous Na_2_SO_4_. After filtration and concentration, the residue was purified by silica gel chromatography with petroleum ether and ethyl acetate afford the deprotected products.

1.3.3 Characterization data of products

**methyl 4-((2-hydroxy-2,4,4-trimethylcyclobutyl)methyl)benzoate**

**3a** was prepared according to general procedure in 73% isolated yield. dr = 1 : 2.0.

Yellow solid. ^1^H NMR (400 MHz, CDCl_3_): δ 7.94 (d, *J* = 8.3 Hz, 2H), 7.33 (d, *J* = 8.3 Hz, 2H), 3.90 (s, 3H), 2.92 (dd, *J* = 14.0, 9.5 Hz, 1H), 2.71 (dd, *J* = 13.9, 6.5 Hz, 1H), 2.19 (dd, *J* = 9.4, 6.7 Hz, 1H), 1.82 (d, *J* = 12.4 Hz, 1H), 1.75 (d, *J* = 12.4 Hz, 1H), 1.19 (s, 3H), 1.10 (s, 3H), 1.03 (s, 3H). ^13^C NMR (101 MHz, CDCl_3_): δ 167.3, 147.7, 129.8, 129.1, 127.8, 72.6, 55.0, 52.1, 49.0, 32.9, 31.3, 31.3, 31.0, 24.4. IR (neat) cm^-1^ ṽ: 3495, 2951, 2922, 2859, 1721, 1609, 1435, 1276, 1225, 1179, 1106, 1019, 758, 702. HRMS (ESI) m/z calcd. for C_16_H_23_O_3_ [M+H]^+^: 263.1642; found: 263.1642.

Yellow oil. ^1^H NMR (400 MHz, CDCl_3_): δ 7.94 (d, *J* = 8.2 Hz, 2H), 7.33 (d, *J* = 8.2 Hz, 2H), 3.89 (s, 3H), 2.81 (dd, *J* = 14.7, 7.7 Hz, 1H), 2.68 (dd, *J* = 14.8, 8.2 Hz, 1H), 2.48 (t, *J* = 7.9 Hz, 1H), 1.94-1.82 (m, 2H), 1.72 (s, 1H), 1.34 (s, 3H), 1.03 (d, *J* = 6.2 Hz, 6H). ^13^C NMR (101 MHz, CDCl_3_): δ 167.3, 146.9, 129.8, 128.9, 127.9, 71.3, 57.0, 52.1, 50.3, 32.8, 32.3, 28.9, 25.0, 23.1. IR (neat) cm^-1^ ṽ: 3463, 2954, 2924, 2862, 1721, 1610, 1435, 1278, 1181, 1108, 1019, 799, 758. HRMS (ESI) m/z calcd. for C_16_H_23_O_3_ [M+H]^+^: 263.1461; found: 263.1461.

**methyl 4-((2-hydroxy-4,4-dimethyl-2-undecylcyclobutyl)methyl)benzoate**

**3b** was prepared according to general procedure in 55% isolated yield. dr = 1 : 1.7.

Colorless oil. ^1^H NMR (400 MHz, CDCl_3_): δ 7.93 (d, *J* = 8.1 Hz, 2H), 7.31 (d, *J* = 8.1 Hz, 2H), 3.90 (s, 3H), 2.90 (dd, *J* = 14.1, 8.8 Hz, 1H), 2.74 (dd, *J* = 14.0, 7.2 Hz, 1H), 2.19 (t, *J* = 7.8 Hz, 1H), 1.73 (dd, *J* = 57.8, 12.4 Hz, 2H), 1.61 (s, 1H), 1.40-1.06 (m, 23H), 1.00 (s, 3H), 0.87 (t, *J* = 6.8 Hz, 3H). ^13^C NMR (101 MHz, CDCl_3_): δ 167.3, 147.8, 129.8, 129.1, 127.7, 75.0, 54.1, 52.1, 47.4, 43.9, 32.9, 32.1, 31.5, 31.4, 30.0, 29.8, 29.8, 29.7, 29.5, 24.7, 23.5, 22.9, 14.3. IR (neat) cm^-1^ ṽ: 3516, 2923, 2853, 1724, 1708, 1610, 1463, 1435, 1277, 1179, 1108, 1020, 759, 702. HRMS (ESI) m/z calcd. for C_26_H_42_NaO_3_ [M+Na]^+^: 425.3026; found: 425.3028.

Colorless oil. ^1^H NMR (400 MHz, CDCl_3_): δ 7.93 (d, *J* = 8.2 Hz, 2H), 7.32 (d, *J* = 8.2 Hz, 2H), 3.88 (s, 3H), 2.86 (dd, *J* = 14.8, 7.5 Hz, 1H), 2.67 (dd, *J* = 14.8, 8.5 Hz, 1H), 2.52 (t, *J* = 8.0 Hz, 1H), 1.85 (dd, *J* = 93.0, 12.2 Hz, 2H), 1.69 (s, 1H), 1.63-1.47 (m, 1H), 1.27 (d, *J* = 15.1 Hz, 18H), 1.00 (d, *J* = 3.0 Hz, 6H), 0.87 (t, *J* = 6.7 Hz, 3H). ^13^C NMR (101 MHz, CDCl_3_): δ 167.29, 147.16, 129.76, 128.88, 127.79, 73.52, 57.39, 52.10, 47.53, 37.02, 32.85, 32.05, 31.80, 30.33, 29.83, 29.79, 29.77, 29.74, 29.48, 29.00, 23.64, 22.83, 22.57, 14.26. IR (neat) cm^-1^ ṽ: 3488.34, 2922.37, 2852.52, 1723.36, 1706.14, 1609.62, 1462.63, 1435.09, 1276.18, 1179.69, 1108.19, 1019.45, 758.22. HRMS (ESI) m/z calcd. for C_26_H_42_NaO_3_ [M+Na]^+^: 425.3026; found: 425.3027.

**methyl 4-((2-hydroxy-2-isobutyl-4,4-dimethylcyclobutyl)methyl)benzoate**

**3c** was prepared according to general procedure in 58% isolated yield. dr = 1 : 1.5.

Colorless oil. ^1^H NMR (400 MHz, CDCl_3_): δ 7.93 (d, *J* = 8.1 Hz, 2H), 7.30 (d, *J* = 8.1 Hz, 2H), 3.90 (s, 3H), 2.88 (dd, *J* = 13.9, 7.9 Hz, 1H), 2.74 (dd, *J* = 14.1, 7.7 Hz, 1H), 2.16 (t, *J* = 7.9 Hz, 1H), 1.88 (d, *J* = 12.5 Hz, 1H), 1.77-1.56 (m, 2H), 1.33-1.20 (m, 2H), 1.19 (s, 3H), 0.97 (s, 6H). ^13^C NMR (101 MHz, CDCl_3_): δ 167.4, 147.7, 129.8, 129.1, 127.8, 75.13, 6.19, 52.2, 52.1, 48.9, 33.8, 31.5, 31.2, 24.5, 24.5, 24.5, 23.3. IR (neat) cm^-1^ ṽ: 3519, 2951, 2925, 2868, 1722, 1708, 1610, 1465, 1436, 1367, 1279, 1179, 1110, 1019, 800, 759. HRMS (ESI) m/z calcd. for C_19_H_28_NaO_3_ [M+Na]^+^: 327.1931; found: 327.1928.

White solid. ^1^H NMR (400 MHz, CDCl_3_): δ 7.94 (d, *J* = 8.2 Hz, 2H), 7.32 (d, *J* = 8.1 Hz, 2H), 3.89 (s, 3H), 2.84 (dd, *J* = 14.8, 7.2 Hz, 1H), 2.66 (dd, *J* = 14.7, 8.8 Hz, 1H), 2.49 (t, *J* = 8.0 Hz, 1H), 2.10 (d, *J* = 12.3 Hz, 1H), 1.90-1.78 (m, 2H), 1.58 (dd, *J* = 14.3, 6.7 Hz, 1H), 1.48 (dd, *J* = 14.4, 6.2 Hz, 1H), 1.01 (s, 3H), 0.99 (s, 3H), 0.96 (dd, *J* = 6.6, 4.0 Hz, 6H). ^13^C NMR (101 MHz, CDCl_3_): δ 167.3, 147.0, 129.8, 128.9, 127.9, 73.4, 57.6, 52.1, 47.6, 45.2, 36.9, 32.9, 32.8, 31.8, 29.0, 27.6, 23.6, 22.0. IR (neat) cm^-1^ ṽ: 3483, 2949, 2861, 1720, 1609, 1435, 1278, 1180, 1107, 1019, 981, 759, 723, 702. HRMS (ESI) m/z calcd. for C_19_H_32_NO_3_ [M+NH_4_]^+^: 322.2377; found: 322.2371.

**methyl 4-((2-hydroxy-2-isopropyl-4,4-dimethylcyclobutyl)methyl)benzoate**

**3d** was prepared according to general procedure in 41% isolated yield.

Colorless oil. ^1^H NMR (400 MHz, CDCl_3_): δ 7.94 (d, *J* = 8.2 Hz, 2H), 7.32 (d, *J* = 8.1 Hz, 2H), 3.90 (s, 3H), 3.07 (dd, *J* = 14.7, 5.4 Hz, 1H), 2.75 (dd, *J* = 14.7, 10.9 Hz, 1H), 2.58 (dd, *J* = 10.8, 5.5 Hz, 1H), 2.05-1.91 (m, 2H), 1.72 (d, *J* = 12.4 Hz, 1H), 1.00 – 0.84 (m, 12H). ^13^C NMR (101 MHz, CDCl_3_): δ 167.3, 147.0, 129.7, 128.9, 127.8, 76.6, 56.4, 52.1, 47.6, 33.3, 33.1, 32.6, 29.2, 24.2, 17.3, 16.4. IR (neat) cm^-1^ ṽ: 3507, 2954, 2872, 1721, 1707, 1610, 1435, 1277, 1179, 1106, 1018, 966, 799, 773, 754, 736. HRMS (ESI) m/z calcd. for C_18_H_26_NaO_3_ [M+Na]^+^: 313.1774; found: 313.1774.

**methyl 4-((2-hydroxy-4,4-dimethyl-2-(4-phenylbutyl)cyclobutyl)methyl)benzoate**

**3e** was prepared according to general procedure in 62% isolated yield. dr = 1 : 1.6.

Colorless oil. ^1^H NMR (400 MHz, CDCl_3_): δ 7.94 (d, *J* = 8.3 Hz, 2H), 7.29 (d, *J* = 8.3 Hz, 2H), 7.27-7.23 (m, 2H), 7.19-7.10 (m, 3H), 3.90 (s, 3H), 2.89 (dd, *J* = 14.0, 8.7 Hz, 1H), 2.73 (dd, *J* = 14.0, 7.1 Hz, 1H), 2.59-2.46 (m, 1H), 2.18 (t, *J* = 7.9 Hz, 1H), 1.80 (d, *J* = 12.4 Hz, 1H), 1.66 (d, *J* = 12.4 Hz, 1H), 1.51 (dt, *J* = 15.4, 7.5 Hz, 2H), 1.39 – 1.30 (m, 2H), 1.28-1.15 (m, 5H), 1.00 (s, 3H). ^13^C NMR (101 MHz, CDCl_3_): δ 167.3, 147.8, 142.6, 129.8, 129.1, 128.5, 128.4, 127.8, 125.8, 74.9, 54.2, 52.1, 47.4, 43.7, 36.0, 32.9, 31.7, 31.5, 31.4, 24.7, 23.1. IR (neat) cm^-1^ ṽ: 3518, 2928, 2858, 1721, 1609, 1436, 1280, 1179, 1109, 1020, 800, 760, 700. HRMS (ESI) m/z calcd. for C_25_H_32_NaO_3_ [M+Na]^+^: 403.2244; found: 403.2244.

Colorless oil. ^1^H NMR (400 MHz, CDCl_3_): δ 7.94 (d, *J* = 8.3 Hz, 2H), 7.32 (d, *J* = 8.3 Hz, 2H), 7.30-7.25 (m, 2H), 7.21-7.14 (m, 3H), 3.90 (s, 3H), 2.85 (dd, *J* = 14.8, 7.6 Hz, 1H), 2.74-2.59 (m, 3H), 2.52 (t, *J* = 8.0 Hz, 1H), 1.96 (d, *J* = 12.2 Hz, 1H), 1.73 (d, *J* = 12.5 Hz, 1H), 1.70-1.60 (m, 4H), 1.48-1.36 (m, 2H), 1.02 (s, 3H). ^13^C NMR (101 MHz, CDCl_3_): δ 167.3, 147.1, 142.6, 129.8, 128.9, 128.5, 128.4, 127.8, 125.8, 73.5, 57.4, 52.1, 47.5, 36.8, 36.1, 32.8, 32.1, 31.8, 29.0, 23.6, 22.3. IR (neat) cm^-1^ ṽ: 3491, 2927, 2859, 1720, 1705, 1609, 1435, 1278, 1180, 1109, 1058, 1019, 758, 699. HRMS (ESI) m/z calcd. for C_25_H_32_NaO_3_ [M+Na]^+^: 403.2244; found: 403.2243.

**Methyl 4-((2-hydroxy-4,4-dimethyl-2-(5-((4-methylphenyl)sulfonamido)pentyl)**

**cyclobutyl)methyl)benzoate**

**3f** was prepared according to general procedure in 66% isolated yield. dr = 1 : 2.4.

Colorless oil. ^1^H NMR (400 MHz, CDCl_3_): δ 7.92 (d, *J* = 8.3 Hz, 1H), 7.73 (d, *J* = 8.3 Hz, 1H), 7.30 (d, *J* = 2.2 Hz, 1H), 7.28 (d, *J* = 2.0 Hz, 1H), 4.40 (t, *J* = 6.1 Hz, 1H), 3.90 (s, 3H), 2.91-2.82 (m, 3H), 2.72 (dd, *J* = 14.0, 6.9 Hz, 1H), 2.42 (s, 3H), 2.15 (dd, *J* = 8.7, 7.1 Hz, 1H), 1.76 (d, *J* = 12.4 Hz, 1H), 1.64 (d, *J* = 12.4 Hz, 1H), 1.39-1.30 (dd, *J* = 14.6, 7.5 Hz, 2H), 1.29-1.20 (m, 2H), 1.18 (s, 3H), 1.16-1.02 (m, 4H), 1.00 (s, 3H). ^13^C NMR (101 MHz, CDCl_3_) δ 167.4, 147.8, 143.5, 137.1, 129.8, 129.8, 129.2, 127.8, 127.2, 74.8, 54.3, 52.2, 47.4, 43.6, 43.2, 32.9, 31.4, 31.4, 29.7, 26.7, 24.6, 22.9, 21.7. IR (neat) cm^-1^ ṽ: 3516, 3278, 2925, 1705, 1608, 1434, 1322, 1279, 1180, 1156, 1094, 1019, 814, 760, 704, 662, 572, 550. HRMS (ESI) m/z calcd. for C_27_H_37_NNaO_5_S [M+Na]^+^: 510.2285; found: 510.2289.

Colorless oil. ^1^H NMR (400 MHz, CDCl_3_): δ 7.93 (d, *J* = 8.2 Hz, 2H), 7.74 (d, *J* = 8.2 Hz, 2H), 7.31 (d, *J* = 7.9 Hz, 2H), 7.28 (d, *J* = 6.5 Hz, 2H), 4.75 (t, *J* = 6.0 Hz, 1H), 3.89 (s, 3H), 2.93 (dd, *J* = 13.2, 6.6 Hz, 1H), 2.83 (dd, *J* = 14.8, 7.4 Hz, 1H), 2.66 (dd, *J* = 14.8, 8.5 Hz, 1H), 2.51 (t, *J* = 7.9 Hz, 1H), 2.42 (s, 3H), 1.92 (d, *J* = 12.2 Hz, 1H), 1.72 (d, *J* = 12.4 Hz, 1H), 1.63-1.41 (m, 4H), 1.35-1.25 (m, 4H), 1.00 (d, *J* = 1.6 Hz, 6H). ^13^C NMR (101 MHz, CDCl_3_): δ 167.3, 147.0, 143.5, 137.1, 129.8, 129.8, 128.9, 127.8, 127.2, 73.4, 57.6, 52.1, 47.5, 43.2, 36.7, 32.8, 31.8, 29.7, 29.0, 27.1, 23.6, 22.1, 21.6. IR (neat) cm^-1^ ṽ: 3494, 3278, 2927, 1704, 1608, 1435, 1320, 1279, 1181, 1155, 1093, 1019, 814, 759, 730, 704, 660, 571, 550. HRMS (ESI) m/z calcd. for C_27_H_37_NNaO_5_S [M+Na]^+^: 510.2285; found: 510.2282.

**methyl 4-((2-(hex-5-yn-1-yl)-2-hydroxy-4,4-dimethylcyclobutyl)methyl)benzoate**

**3g** was prepared according to general procedure in 71% isolated yield. dr = 1 : 1.3.

Colorless oil. ^1^H NMR (400 MHz, CDCl_3_): δ 7.94 (d, *J* = 8.3 Hz, 2H), 7.31 (d, *J* = 8.2 Hz, 2H), 3.90 (s, 3H), 2.91 (dd, *J* = 14.0, 8.7 Hz, 1H), 2.74 (dd, *J* = 14.0, 7.1 Hz, 1H), 2.20 ((t, *J* = 7.6 Hz, 1H), 2.11 (td, *J* = 7.0, 2.6 Hz, 2H), 1.92 (t, *J* = 2.6 Hz, 1H), 1.82 (d, *J* = 12.4 Hz, 1H), 1.68 (d, *J* = 12.4 Hz, 1H), 1.42 (dt, *J* = 14.2, 7.1 Hz, 2H), 1.36-1.23 (m, 4H), 1.19 (s, 3H), 1.01 (s, 3H). ^13^C NMR (101 MHz, CDCl_3_): δ 167.3, 147.7, 129.8, 129.1, 127.8, 84.5, 74.8, 68.5, 54.2, 52.1, 47.4, 43.3, 32.9, 31.4, 31.4, 28.6, 24.6, 22.6, 18.5. IR (neat) cm^-1^ ṽ: 3511, 3306, 2946, 2861, 1719, 1609, 1435, 1279, 1180, 1109, 1019, 760, 632. HRMS (ESI) m/z calcd. for C_21_H_28_NaO_3_ [M+Na]^+^: 351.1931; found: 351.1932.

Colorless oil. ^1^H NMR (400 MHz, CDCl_3_): δ 7.94 (d, *J* = 8.3 Hz, 2H), 7.32 (d, *J* = 8.2 Hz, 2H), 3.90 (d, *J* = 4.5 Hz, 3H), 2.86 (dd, *J* = 14.8, 7.5 Hz, 1H), 2.68 (dd, *J* = 14.8, 8.5 Hz, 1H), 2.52 (t, *J* = 8.0 Hz, 1H), 2.22 (td, *J* = 6.8, 2.6 Hz, 1H), 1.99 (d, *J* = 10.4 Hz, 1H), 1.94 (t, *J* = 2.6 Hz, 1H), 1.76 (t, *J* = 14.7 Hz, 1H), 1.70-1.42 (m, 7H), 1.02 (d, *J* = 1.8 Hz, 6H). ^13^C NMR (101 MHz, CDCl_3_): δ 167.3, 147.0, 129.8, 128.9, 127.9, 84.5, 73.5, 68.6, 57.6, 52.1, 47.6, 36.4, 32.9, 31.8, 29.0, 28.9, 23.6, 21.8, 18.6. IR (neat) cm^-1^ ṽ: 3499, 3305, 2949, 2862, 1720, 1610, 1435, 1280, 1181, 1108, 1019, 801, 760, 633. HRMS (ESI) m/z calcd. for C_21_H_28_NaO_3_ [M+Na]^+^: 351.1931; found: 351.1933.

**Methyl 4-((2-(heptadec-8-en-1-yl)-2-hydroxy-4,4-dimethylcyclobutyl)methyl)ben**

**-zoate**

**3h** was prepared according to general procedure in 69% isolated yield. dr = 1 : 1.7.

Colorless oil. ^1^H NMR (400 MHz, CDCl_3_): δ 7.93 (d, *J* = 8.2 Hz, 2H), 7.30 (d, *J* = 8.2 Hz, 2H), 5.43-5.23 (m, 2H), 3.89 (s, 3H), 2.89 (dd, *J* = 14.0, 8.6 Hz, 1H), 2.74 (dd, *J* = 14.1, 7.1 Hz, 1H), 2.19 (t, *J* = 7.9 Hz, 1H), 2.04-1.94 (m, 4H), 1.80 (d, *J* = 12.4 Hz, 1H), 1.66 (d, *J* = 12.4 Hz, 1H), 1.39-1.09 (m, 24H), 1.03-0.96 (m, 6H), 0.87 (t, *J* = 6.8 Hz, 3H). ^13^C NMR (101 MHz, CDCl_3_): δ 167.3, 147.8, 130.1, 130.0, 129.8, 129.1, 127.8, 74.9, 54.1, 52.1, 47.4, 43.9, 32.9, 32.0, 31.5, 31.4, 30.0, 29.9, 29.9, 29.7, 29.7, 29.5, 29.3, 27.4, 27.3, 24.6, 23.5, 22.8, 14.3. IR (neat) cm^-1^ ṽ: 3512, 2922, 2853, 1724, 1709, 1609, 1462, 1435, 1276, 1178, 1108, 1020, 759, 7219, 702. HRMS (ESI) m/z calcd. for C_32_H_52_NaO_3_ [M+Na]^+^: 507.3804; found: 507.3809.

Colorless oil. ^1^H NMR (400 MHz, CDCl_3_): δ 7.93 (d, *J* = 8.2 Hz, 2H), 7.32 (d, *J* = 8.2 Hz, 2H), 5.41-5.28 (m, 2H), 3.89 (s, 3H), 2.86 (dd, *J* = 14.8, 7.4 Hz, 1H), 2.68 (dd, *J* = 14.8, 8.6 Hz, 1H), 2.52 (t, *J* = 8.0 Hz, 1H), 2.04-1.94 (m, 5H), 1.74 (d, *J* = 12.2 Hz, 1H), 1.65-1.53 (m, 2H), 1.40-1.20 (m, 22H), 1.01 (d, *J* = 3.2 Hz, 6H), 0.87 (t, *J* = 6.7 Hz, 3H). ^13^C NMR (101 MHz, CDCl_3_): δ 167.3, 147.2, 130.1, 129.9, 129.8, 128.9, 127.8, 73.5, 57.4, 52.1, 47.6, 37.0, 32.9, 32.0, 31.8, 30.3, 29.9, 29.9, 29.7, 29.7, 29.5, 29.4, 29.0, 27.4, 27.3, 23.6, 22.8, 22.6, 14.3. IR (neat) cm^-1^ ṽ: 3493, 2923, 2853, 1724, 1706, 1610, 1462, 1435, 1277, 1180, 1108, 1020, 979, 758, 721 701. HRMS (ESI) m/z calcd. for C_32_H_52_NaO_3_ [M+Na]^+^: 507.3809; found: 507.3802.

**Methyl 4-((2-hydroxy-2-(5-(4-methoxyphenoxy)pentyl)-4,4-dimethylcyclobutyl)-**

**methyl)benzoate**

**3i** was prepared according to general procedure in 65% isolated yield. dr = 1 : 1.2.

Colorless oil. ^1^H NMR (400 MHz, CDCl_3_): δ 7.93 (d, *J* = 8.1 Hz, 2H), 7.30 (d, *J* = 8.1 Hz, 2H), 6.86-6.76 (m, 4H), 3.89 (s, 3H), 3.84 (t, *J* = 6.4 Hz, 2H), 3.76 (s, 3H), 2.90 (dd, *J* = 14.1, 8.7 Hz, 1H), 2.74 (dd, *J* = 14.0, 7.0 Hz, 1H), 2.20 (t, *J* = 7.8 Hz, 1H), 1.81 (d, *J* = 12.4 Hz, 1H), 1.71-1.62 (m, 3H), 1.40-1.21 (m, 6H), 1.19 (s, 3H), 1.01 (s, 3H). ^13^C NMR (101 MHz, CDCl_3_): δ 167.3 , 153.8 , 153.3, 147.8, 129.8, 129.1, 127.8, 115.5, 114.7, 74.9, 68.5, 55.9, 54.2, 52.1, 47.4, 43.8, 32.9, 31.5, 31.4, 29.5, 26.4, 24.6, 23.3. IR (neat) cm^-1^ ṽ: 3514, 2944, 2860, 1719, 1609, 1507, 1465, 1436, 1278, 1229, 1179, 1107, 1039, 1020, 824, 760, 703. HRMS (ESI) m/z calcd. for C_27_H_36_NaO_5_ [M+Na]^+^: 463.2455; found: 463.2456.

Colorless oil. ^1^H NMR (400 MHz, CDCl_3_): δ 7.94 (d, *J* = 8.2 Hz, 1H), 7.33 (d, *J* = 8.2 Hz, 1H), 6.83 (s, 2H), 3.95-3.85 (m, 2H), 3.76 (s, 1H), 2.86 (dd, *J* = 14.8, 7.5 Hz, 1H), 2.68 (dd, *J* = 14.8, 8.4 Hz, 1H), 2.53 (t, *J* = 8.0 Hz, 1H), 1.98 (d, *J* = 12.3 Hz, 1H), 1.83 -1.71 (m, 2H), 1.71-1.53 (m, 1H), 1.52-1.37 (m, 2H), 1.02 (d, *J* = 1.2 Hz, 3H). ^13^C NMR (101 MHz, CDCl_3_): δ 167.3, 153.8, 153.3, 147.1, 129.8, 128.9, 127.8, 115.5, 114.7, 73.4, 68.6, 57.5, 55.8, 52.1, 47.5, 36.9, 32.8, 31.8, 29.6, 29.0, 26.8, 23.6, 22.4. IR (neat) cm^-1^ ṽ: 3494, 2946, 2861, 1719, 1609, 1506, 1464, 1435, 1278, 1228, 1179, 1107, 1037, 1020, 823, 758. HRMS (DART) m/z calcd. for C_27_H_40_NO_5_ [M+NH_4_]^+^: 458.2901; found: 458.2894.

**methyl 4-((2-(5-chloropentyl)-2-hydroxy-4,4-dimethylcyclobutyl)methyl)benzoate**

**3j** was prepared according to general procedure in 70% isolated yield. dr = 1 : 1.5.

Colorless oil. ^1^H NMR (400 MHz, CDCl_3_): δ 7.93 (d, *J* = 8.2 Hz, 2H), 7.30 (d, *J* = 8.1 Hz, 2H), 3.90 (s, 3H), 3.45 (t, *J* = 6.7 Hz, 2H), 2.90 (dd, *J* = 14.1, 8.9 Hz, 1H), 2.73 (dd, *J* = 14.2, 6.9 Hz, 1H), 2.18 (t, *J* = 7.6 Hz, 2H), 1.80 (d, *J* = 12.4 Hz, 1H), 1.71-1.62 (m, 3H), 1.37-1.23 (m, 4H), 1.20-1.08 (m, 5H), 1.01 (s, 3H). ^13^C NMR (101 MHz, CDCl_3_): δ 167.3, 147.7, 129.8, 129.1, 127.8, 74.8, 54.2, 52.6, 47.3, 45.1, 43.7, 32.9, 32.7, 31.4, 31.4, 27.2, 24.6, 22.8. IR (neat) cm^-1^ ṽ: 3513, 2927, 2859, 1720, 1609, 1435, 1278, 1179, 1109, 1019, 760. HRMS (ESI) m/z calcd. for C_20_H_29_ClNaO_3_ [M+Na]^+^: 375.1697; found: 375.1698.

White solid. ^1^H NMR (400 MHz, CDCl_3_): δ 7.94 (d, *J* = 8.2 Hz, 2H), 7.32 (d, *J* = 8.1 Hz, 2H), 3.89 (s, 3H), 3.54 (t, *J* = 6.7 Hz, 2H), 2.85 (dd, *J* = 14.8, 7.5 Hz, 1H), 2.68 (dd, *J* = 14.8, 8.5 Hz, 1H), 2.52 (t, *J* = 8.0 Hz, 1H), 1.97 (d, *J* = 12.3 Hz, 1H), 1.82-1.70 (m, 3H), 1.67-1.53 (m, 2H), 1.51-1.33 (m, 4H), 1.01 (s, 6H). ^13^C NMR (101 MHz, CDCl_3_): δ 167.3, 147.0, 129.8, 128.9, 127.9, 73.4, 57.6, 52.1, 47.6, 45.2, 36.9, 32.9, 32.8, 31.8, 29.0, 27.6, 23.6, 22.0. IR (neat) cm^-1^ ṽ: 3532, 3494, 2950, 2924, 2866, 1701, 1607, 1435, 1311, 1283, 1178, 1106, 1018, 757, 516. HRMS (ESI) m/z calcd. for C_20_H_33_ClNO_3_ [M+Na]^+^: 370.2143; found: 370.2139.

**Methyl 4-((2-hydroxy-4,4-dimethyl-2-(5-(phenylthio)pentyl)cyclobutyl)methyl)-benzoate**

**3k** was prepared according to general procedure in 51% isolated yield. dr = 1 : 1.3.

Colorless oil. ^1^H NMR (400 MHz, CDCl_3_): δ 7.93 (d, *J* = 8.2 Hz, 2H), 7.37-7.23 (m, 6H), 7.16 (dd, *J* = 9.2, 4.4 Hz, 1H), 3.90 (s, 3H), 3.00-2.80 (m, 3H), 2.73 (dd, *J* = 14.0, 7.0 Hz, 1H), 2.21-2.15 (m, 1H), 1.79 (d, *J* = 12.4 Hz, 1H), 1.65 (d, *J* = 12.5 Hz, 1H), 1.63-1.52 (m, 4H), 1.37-1.24 (m, 4H), 1.18 (s, 3H), 1.00 (s, 3H). ^13^C NMR (101 MHz, CDCl_3_): δ 167.3, 147.7, 137.0, 129.8, 129.1, 129.0, 129.0, 127. 8, 125.8, 74.8, 54.2, 52.1, 47.3, 43.6, 33.5, 32.9, 31.4, 31.4, 29.2, 29.1, 24.6, 23.0. IR (neat) cm^-1^ ṽ: 3516, 2926, 2857, 1720, 1609, 1436, 1279, 1179, 1109, 1021, 760, 739, 692. HRMS (DART) m/z calcd. for C_26_H_38_NO_3_S [M+NH_4_]^+^: 444.2567; found: 444.2561.

Colorless oil. ^1^H NMR (400 MHz, CDCl_3_): δ 7.94 (d, *J* = 8.2 Hz, 2H), 7.35-7.24 (m, 6H), 7.16 (t, *J* = 7.1 Hz, 1H), 3.90 (s, 3H), 2.92 (t, *J* = 7.3 Hz, 2H), 2.84 (dd, *J* = 14.8, 7.6 Hz, 1H), 2.67 (dd, *J* = 14.8, 8.4 Hz, 1H), 2.51 (t, *J* = 8.0 Hz, 1H), 1.96 (d, *J* = 12.3 Hz, 1H), 1.73 (d, *J* = 12.6 Hz, 1H), 1.69-1.53 (m, 4H), 1.49-1.32 (m, 4H), 1.01 (s, 6H). ^13^C NMR (101 MHz, CDCl_3_) δ 167.3, 147.0, 137.0, 129.8, 129.1, 129.0, 128.9, 127.9, 125.9, 73.5, 57.6, 52.1, 47.6, 36.9, 33.7, 32.9, 31.8, 29.4, 29.3, 29.0, 23.6, 22.2. IR (neat) cm^-1^ ṽ: 3497, 2947, 2859 1720, 1610, 1436, 1279, 1181, 1109, 1021, 759, 739, 692. HRMS (DART) m/z calcd. for C_26_H_38_NO_3_S [M+NH_4_]^+^: 444.2567; found: 444.2562.

**4-((2-hydroxy-2,4,4-trimethylcyclobutyl)methyl)benzonitrile**

**3l** was prepared according to general procedure in 66% isolated yield. dr = 1 : 2.0.

Colorless oil. ^1^H NMR (400 MHz, CDCl_3_): δ 7.55 (d, *J* = 8.2 Hz, 2H), 7.36 (d, *J* = 8.1 Hz, 2H), 2.94 (dd, *J* = 14.0, 9.5 Hz, 1H), 2.69 (dd, *J* = 13.9, 6.4 Hz, 1H), 2.14 (dd, *J* = 9.2, 6.8 Hz, 1H), 1.79 (dd, *J* = 36.6, 12.4 Hz, 2H), 1.18 (s, 3H), 1.11 (s, 3H), 1.03 (s, 3H). ^13^C NMR (101 MHz, CDCl_3_): δ 148.0, 132.2, 129.9, 119.3, 109.6, 72.4, 55.0, 49.2, 32.9, 31.5, 31.3, 31.0, 24.4. IR (neat) cm^-1^ ṽ: 3462, 2951, 2925, 2862, 1721, 1610, 1436, 1280, 1180, 1109, 1021, 754. HRMS (DART) m/z calcd. for C_15_H_20_NO [M+H]^+^: 230.1539; found: 230.1537.

Colorless oil. ^1^H NMR (400 MHz, CDCl_3_): δ 7.54 (d, *J* = 8.1 Hz, 2H), 7.37 (d, *J* = 8.1 Hz, 2H), 2.81 (dd, *J* = 15.0, 7.8 Hz, 1H), 2.67 (dd, *J* = 15.0, 7.9 Hz, 1H), 2.44 (t, *J* = 7.9 Hz, 1H), 1.89 (s, 2H), 1.32 (s, 3H), 1.02 (s, 6H). ^13^C NMR (101 MHz, CDCl_3_): δ 147.2, 132.2, 129.6, 119.2, 109.6, 71.2, 56.6, 50.3, 32.7, 32.4, 28.8, 24.9, 23.1. IR (neat) cm^-1^ ṽ: 3389, 2954, 2928, 2861, 1722, 1610, 1436, 1280, 1180, 1110, 1065, 1020, 799, 763. HRMS (DART) m/z calcd. for C_15_H_20_NO [M+H]^+^: 230.1539; found: 230.1538.

**1-(4-((2-hydroxy-2,4,4-trimethylcyclobutyl)methyl)phenyl)ethan-1-one**

**3m** was prepared according to general procedure in 50% isolated yield. dr = 1 : 1.8.

Colorless oil. ^1^H NMR (400 MHz, CDCl_3_): δ 7.87 (d, *J* = 8.1 Hz, 2H), 7.35 (d, *J* = 8.1 Hz, 2H), 2.93 (dd, *J* = 14.0, 9.4 Hz, 1H), 2.71 (dd, *J* = 14.0, 6.5 Hz, 1H), 2.58 (s, 3H), 2.20 (dd, *J* = 9.1, 6.8 Hz, 1H), 1.79 (dd, *J* = 29.9, 12.4 Hz, 1H), 1.19 (s, 3H), 1.11 (s, 3H), 1.04 (s, 3H). ^13^C NMR (101 MHz, CDCl_3_): δ 198.0, 148.0, 135.1, 129.3, 128.6, 72.5, 55.0, 49.1, 32.9, 31.4, 31.3, 31.0, 26.7, 24.5. IR (neat) cm^-1^ ṽ: 3470, 2952, 2922, 2863, 1675, 1605, 1413, 1360, 1269, 1226, 1183, 1110, 1017, 957, 846, 797, 597. HRMS (ESI) m/z calcd. for C_16_H_23_O_2_ [M+H]^+^: 247.1693; found: 247.1693.

Colorless oil. ^1^H NMR (400 MHz, CDCl_3_): δ 7.86 (d, *J* = 8.4 Hz,2H), 7.35 (d, *J* = 8.4 Hz, 2H), 2.81 (dd, *J* = 14.8, 7.8 Hz, 1H), 2.68 (dd, *J* = 14.8, 8.1 Hz, 1H), 2.57 (s, 3H), 2.48 (t, *J* = 8.0 Hz, 1H), 1.88 (d, *J* = 2.0 Hz, 2H), 1.34 (s, 3H), 1.03 (d, *J* = 4.9 Hz, 6H). ^13^C NMR (101 MHz, CDCl_3_): δ 198.1, 147.3, 135.1, 129.0, 128.6, 71.3, 56.9, 50.3, 32.8, 32.3, 28.9, 26.7, 25.0, 23.1. IR (neat) cm^-1^ ṽ: 3412, 2958, 2924, 2863, 1678, 1605, 1414, 1359, 1268, 1233, 1184, 1104, 1076, 1017, 956, 800, 596. HRMS (ESI) m/z calcd. for C_16_H_22_NaO_2_ [M+Na]^+^: 269.1512; found: 269.1512.

**N,N-diethyl-4-((2-hydroxy-2,4,4-trimethylcyclobutyl)methyl)benzamide**

**3n** was prepared according to general procedure in 49% isolated yield. dr = 1 : 2.5.

White solid. ^1^H NMR (400 MHz, CDCl_3_): δ 7.29-7.25 (m, 4H), 3.54 (s, 2H), 3.26 (s, 2H), 2.88 (dd, *J* = 14.1, 9.6 Hz, 1H), 2.68 (dd, *J* = 14.0, 6.4 Hz, 1H), 2.19 (dd, *J* = 9.5, 6.6 Hz, 1H), 1.78 (dd, *J* = 26.8, 12.3 Hz, 2H), 1.35-0.92 (m, 15H). ^13^C NMR (101 MHz, CDCl_3_): δ 171.6, 143.1, 134.7, 129.0, 126.6, 72.6, 54.9, 48.9, 43.5, 39.4, 32.7, 31.4, 31.1, 31.0, 24.5, 14.4, 13.0. IR (neat) cm^-1^ ṽ: 3422, 2953, 2865, 1614, 1459, 1430, 1380, 1288, 1261, 1225, 1097, 1020, 799. HRMS (ESI) m/z calcd. for C_19_H_30_NO_2_ [M+H]^+^: 304.2271; found: 304.2272.

Colorless oil. ^1^H NMR (400 MHz, CDCl_3_): δ 7.31-7.26 (m, 4H), 3.53 (s, 2H), 3.26 (s, 2H), 2.78 (dd, *J* = 14.8, 7.8 Hz, 1H), 2.64 (dd, *J* = 14.8, 8.1 Hz, 1H), 2.47 (t, *J* = 7.9 Hz, 1H), 1.93-1.84 (m, 2H), 1.38-0.95 (m, 15H). ^13^C NMR (101 MHz, CDCl_3_): δ 171.6, 142.5, 134.7, 128.8, 126.6, 71.3, 56.9, 50.2, 43.5, 39.4, 32.8, 32.0, 28.8, 24.9, 23.1, 14.3, 13.0. IR (neat) cm^-1^ ṽ: 3402, 2963, 2927, 2871, 1610, 1458, 1428, 1368, 1314, 1286, 1221, 1097, 956, 758, 7294, 574. HRMS (ESI) m/z calcd. for C_19_H_30_NO_2_ [M+H]^+^: 304.2271; found: 304.2272.

**N,N-diethyl-4-((2-hydroxy-2,4,4-trimethylcyclobutyl)methyl)benzenesulfonamide**

**3o** was prepared according to general procedure in 63% isolated yield. dr = 1 : 2.7.

White solid. ^1^H NMR (400 MHz, CDCl_3_): δ 7.69 (d, *J* = 8.3 Hz, 2H), 7.38 (d, *J* = 8.2 Hz, 2H), 3.22 (q, *J* = 7.1 Hz, 4H), 2.93 (dd, *J* = 13.9, 9.5 Hz, 1H), 2.70 (dd, *J* = 13.9, 6.4 Hz, 1H), 2.15 (dd, *J* = 9.3, 6.7 Hz, 1H), 1.82 (d, *J* = 12.5 Hz, 1H), 1.74 (d, *J* = 12.4 Hz, 1H), 1.18 (s, 3H), 1.13-1.06 (m, 9H), 1.02 (s, 3H). ^13^C NMR (101 MHz, CDCl_3_): δ 147.1, 137.7, 129.7, 127.2, 72.5, 55.1, 49.1, 42.0, 32.9, 31.3, 31.1, 31.0, 24.4, 14.1. IR (neat) cm^-1^ ṽ: 3519, 2952, 2916, 2896, 2867, 1596, 1464, 1374, 1348, 1324, 1311, 1201, 1182, 1148, 1090, 1013, 937, 789, 729, 699, 568, 544. HRMS (ESI) m/z calcd. for C_18_H_30_NO_3_S [M+H]^+^: 340.1941; found: 340.1940.

Colorless oil. ^1^H NMR (400 MHz, CDCl_3_): δ 7.68 (d, *J* = 8.3 Hz, 2H), 7.37 (d, *J* = 8.2 Hz, 2H), 3.21 (q, *J* = 7.1 Hz, 4H), 2.82 (dd, *J* = 14.9, 7.5 Hz, 1H), 2.66 (dd, *J* = 14.9, 8.3 Hz, 1H), 2.45 (t, *J* = 7.9 Hz, 1H), 1.94-1.72 (m, 2H), 1.32 (s, 3H), 1.09 (t, *J* = 7.1 Hz, 6H), 1.00 (d, *J* = 11.4 Hz, 6H). ^13^C NMR (101 MHz, CDCl_3_): δ 146.4, 137.7, 129.4, 127.1, 71.2, 56.8, 50.3, 42.0, 32.7, 32.1, 28.8, 24.9, 23.1, 14.2. IR (neat) cm^-1^ ṽ: 3504, 2958, 2870, 1464, 1382, 1330, 1199, 1151, 1090, 1015, 928, 785, 728, 704, 659, 571. HRMS (ESI) m/z calcd. for C_18_H_30_NO_3_S [M+H]^+^: 340.1941; found: 340.1942.

**methyl 4-((2-hydroxy-4-methyl-2-propylcyclobutyl)methyl)benzoate**

**3q** was prepared according to general procedure in 20% isolated yield.

Colorless oil. ^1^H NMR (400 MHz, CDCl_3_): δ 7.94 (d, *J* = 8.2 Hz, 2H), 7.28 (d, *J* = 8.2 Hz, 2H), 3.89 (s, 3H), 2.91 (dd, *J* = 14.0, 6.6 Hz, 1H), 2.61 (dd, *J* = 13.9, 9.0 Hz, 1H), 2.27 (dd, *J* = 11.1, 7.9 Hz, 1H), 2.10 (dd, *J* = 15.9, 9.1 Hz, 1H), 1.64 (s, 2H), 1.57-1.49 (m, 2H), 1.48-1.30 (m, 2H), 0.98 (t, *J* = 7.2 Hz, 2H), 0.77 (d, *J* = 6.5 Hz, 1H). ^13^C NMR (101 MHz, CDCl_3_): δ 167.3, 146.7, 129.8, 128.9, 127.9, 74.4, 57.5, 52.1, 41.5, 37.3, 35.5, 27.8, 21.2, 16.3, 14.9. IR (neat) cm^-1^ ṽ: 3484, 2956, 2929, 2870, 1722, 1610, 1436, 1279, 1179, 1109, 1020, 757. HRMS (ESI) m/z calc. for C_17_H_24_NaO_3_ [M+Na]^+^: 299.1618; found: 299.1615..

**methyl 4-((4-hydroxy-2,2-dimethylcyclobutyl)methyl)benzoate**

**3q** was prepared according to general procedure in 47% isolated yield. dr = 1 : 3.4.

Colorless oil. ^1^H NMR (400 MHz, CDCl_3_): δ 7.94 (d, *J* = 8.1 Hz, 2H), 7.32 (d, *J* = 8.1 Hz, 2H), 4.43 (dd, *J* = 11.1, 7.2 Hz, 1H), 3.90 (s, 3H), 3.02 (dd, *J* = 14.5, 10.0 Hz, 1H), 2.75 (dd, *J* = 14.6, 6.1 Hz, 1H), 2.58-2.40 (m, 1H), 2.12 (dd, *J* = 10.7, 6.7 Hz, 1H), 1.72 (dd, *J* = 12.2, 4.0 Hz, 1H), 1.58 (s, 3H), 1.13 (s, 3H), 1.09 (s, 3H). ^13^C NMR (101 MHz, CDCl_3_): δ 167.3, 147.7, 129.8, 128.9, 127.8, 66.3, 52.1, 50.1, 43.4, 34.3, 30.9, 30.8, 25.2. IR (neat) cm^-1^ ṽ: 3462, 2950, 2925, 2862, 1721, 1610, 1436, 1280, 1180, 1109, 1021, 754. HRMS (ESI) m/z calcd. for C_15_H_21_O_3_ [M+H]^+^: 249.1485; found: 249.1486.

Colorless oil. ^1^H NMR (400 MHz, CDCl_3_): δ 7.96 (d, *J* = 8.2 Hz, 2H), 7.31 (d, *J* = 8.1 Hz, 2H), 3.98 (dd, *J* = 15.5, 7.7 Hz, 1H), 3.90 (s, 3H), 2.87-2.67 (m, 2H), 2.16-2.06 (m, 2H), 1.60-1.57 (m, 1H), 1.04 (s, 3H), 1.00 (s, 3H). ^13^C NMR (101 MHz, CDCl_3_): δ 167.3, 146.6, 130.0, 128.8, 128.0, 69.4, 56.3, 52.1, 44.2, 35.4, 31.1, 30.4, 23.6. IR (neat) cm^-1^ ṽ: 3389, 2954, 2928, 2861, 1722, 1610, 1436, 1280, 1180, 1110, 1065, 1020, 799, 763. HRMS (ESI) m/z calcd. for C_15_H_20_NaO_3_ [M+Na]^+^: 271.1305; found: 271.1304.

**methyl 4-((2-hydroxyspiro[3.5]nonan-1-yl)methyl)benzoate**

**3r** was prepared according to general procedure in 56% isolated yield. dr = 1 : 2.4.

White solid. ^1^H NMR (400 MHz, CDCl_3_): δ 7.94 (d, *J* = 8.2 Hz, 2H), 7.33 (d, *J* = 8.2 Hz, 2H), 4.40 (td, *J* = 7.0, 4.9 Hz, 1H), 3.90 (s, 3H), 3.03 (dd, *J* = 14.6, 10.3 Hz, 1H), 2.80 (dd, *J* = 14.6, 5.7 Hz, 1H), 2.50-2.40 (m, 1H), 2.14 (ddd, *J* = 12.3, 7.0, 1.8 Hz, 1H), 1.76-1.69 (m, 2H), 1.52-1.10 (m, 9H). ^13^C NMR (101 MHz, CDCl_3_): δ 167.3, 148.0, 129.8, 128.9, 127.8, 66.1, 52.1, 50.1, 40.4, 39.9, 38.1, 34.2, 30.0, 26.2, 23.5, 22.7. IR (neat) cm^-1^ ṽ: 3355, 2923, 2849, 1715, 1607, 1435, 1272, 1187, 1106, 1081, 1059 1018, 960, 757, 744, 695, 483. HRMS (ESI) m/z calcd. for C_18_H_25_O_3_ [M+H]^+^: 289.1798; found: 289.1798.

White solid. ^1^H NMR (400 MHz, CDCl_3_): δ 7.95 (d, *J* = 8.2 Hz, 2H), 7.31 (d, *J* = 8.1 Hz, 2H), 3.96 (q, *J* = 7.7 Hz, 1H), 3.89 (s, 3H), 2.87-2.72 (m, 2H), 2.30 (dd, *J* = 11.2, 7.7 Hz, 1H), 2.05 (q, *J* = 7.8 Hz, 1H), 1.71-1.00 (m, 11H). ^13^C NMR (101 MHz, CDCl_3_): δ 167.3, 146.9, 129.9, 128.9, 127.9, 69.5, 56.9, 52.1, 41.1, 40.5, 35.1, 34.7, 32.4, 26.2, 23.3, 22.8. IR (neat) cm^-1^ ṽ: 3411, 2923, 2850, 1720, 1610, 1436, 1415, 1277, 1180, 1108, 1048, 1020, 762. HRMS (ESI) m/z calcd. for C_18_H_24_NaO_3_ [M+Na]^+^: 311.1618; found: 311.1618.

**Methyl 4-((2-((diisopropyl(methyl)silyl)oxy)-2-methylspiro[3.5]nonan-1-yl)meth-yl)ben**

**-zoate**

**3s** was prepared according to general procedure in 41% isolated yield.

Colorless oil. ^1^H NMR (400 MHz, CDCl_3_): δ 7.92 (d, *J* = 8.2 Hz, 2H), 7.39 (d, *J* = 8.2 Hz, 2H), 3.90 (s, 3H), 2.82 (dd, *J* = 15.4, 8.4 Hz, 1H), 2.72 (dd, *J* = 15.5, 7.5 Hz, 1H), 2.47 (t, *J* = 8.0 Hz, 1H), 1.95 (d, *J* = 12.0 Hz, 1H), 1.86 (d, *J* = 11.7 Hz, 1H), 1.63 (d, *J* = 7.7 Hz, 1H), 1.60-1.49 (m, 2H), 1.47-1.40 (m, 2H), 1.29-1.20 (m, 2H), 1.02-0.92(m, 12H), 0.90-0.79 (m, 2H), 0.30 (s, 3H). ^13^C NMR (101 MHz, CDCl_3_): δ 167.5, 147.735, 129.6, 129.0, 127.5, 72.7, 57.7, 52.1, 47.5, 42.3, 33.7, 31.8, 31.4, 26.8, 26.2, 23.2, 22.58, 17.9, 17.9, 17.7, 14.2, 14.1, -5.2. IR (neat) cm^-1^ ṽ: 2925, 2861, 1726, 1278, 1260, 1097, 1023, 799. HRMS (DART) m/z calcd. for C_26_H_43_O_3_Si [M+H]^+^: 431.2976; found: 431.2971.

**methyl 4-((2-hydroxyspiro[3.4]octan-1-yl)methyl)benzoate**

**3t** was prepared according to general procedure in 53% isolated yield. dr = 1 : 2.9.

Yellow oil. ^1^H NMR (400 MHz, CDCl_3_): δ 7.94 (d, *J* = 8.2 Hz, 2H), 7.32 (d, *J* = 8.2 Hz, 2H), 4.43 (dd, *J* = 12.4, 6.8 Hz, 1H), 3.90 (s, 3H), 3.07 (dd, *J* = 14.6, 9.6 Hz, 1H), 2.76 (dd, *J* = 14.6, 6.3 Hz, 1H), 2.70-2.61 (m, 1H), 2.18 (ddd, *J* = 11.8, 6.8, 2.2 Hz, 1H), 1.84 (dd, *J* = 11.7, 5.3 Hz, 1H), 1.76-1.41 (m, 8H). ^13^C NMR (101 MHz, CDCl_3_): δ 167.3, 147.8, 129.8, 128.9, 127.8, 66.1, 52.1, 49.1, 44.6, 42.8, 40.6, 34.7, 31.5, 23.7, 23.6. IR (neat) cm^-1^ ṽ: 3432, 2949, 2924, 2855, 1720, 1609, 1435, 1277, 1179, 1109, 1056, 1019, 756, 732, 701. HRMS (ESI) m/z calcd. for C_17_H_23_O_3_ [M+H]^+^: 275.1641; found: 275.1642.

Yellow oil. ^1^H NMR (400 MHz, CDCl_3_): δ 7.95 (d, *J* = 8.1 Hz, 2H), 7.31 (d, *J* = 8.1 Hz, 2H), 4.00-3.83 (m, 4H), 2.86-2.69 (m, 2H), 2.35 (dd, *J* = 15.5, 7.8 Hz, 1H), 2.17 (dd, *J* = 10.7, 7.5 Hz, 1H), 1.69-1.35 (m, 9H). ^13^C NMR (101 MHz, CDCl_3_): δ 167.2, 146.6, 123.0, 128.8, 128.0, 69.5, 54.3, 52.1, 44.4, 41.3, 40.3, 36.0, 33.1, 24.4, 23.4. IR (neat) cm^-1^ ṽ: 3414, 2951, 2855, 1721, 1610, 1436, 1278, 1180, 1107, 1054, 1019, 798, 762. HRMS (ESI) m/z calcd. for C_17_H_22_NaO_3_ [M+Na]^+^: 297.1462; found: 297.1461.

**methyl 4-((2-hydroxyspiro[3.6]decan-1-yl)methyl)benzoate**

**3u** was prepared according to general procedure in 49% isolated yield. dr = 1 : 3.6.

White solid. ^1^H NMR (400 MHz, CDCl_3_): δ 7.94 (d, *J* = 8.3 Hz, 2H), 7.33 (d, *J* = 8.3 Hz, 2H), 4.40 (dd, *J* = 11.7, 6.9 Hz, 1H), 3.90 (s, 3H), 3.03 (dd, *J* = 14.6, 10.1 Hz, 1H), 2.79 (dd, *J* = 14.6, 6.0 Hz, 1H), 2.56-2.44 (m, 1H), 2.14 (ddd, *J* = 12.2, 7.0, 1.9 Hz, 1H), 1.81-1.61 (m, 4H), 1.53-1.22 (m, 9H). ^13^C NMR (101 MHz, CDCl_3_): δ 167.3, 147.9, 129.8, 128.9, 127.7, 65.9, 52.1, 51.4, 43.4, 41.6, 40.9, 36.7, 30.7, 28.6, 28.4, 23.3, 22.8. IR (neat) cm^-1^ ṽ: 3465, 2921, 2853, 1722, 1610, 1436, 1279, 1180, 1109, 1020, 800. HRMS (ESI) m/z calcd. for C_19_H_27_O_3_ [M+H]^+^: 303.1955; found: 303.1954.

Colorless oil. ^1^H NMR (400 MHz, CDCl_3_): δ 7.95 (d, *J* = 7.6 Hz, 2H), 7.31 (d, *J* = 7.8 Hz, 2H), 3.99-3.84 (m, 4H), 2.87-2.73 (m, 2H), 2.23 (dd, *J* = 11.0, 7.7 Hz, 1H), 2.11 (q, *J* = 7.8 Hz, 1H), 1.70-1.30 (m, 13H). ^13^C NMR (101 MHz, CDCl_3_): δ 167.3, 146.9, 130.0, 128.9, 128.0, 69.2, 58.7, 52.1, 44.3, 41.9, 37.7, 35.5, 35.2, 28.7, 28.3, 23.4, 23.0. IR (neat) cm^-1^ ṽ: 3398, 2921, 2852, 1720, 1609, 1435, 1276, 1179, 1107, 1048, 1020, 760, 702. HRMS (ESI) m/z calcd. for C_19_H_26_NaO_3_ [M+Na]^+^: 325.1774; found: 325.1775.

**methyl 4-((2-hydroxy-4-(4-hydroxy-4-methylpentyl)-2,4-dimethylcyclobutyl)met-hyl)ben-zoate**

**3v** was prepared according to general procedure in 38% isolated yield. dr = 1 : 2.2.

Colorless oil. ^1^H NMR (400 MHz, CDCl_3_): δ 7.94 (d, *J* = 8.1 Hz, 2H), 7.32 (d, *J* = 7.9 Hz, 2H), 3.90 (s, 3H), 2.94 (dd, *J* = 13.4, 9.9 Hz, 1H), 2.70 (dd, *J* = 13.8, 6.4 Hz, 1H), 2.20-2.13 (m, 1H), 1.81 (d, *J* = 12.3 Hz, 1H), 1.70 (d, *J* = 12.5 Hz, 1H), 1.62 (s, 1H), 1.42-1.34 (m, 2H), 1.33-1.22 (m, 4H), 1.21-1.15 (m, 9H), 1.09 (s, 3H). ^13^C NMR (101 MHz, CDCl_3_): δ 167.3, 147.7, 129.8, 129.2, 127.8, 72.6, 71.1, 54.7, 52.1, 48.0, 45.5, 44.6, 36.2, 31.7, 31.1, 29.4, 29.4, 21.4, 19.5. IR (neat) cm^-1^ ṽ: 3426, 2955, 1706, 1609, 1436, 1374, 1277, 1179, 1110, 1020, 907, 758, 733, 703. HRMS (ESI) m/z calcd. for C_21_H_32_NaO_4_ [M+Na]^+^: 371.2193; found: 371.2191

Colorless oil. ^1^H NMR (400 MHz, CDCl_3_): δ 7.93 (d, *J* = 8.0 Hz, 2H), 7.32 (d, *J* = 7.9 Hz, 2H), 3.89 (s, 3H), 2.82 (dd, *J* = 14.5, 7.4 Hz, 1H), 2.68 (dd, *J* = 14.6, 8.0 Hz, 1H), 2.45 (t, *J* = 7.8 Hz, 1H), 1.88 (d, *J* = 12.0 Hz, 1H), 1.82 (d, *J* = 12.3 Hz, 1H), 1.75 (s, 1H), 1.40-1.17 (m, 9H), 1.11 (s, 6H), 1.04 (s, 3H). ^13^C NMR (101 MHz, CDCl_3_): δ 167.1, 147.0, 129.7, 128.9, 127.7, 71.2, 70.9, 56.6, 52.0, 49.1, 46.5, 44.3, 32.5, 32.0, 29.2, 29.1, 25.1, 20.2, 19.0. IR (neat) cm^-1^ ṽ: 3376, 2962, 2933, 2845, 1706, 1609, 1435, 1378, 1278, 1180, 1110, 1020, 907, 758, 730. HRMS (ESI) m/z calcd. for C_21_H_32_NaO_4_ [M+Na]^+^: 371.2193; found: 371.2190.

**Methyl 4-((2-hydroxy-2,4-dimethyl-4-(4-methylpentyl)cyclobutyl)methyl)ben**

**-zoate**

**3w** was prepared according to general procedure in 64% isolated yield. dr = 1 : 2.3.

Yellow oil. ^1^H NMR (400 MHz, CDCl_3_): δ 7.94 (d, *J* = 8.2 Hz, 2H), 7.32 (d, *J* = 8.1 Hz, 2H), 3.90 (s, 3H), 2.95 (dd, *J* = 14.1, 9.9 Hz, 1H), 2.69 (dd, *J* = 13.8, 6.0 Hz, 1H), 2.16 (dd, *J* = 9.6, 6.1 Hz, 1H), 1.80 (d, *J* = 12.4 Hz, 1H), 1.68 (d, *J* = 12.4 Hz, 1H), 1.54-1.44 (m, 1H), 1.32-1.23 (m, 4H), 1.17 (s, 3H), 1.12-1.09 (m, 2H), 1.07 (s, 3H), 0.84 (dd, *J* = 6.6, 1.8 Hz, 6H). ^13^C NMR (101 MHz, CDCl_3_): δ 167.3, 147.8, 129.8, 129.2, 127.8, 72.6, 54.6, 52.1, 48.1, 45.2, 39.8, 36.1, 31.8, 31.1, 28.1, 22.8, 22.8, 22.5, 21.5. IR (neat) cm^-1^ ṽ: 3502, 2953, 2924, 2868, 1723, 1609, 1436, 1277, 1178, 1107, 1019, 797, 759. HRMS (ESI) m/z calcd. for C_21_H_33_O_3_ [M+H]^+^: 333.2424; found: 333.2422.

Yellow oil. ^1^H NMR (400 MHz, CDCl_3_): δ 7.94 (d, *J* = 8.3 Hz, 2H), 7.32 (d, *J* = 8.3 Hz, 2H), 3.89 (s, 3H), 2.81 (dd, *J* = 14.8, 8.1 Hz, 1H), 2.68 (dd, *J* = 14.7, 7.6 Hz, 1H), 2.45 (t, *J* = 7.8 Hz, 1H), 1.88 (d, *J* = 12.2 Hz, 1H), 1.81 (d, *J* = 12.1 Hz, 1H), 1.49-1.39 (m, 1H), 1.34 (s, 1H), 1.30-1.22 (m, 4H), 1.05-1.00 (m, 5H), 0.80 (dd, *J* = 6.6, 3.7 1Hz, 6H). ^13^C NMR (101 MHz, CDCl_3_): δ 167.3, 147.2, 129.8, 128.9, 127.8, 71.5, 56.6, 52.1, 49.2, 46.5, 39.6, 32.7, 32.1, 28.0, 25.3, 22.7, 22.7, 22.2, 20.4. IR (neat) cm^-1^ ṽ: 3432, 2953, 2925, 2869, 1722, 1610, 1435, 1277, 1180, 1109, 1019, 758, 735, 702. HRMS (ESI) m/z calcd. for C_21_H_32_NaO_3_ [M+Na]^+^: 355.2244; found: 355.2244.

**Methyl 4-(((1S,4R,6R,7R)-6-hydroxy-4,8,8-trimethylbicyclo[4.2.0]octan-7-yl)**

**methyl)benzoate**

**3x** was prepared according to general procedure in 30% isolated yield. dr = 2.0 : 1.

White solid. ^1^H NMR (400 MHz, CDCl_3_): δ 7.93 (d, *J* = 8.2 Hz, 2H), 7.30 (d, *J* = 8.1 Hz, 2H), 3.90 (s, 3H), 2.84 (dd, *J* = 13.7, 8.3 Hz, 1H), 2.68 (dd, *J* = 13.8, 7.2 Hz, 1H), 2.25 (t, *J* = 7.8 Hz, 1H), 1.84-1.73 (m, 1H), 1.73-1.61 (m, 1H), 1.56-1.49 (m, 2H), 1.48 -1.21 (m, 7H), 0.84 (s, 3H), 0.81 (d, *J* = 6.6 Hz, 3H). ^13^C NMR (101 MHz, CDCl_3_): δ 167.4, 147.8, 129.7, 129.1, 127.7, 75.4, 52.1, 50.3, 49.4, 46.2, 36.9, 31.8, 30.5, 30.0, 25.8, 25.4, 24.8, 22.8. IR (neat) cm^-1^ ṽ: 3509, 2947, 2867, 1707, 1609 1436, 1277, 1178, 1108, 1022, 760. HRMS (ESI) m/z calcd. for C_20_H_28_NaO_3_ [M+Na]^+^: 339.1931; found: 339.1930.

White solid. ^1^H NMR (400 MHz, CDCl_3_): δ 7.94 (d, *J* = 8.2 Hz,2H), 7.32 (d, *J* = 8.2 Hz, 2H), 3.89 (s, 3H), 2.77 (dd, *J* = 14.5, 8.0 Hz, 1H), 2.65 (dd, *J* = 14.5, 7.8 Hz, 1H), 2.31 (t, *J* = 7.9 Hz, 1H), 2.09-1.92 (m, 2H), 1.69-1.55 (m, 2H), 1.38-1.20 (m, 4H), 1.03 (d, *J* = 7.0 Hz, 3H), 1.00 (s, 3H), 0.97 (s, 3H). ^13^C NMR (101 MHz, CDCl_3_): δ 167.3, 147.3, 129.8, 129.0, 127.8, 72.5, 59.1, 52.1, 51.0, 35.7, 33.5, 32.9, 31.5, 27.9, 26.0, 21.2, 18.7, 16.6. IR (neat) cm^-1^ ṽ: 3505, 2944, 2919, 2856, 1700, 1607, 1436, 1313, 1280, 1199, 1178, 1107, 1026, 799, 757, 732, 700, 489. HRMS (ESI) m/z calcd. for C_20_H_28_NaO_3_ [M+Na]^+^: 339.1931; found: 339.1928.

**methyl 4-((5-hydroxy-7,7-dimethylbicyclo[3.2.0]heptan-6-yl)methyl)benzoate**

**3y** was prepared according to general procedure in 60% isolated yield. dr = 1.3 : 1.

White solid. ^1^H NMR (400 MHz, CDCl_3_): δ 7.93 (d, *J* = 8.3 Hz, 2H), 7.32 (d, *J* = 8.1 Hz, 2H), 3.89 (s, 3H), 2.98 (dd, *J* = 14.1, 9.6 Hz, 1H), 2.72 (dd, *J* = 14.1, 6.2 Hz, 1H), 2.06 (dd, *J* = 9.5, 6.3 Hz, 1H), 1.85 (d, *J* = 8.1 Hz, 1H), 1.82-1.74 (m, 1H), 1.73-1.54 (m, 3H), 1.46-1.39 (m, 1H), 1.37-1.23 (m, 1H), 1.17 (s, 3H), 0.83 (s, 3H). ^13^C NMR (101 MHz, CDCl_3_): δ 167.3, 148.0, 129.8, 129.0, 127.7, 83.2, 53.8, 52.1, 51.4, 40.3, 34.0, 31.1, 26.3, 26.3, 26.2, 23.7. IR (neat) cm^-1^ ṽ: 3463, 2944, 2922, 2892, 2854, 1695, 1608, 1434, 1299, 1223, 1148, 1121, 1067, 756, 702, 513. HRMS (ESI) m/z calcd. for C_18_H_24_NaO_3_ [M+Na]^+^: 311.1618; found: 311.1616.

White solid. ^1^H NMR (400 MHz, CDCl_3_): δ 7.94 (d, *J* = 8.3 Hz, 2H), 7.32 (d, *J* = 8.4 Hz, 2H), 3.89 (s, 3H), 2.72 (ddd, *J* = 22.1, 14.8, 8.1 Hz, 2H), 2.45 (t, *J* = 8.1 Hz, 1H), 2.13-2.07 (m, 1H), 1.94-1.86 (m, 1H), 1.85-1.69 (m, 3H), 1.63-1.54 (m, 1H), 1.50 (dd, *J* = 12.9, 6.8 Hz, 1H), 1.15 (s, 3H), 0.81 (s, 3H). ^13^C NMR (101 MHz, CDCl_3_): δ 167.3, 147.2, 129.8, 128.8, 127.8, 83.0, 56.3, 55.3, 52.1, 36.3, 34.5, 32.3, 31.3, 27.3, 26.1, 18.5. IR (neat) cm^-1^ ṽ: 3432, 2950, 2861, 1720, 1610, 1435, 1277, 1180, 1109, 1067, 1020, 757. HRMS (ESI) m/z calcd. for C_18_H_24_NaO_3_ [M+Na]^+^: 311.1618; found: 311.1618.

**Methyl 4-((1-hydroxyspiro[bicyclo[4.2.0]octane-7,1'-cyclohexan]-8-yl)methyl)**

**benzoate**

**3z** was prepared according to general procedure in 59% isolated yield. dr = 1 : 2.6.

Colorless oil. ^1^H NMR (400 MHz, CDCl_3_): δ 7.93 (d, *J* = 8.2 Hz, 2H), 7.32 (d, *J* = 8.2 Hz, 2H), 3.90 (s, 3H), 2.94 (dd, *J* = 14.0, 10.1 Hz, 1H), 2.75 (dd, *J* = 14.0, 5.8 Hz, 1H), 2.21 (dd, *J* = 10.1, 5.7 Hz, 1H), 1.99 (d, *J* = 11.0 Hz, 1H), 1.88-1.81 (m, 1H), 1.76-1.05 (m, 18H). ^13^C NMR (101 MHz, CDCl_3_): δ 167.4, 148.3, 129. 8, 129.0, 127.7, 73.6, 52.6, 52.1, 48.7, 40.5, 37.2, 35.0, 34.7, 30.4, 26.3, 24.4, 23.0, 22.2, 21.5, 20.2. IR (neat) cm^-1^ ṽ: 3511, 2923, 2852, 1721, 1609, 1436, 1278, 1179, 1108, 1018.73, 802.64, 754.46. HRMS (EI, 70eV) m/z calcd. for C_22_H_26_O_3_ [M]^+^: 338.1876; found: 338.1884.

Colorless oil. ^1^H NMR (400 MHz, CDCl_3_): δ 7.94 (d, *J* = 8.2 Hz, 2H), 7.34 (d, *J* = 8.1 Hz, 2H), 3.89 (s, 3H), 2.79 (d, *J* = 8.3 Hz, 1H), 2.28 (t, *J* = 8.1 Hz, 1H), 1.95-1.89 (m, 1H), 1.88-1.78 (m, 2H), 1.73-1.20 (m, 16H). ^13^C NMR (101 MHz, CDCl_3_): δ 167.3, 147.5, 129.8, 129.0, 127.8, 72.3, 58.5, 52.1, 51.8, 44.9, 37.6, 32.2, 30.7, 29.1, 26.2, 23.9, 23.8, 22.1, 21.5, 19.4. IR (neat) cm^-1^ ṽ: 3495, 2924, 2852, 1696, 1607, 1427, 1284, 1262, 1180, 1105, 1006, 801, 755, 730, 698, 547. HRMS (ESI) m/z calcd. for C_21_H_30_NaO_3_ [M+Na]^+^: 365.2087; found: 365.2088.

**methyl 4-((6-butyl-8-hydroxybicyclo[4.2.0]octan-7-yl)methyl)benzoate**

**3aa** was prepared according to general procedure in 35% isolated yield.

Colorless oil. ^1^H NMR (400 MHz, CDCl_3_): δ 7.94 (d, *J* = 8.2 Hz, 2H), 7.30 (d, *J* = 8.1 Hz, 2H), 3.90 (s, 3H), 3.82 (t, *J* = 8.1 Hz, 1H), 2.92-2.71 (m, 2H), 1.90 (dd, *J* = 15.4, 7.7 Hz, 1H), 1.72-1.02 (m, 16H), 0.83 (t, *J* = 7.0 Hz, 3H). ^13^C NMR (101 MHz, CDCl_3_): δ 167.3, 147.2, 129.9, 128.9, 127.9, 71.4, 53.5, 52.2, 47.4, 40.3, 35.4, 29.6, 27.2, 23.5, 23.1, 22.8, 21.9, 14.3. IR (neat) cm^-1^ ṽ: 3429, 2925, 2856, 1722, 1609, 1436, 1277.83, 1180, 1110, 1021, 764. HRMS (ESI) m/z calcd. for C_21_H_30_NaO_3_ [M+Na]^+^: 353.2807; found: 353.2807.

- 1. Synthetic applications

1.4.1 Synthesis of γ-fluorinated ketone 4^4^

**Experimental procedure**：In a dried sealed vial, cyclopropanol **3a** (0.076 mmol, 1.0 equiv), AgBF_4_ (0.025 mmol, 0.25 equiv), and SelectFluor (0.229 mmol, 3.0 equiv) were dissolved in DCE/H_2_O (1mL/1mL) under a N_2_ atmosphere, and the mixture was stirred for 32 h at room temperature. The mixture was extracted with CH_2_Cl_2_ (3× 10 mL). The combined organic extracts were washed by brine, dried over Na_2_SO_4_, filtered, concentrated, and purified by flash chromatography on silica gel.

**methyl 4-(2-fluoro-3,3-dimethyl-5-oxohexyl)benzoate** 48% yield, Colorless oil. ^1^H NMR (400 MHz, CDCl_3_): δ 7.98 (d, *J* = 8.1 Hz, 1H), 7.31 (d, *J* = 8.1 Hz, 1H), 4.65 (ddd, *J* = 47.7, 8.4, 3.6 Hz, 1H), 3.91 (s, 3H), 2.97-2.86 (m, 1H), 2.86-2.81 (m, 1H), 2.66 (d, *J* = 16.0 Hz, 1H), 2.41 (d, *J* = 16.0 Hz, 1H), 2.15 (s, 3H), 1.13 (d, *J* = 11.3 Hz, 6H). ^19^F NMR (376 MHz, CDCl_3_): δ -188.2--188.6 (m, 1F). ^13^C NMR (101 MHz, CDCl_3_): δ 208.1, 167.2, 144.0, 129.9, 129.4, 128.6, 99.1 (d, J = 177.0 Hz), 52.2, 50.8 (d, J = 4.8 Hz), 37.8 (d, J = 18.7 Hz), 36.2 (d, J = 22.1 Hz), 32.4, 23.4 (d, J = 5.6 Hz), 22.7 (d, J = 3.2 Hz). IR (neat) cm^-1^ ṽ: 2964, 1719, 1612, 1435, 1365, 1280, 1181, 1107.77, 1067, 1020, 800, 755. HRMS (EI, 70eV) m/z calcd. for C_16_H_21_FO_3_ [M]^+^: 280.1469; found: 280.1474.

1.4.2 Synthesis of γ-Arylation ketone 5^5^

**Experimental procedure**：A dried screw-cap test tube containing a stirring bar was charged with the cyclobutanol **3a** (0.076 mmol, 1.0 equiv), tricyclohexylphosphane (0.0038 mmol, 5 mol %), NaO*t*Bu (0.084 mmol, 1.10 equiv.), chlorobenzene(0.099 mmol, 1.3 equiv.), toluene (2 mL) and Pd(OAc)_2_ (0.0019, 2.5 mol %) were then added by syringe. The mixture was then stirred in a pre-heated oil bath (110℃) for 24 h. The mixture was then allowed to warm to room temperature and diluted with EtOAc (5 mL) and filtered through a Celite plug, eluting with additional EtOAc (10 mL). The filtrate was concentrated and purified by column chromatography on silica gel.

**methyl 4-(2-acetyl-3,3-dimethyl-4-phenylbutyl)benzoate** 65% yield, white solid. ^1^H NMR (400 MHz, CDCl_3_): δ 7.94 (d, *J* = 8.1 Hz, 1H), 7.35-7.28 (m, 2H), 7.28-7.14 (m, 5H), 3.90 (s, 3H), 3.07-2.93 (m, 1H), 2.84 (dd, *J* = 11.1, 3.4 Hz, 1H), 2.76 (d, *J* = 13.0 Hz, 1H), 2.67 (d, *J* = 13.0 Hz, 1H), 1.75 (s, 3H), 0.99 (d, *J* = 14.1 Hz, 6H). ^13^C NMR (101 MHz, CDCl_3_): δ 213.1, 167.1, 146.0, 138.1, 130.9, 130.0, 129.1, 128.4, 128.1, 126.5, 62.9, 52.2, 46.3, 37.7, 35.6, 34.5, 25.3, 24.4. IR (neat) cm^-1^ ṽ: 2961, 2943, 2925, 2871, 2852, 1714, 1704, 1607, 1432, 1356, 1276, 1179, 1104, 1022, 967, 860, 775, 759, 722, 700, 521, 496. HRMS (EI, 70eV) m/z calcd. for C_22_H_26_O_3_ [M]^+^: 338.1876; found: 338.1884.

- 1. Mechanistic investigations

1.5.1 Deuterium-labeling Experiments

**Experimental procedure**：In a dried sealed vial, [(DPEphos)(bcp)Cu]PF_6_ (10 mol %) (0.010 mmol, 10 mol %), DIPEA (0.4 mmol, 4.0 equiv), and terminal alkyne (0.15 mmol, 1.5 equiv) were dissolved in CD_3_CN (1.0 mL) under N_2_ atmosphere. Then, tethered alcohol (0.1 mmol, 1.0 equiv) was added. The reaction mixture was stirred at room temperature under B-LED for 24 h. The distance between the vial and the lamp was about 1–3 cm. The resulting mixture was filtered and concentrated. After that, the residual oil was dissolved in THF (1.0 mL), followed by the addition of TBAF (2 mL, 1.0 M in THF). The reaction mixture was stirring at room temperature until the reaction was completed, as monitored by TLC analysis. Then the mixture was diluted with EtOAc, washed with saturated NaCl (aq.) and dried over with anhydrous Na_2_SO_4_. After filtration and concentration, the residue was purified by silica gel chromatography with petroleum ether and ethyl acetate afford the deprotected products.

**Experimental procedure**：In a dried sealed vial, [(DPEphos)(bcp)Cu]PF_6_ (10 mol %) (0.010 mmol, 10 mol %), DIPEA (0.4 mmol, 4.0 equiv) and terminal alkyne (0.15 mmol, 1.5 equiv) were dissolved in CH_3_CN (1.0 mL) under N_2_ atmosphere. Then deuterated tethered alcohol **1c-*d1*** (0.1 mmol, 1.0 equiv) was added. The reaction mixture was stirred at room temperature under B-LED for 24 h. The distance between the vial and the lamp was about 1–3 cm. The resulting mixture was filtered and concentrated. After that, the residual oil was dissolved in THF (1.0 mL), followed by the addition of TBAF (2 mL, 1.0 M in THF). The reaction mixture was stirring at room temperature until the reaction was completed, as monitored by TLC analysis. Then the mixture was diluted with EtOAc, washed with saturated NaCl (aq.) and dried over with anhydrous Na_2_SO_4_. After filtration and concentration, the residue was purified by silica gel chromatography with petroleum ether and ethyl acetate afford the deprotected products.


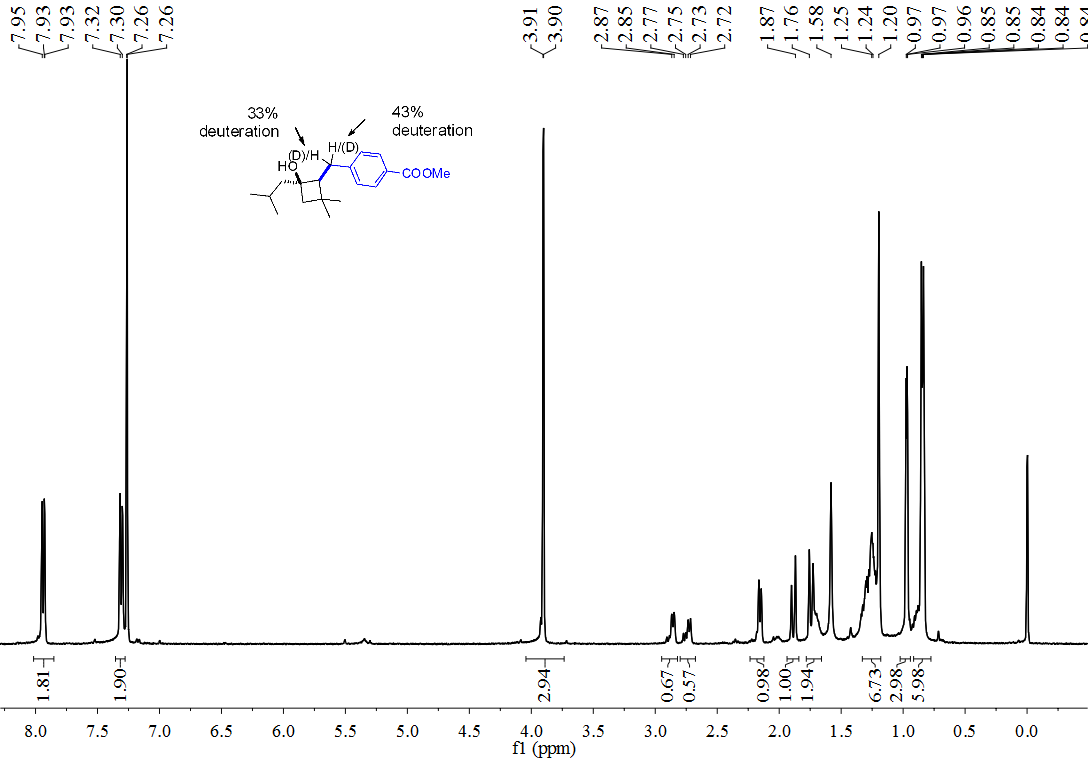


**Supplementary Fig. 1.** ^1^H NMR spectra of compound **3c_1_-*d2***.


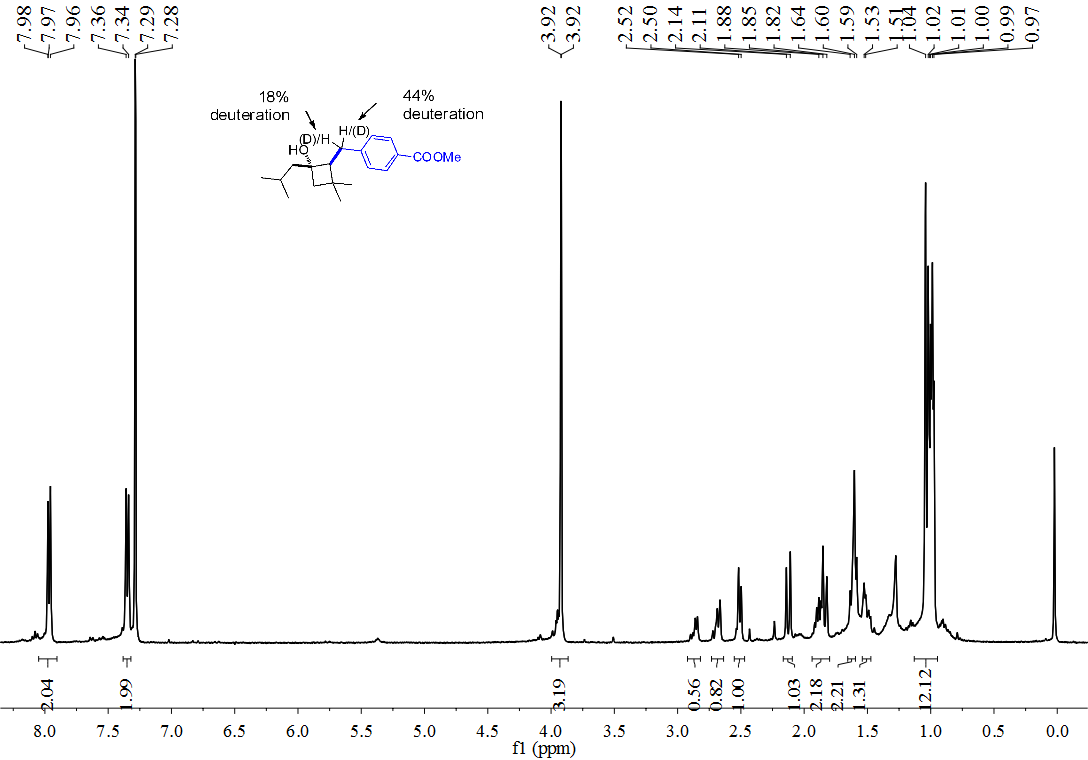


**Supplementary Fig. 2.** ^1^H NMR spectra of compound **3c_2_-*d2***.

**Experimental procedure**：In a dried sealed vial, [(DPEphos)(bcp)Cu]PF_6_ (10 mol %) D_2_O (0.10 mmol, 1.0 equiv), DIPEA (0.4 mmol, 4.0 equiv) and terminal alkyne (0.15 mmol, 1.5 equiv) were dissolved in CH_3_CN (1.0 mL) under N_2_ atmosphere. Then, tethered alcohol (0.1 mmol, 1.0 equiv) was added. The reaction mixture was stirred at room temperature under B-LED for 24 h. The distance between the vial and the lamp was about 1–3 cm. The resulting mixture was filtered and concentrated. After that, the residual oil was dissolved in THF (1.0 mL), followed by the addition of TBAF (2 mL, 1.0 M in THF). The reaction mixture was stirring at room temperature until the reaction was completed, as monitored by TLC analysis. Then the mixture was diluted with EtOAc, washed with saturated NaCl (aq.) and dried over with anhydrous Na_2_SO_4_. After filtration and concentration, the residue was purified by silica gel chromatography with petroleum ether and ethyl acetate afford the deprotected products.


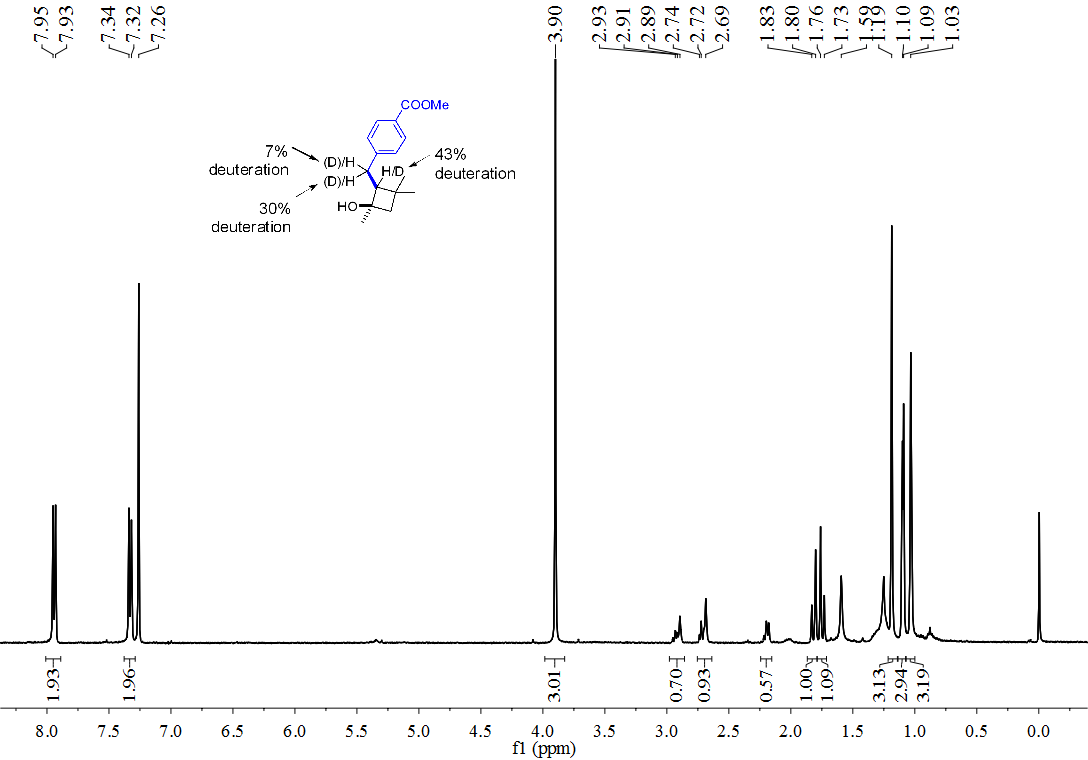


**Supplementary Fig. 3.** ^1^H NMR spectra of compound **3a_1_-*d3***.


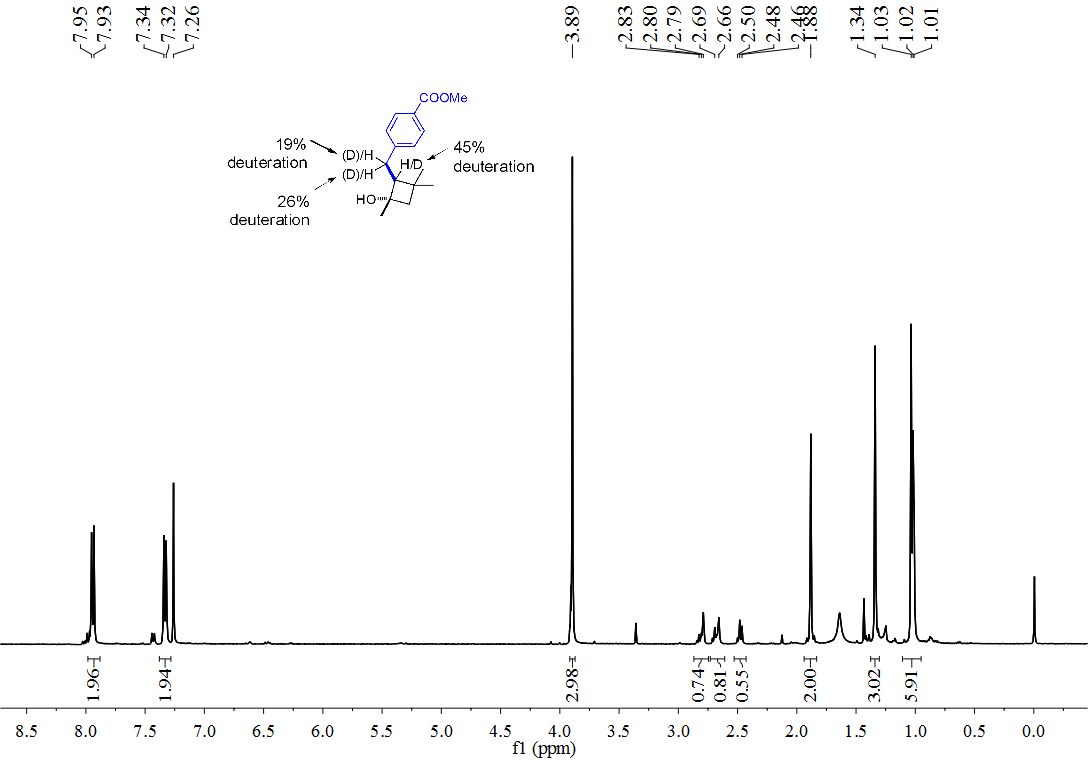


**Supplementary Fig. 4.** ^1^H NMR spectra of compound **3a_1_-*d3***.

1.5.2 Radical scavenger study

The radical scavenger experiments showed that TEMPO greatly hampered the reaction. We did not fund the adducts of TEMPO with initially formed silyl methyl radical or radicals generated after HAT. The excited-state [(DPEphos)(bcp)Cu]PF_6_ can be quenched by the TEMPO according to Stern-Volmer experiment.

1.5.3 EPR study

General Procedure for the ERP Analysis. After 30 min, the solution sample was taken out into a small tube and then analyzed by EPR. EPR spectra was recorded at room temperature on a EPR spectrometer operated at 9.371 GHz. Typical spectrometer parameters are shown as follows, sweep width: 50 G; center field: 3480 G; static field: 3480G; conversion time: 5.12 ms; sweep time: 5.24 s; modulation amplitude: 1.0 G; modulation frequency: 100 kHz; receiver gain: 2 × 10^4^.

The EPR spectrum of control experiment showed the existence of carbon radical (g = 2.005)


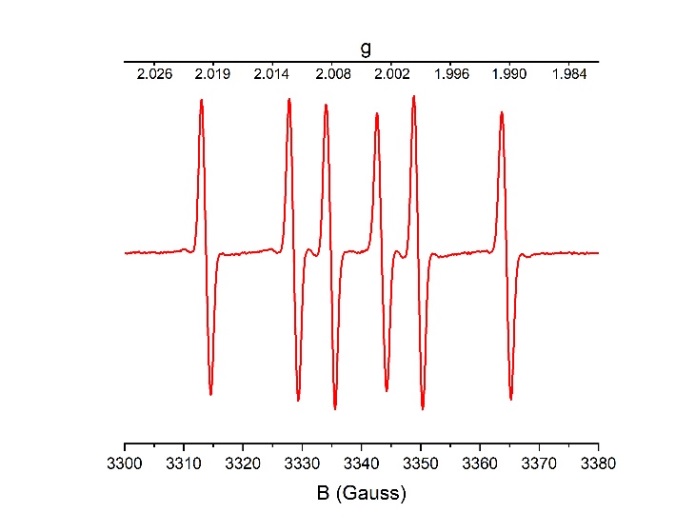


**Supplementary Fig. 5.** EPR spectra of reaction in the presence of DMPO.

1.5.4 Photophysical studies

**UV-visible Spectroscopic Study of [(DPEphos)(bcp)Cu]PF_6_**

**Experimental details**

The UV-Vis spectra of [(DPEphos)(bcp)Cu]PF_6_ was measured directly in MeCN.^6^

**
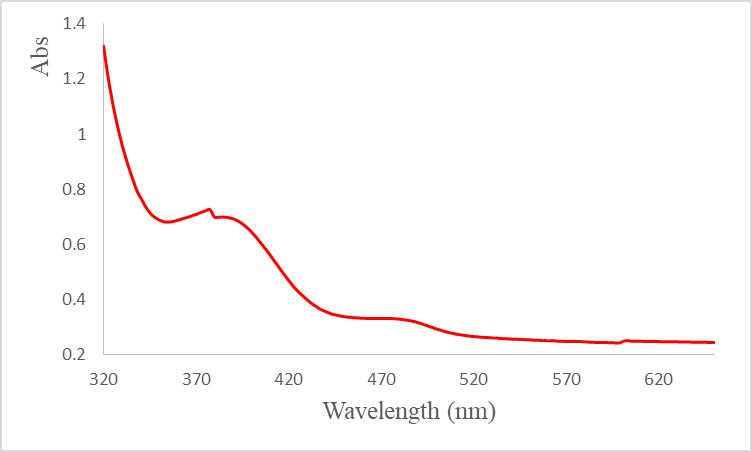
**

**Supplementary Fig. 6.** Absorption Spectra of [(DPEphos)(bcp)Cu]PF_6_

In the range of 380-500 nm, [(DPEphos)(bcp)Cu]PF_6_ showed obvious absorption.

**Stern-Volmer Experiment**

In order to figure out how the excited state of [(DPEphos)(bcp)Cu]PF_6_ interacts with the tethered alcohols and DIPEA, luminescence quenching experiments were performed.

**Experimental procedure**

[(DPEphos)(bcp)Cu]PF_6_ was added in a dried reaction vessel with 2 mL MeCN, 200μl of the solution diluted with 2mL MeCN was added tethered alcohol **1a** (0.01-0.05 M) and DIPEA(0.05-0.25 M) respectively. The solution was transferred to the cuvette with 1 cm optical path utilizing an oven-dried glass Pasteur pipette. Fluorescence spectra of [(DPEphos)(bcp)Cu]PF_6_ (0.25 mM) is given in **Supplementary Fig. 7** and **Fig. 8**. ^6^


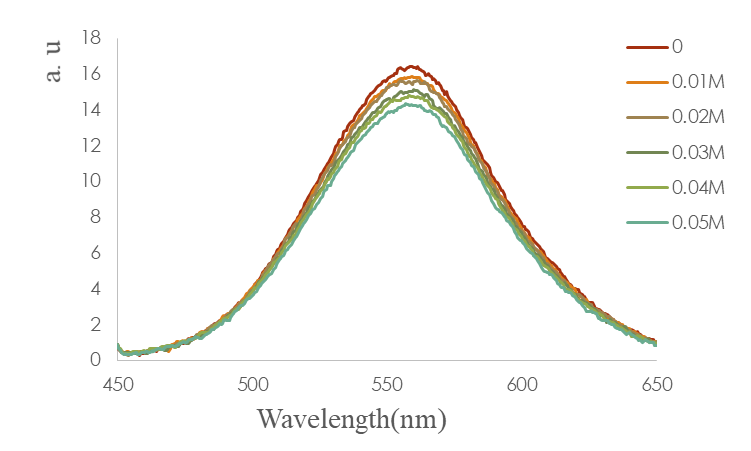


**Supplementary Fig. 7.** Quenching of [(DPEphos)(bcp)Cu]PF_6_* by tethered alcohol **1a**
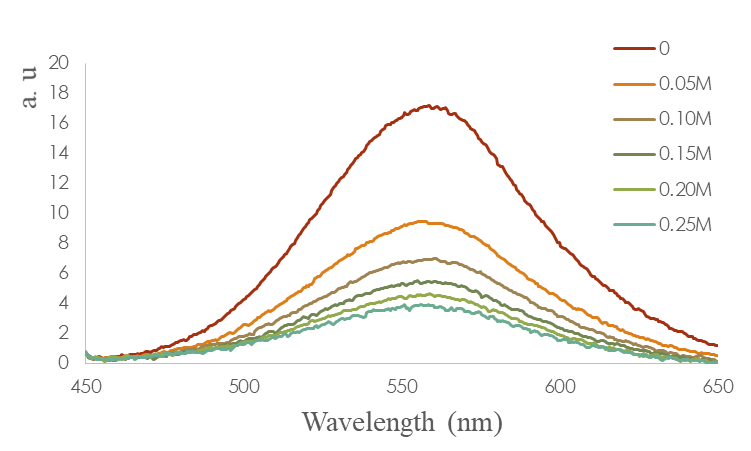


**Supplementary Fig. 8.** Quenching of [(DPEphos)(bcp)Cu]PF_6_* by DIPEA

Stern-Volmer plot for the quenching of [(DPEphos)(bcp)Cu]PF_6_* by tethered alcohol **1a** and DIPEA were showed in **Supplementary Fig. 9** and **Fig. 10** respectively. Based on Stern-Volmer constants, the excited-state [(DPEphos)(bcp)Cu]PF_6_ was quenched faster by the DIPEA than tethered alcohol **1a**.(K_SV(_***_DIPEA_****_)_* = 13.967 M^-1^, K_SV_***_3a_***= 3.012 M^-1^ )


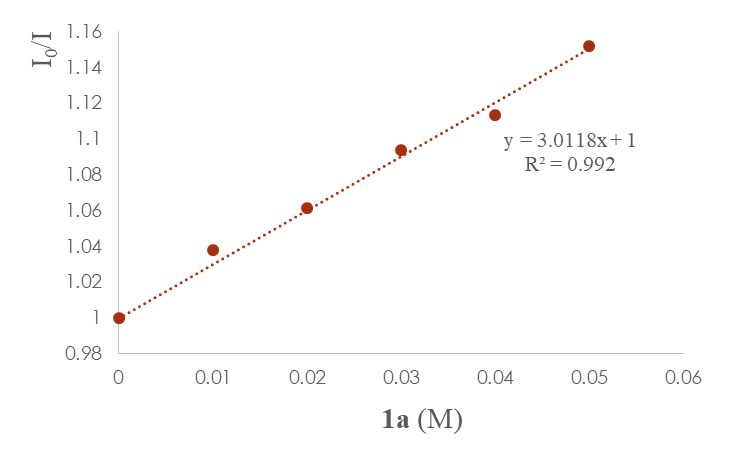


**Supplementary Fig. 9.** The Stern-Volmer plot for the quenching of [(DPEphos)(bcp)Cu]PF6* by **1a**


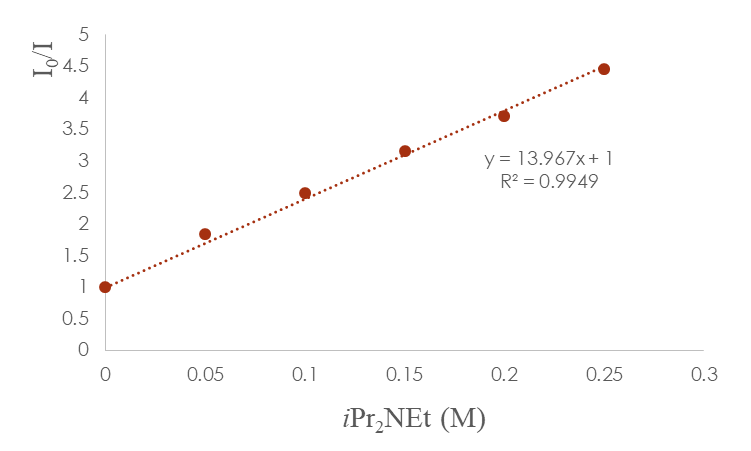


**Supplementary Fig. 10.** The Stern-Volmer plot for the quenching of [(DPEphos)(bcp)Cu]PF6* by DIPEA

1.5.5 Quantum yield measurement

We conducted the quantum yield measurement of the photoinduced reaction between tethered alcohols and terminal alkyne by an optical power meter. The result was calculated based on the product yield and the measured photon flux through the reaction system using the equation $\Phi$= $\frac{\text{mol product}}{\text{photon flux∙t∙f}}$.

**Experimental procedure**

*Product yields and photon flux:*^7^

In a dried sealed vial, [(DPEphos)(bcp)Cu]PF_6_ (0.010 mmol, 10 mol %), DIPEA (0.4 mmol, 4.0 equiv) and terminal alkyne (0.15 mmol, 1.5 equiv) were dissolved in CH_3_CN (1.0 mL) under N_2_ atmosphere. Then tethered alcohol **1a** (0.1 mmol, 1.0 equiv) was added. The reaction mixture was stirred under nitrogen at room temperature under B-LED for 30 min. The distance between the vial and the lamp was about 1-3 cm. The resulting mixture was passed through a pad of silica gel, and concentrated under a reduced pressure. Diethyl phthalate was internal standard for ^1^H NMR suggesting a 2.4 × 10^-5^ mol product (24 % yield). The illumination intensity of the reaction position was determined by an optical power meter to be 165.6 mW/cm^2^. The irradiated area S was measured to be 4.7 cm^2^. Based on equation (1), the Photon flux was calculated to be 3.03×10^-6^ einstein·s^-1^

Photon flux = $\frac{P}{N_{A}\cdot{hc}/\lambda}$ = $\frac{165.6\times{10}^{-3}\times4.7}{6.02\times{10}^{23}\times6.63\times{10}^{-34}\times3\times{{10}^{8}}/{(467\times{10}^{-9})}}$ = 3.03×10^-6^ einstein·s^-1^ (1)

N_A_: 6.02×10^23^/mol; λ: wavelength (λ = 467 nm); h: planck constant (h = 6.626 × 10^-34^ J*s); c: velocity of light (c = 3 × 10^8^ m/s); P = E*S (E: illumination intensity, E = 165.6 mW/cm^2^; S: the irradiated area S = 4.7 cm^2^.

*Fraction of light (f)* ^8,9^

In a parallel reaction, in a glove box, [(DPEphos)(bcp)Cu]PF_6_ (0.010 mmol, 10 mol %), DIPEA (0.4 mmol, 4.0 equiv) and terminal alkyne (0.15 mmol, 1.5 equiv) were dissolved in CH_3_CN (1.0 mL). Then tethered alcohol **1a** (0.1 mmol, 1.0 equiv) was added. The tube was sealed with Teflon septum and removed from glovebox. The reaction mixture was stirred under nitrogen at room temperature under B-LED for 30 min. After irradiation, the tube was moved into glovebox and 0.1 mL of the solution diluted with 2 mL MeCN, the solution was transferred to the cuvette with 1 cm optical path utilizing an oven-dried glass Pasteur pipette. The UV-Vis spectra of the reaction system was measured directly in MeCN_,_The absorbance at 467 nm is 0.295, so the A value of the reaction system should be 5.9 indicating the fraction of light f value is >0.999 according to the equation (2).

f = 1 – 10^-A^ = 1 – 10^-5.9^ = 0.999 (2)

Thus, the quantum yield can be calculated by the equation (3). f is the fraction of light (f = 0.999, λ = 467 nm), the production of 2.4 × 10^-5^ mol product (24 % yield) in 30 min (1800 s) corresponds to a quantum yield $\Phi$= 0.44%.

$\Phi$= $\frac{\text{mol product}}{\text{photon flux∙t∙f}}$ = $\frac{2.4\times{10}^{-5}}{3.03\times{10}^{-6}\times1800\times0.999}$ = 0.44% (3)

- 1. Spectral data


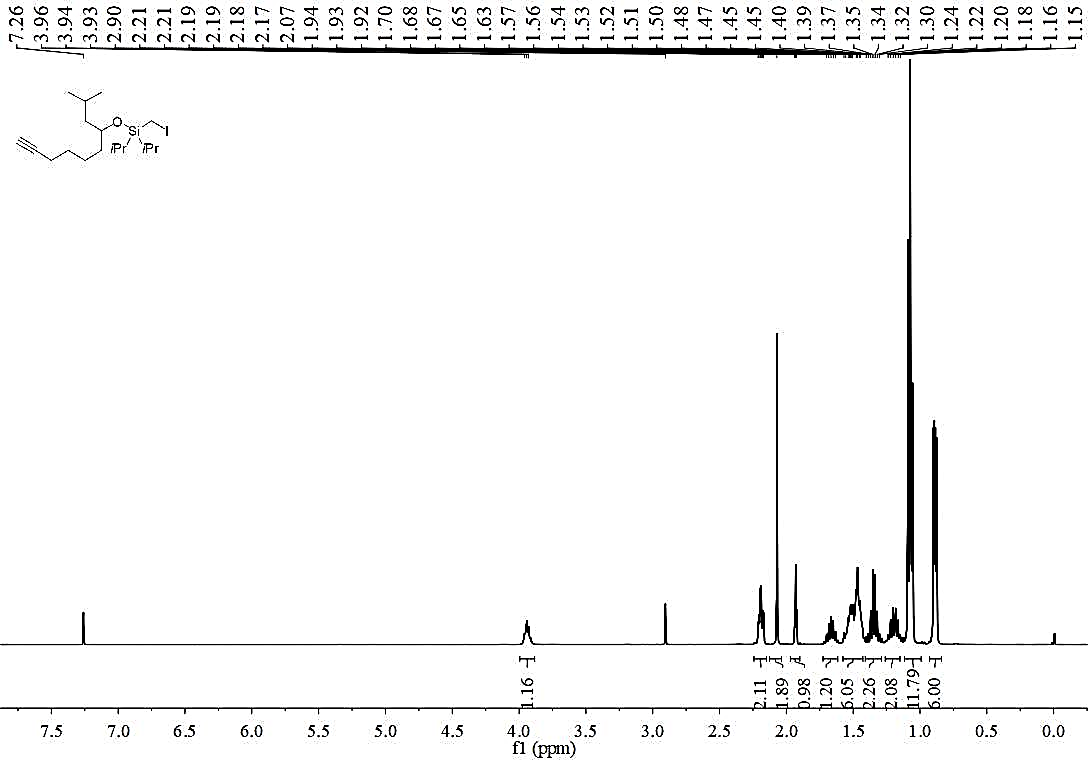


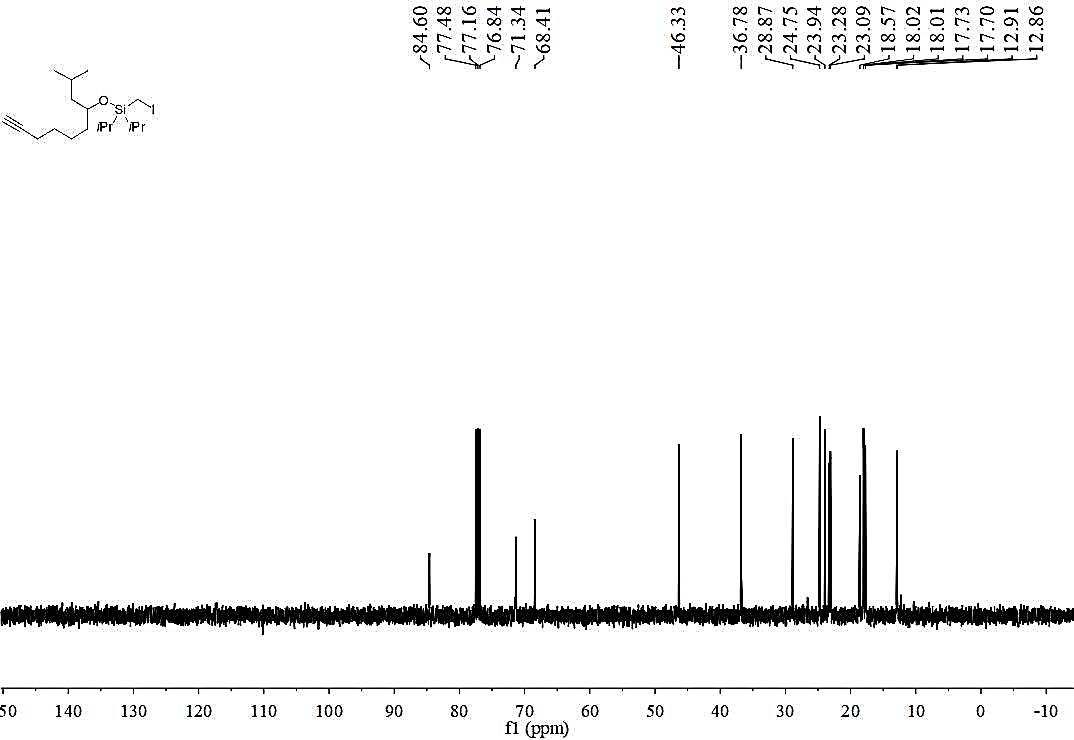


**Supplementary Fig. 11.** ^1^H NMR and ^13^C NMR spectrum of **1g.**


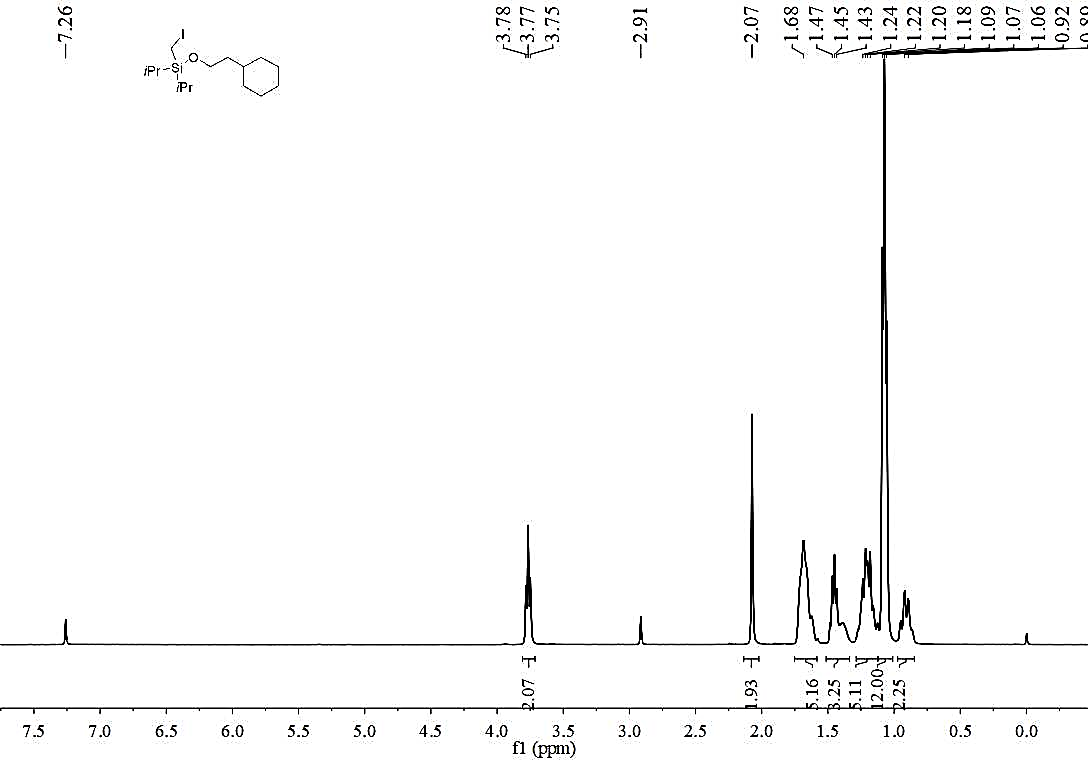


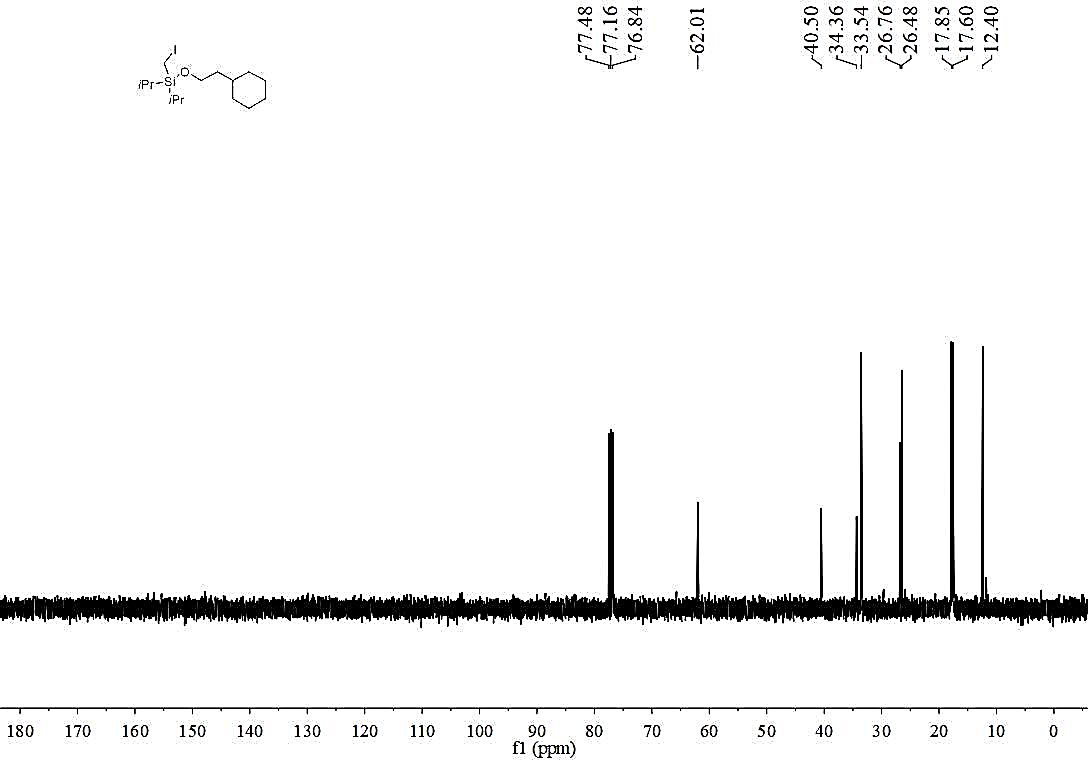


**Supplementary Fig. 12.** ^1^H NMR and ^13^C NMR spectrum of **1s.**


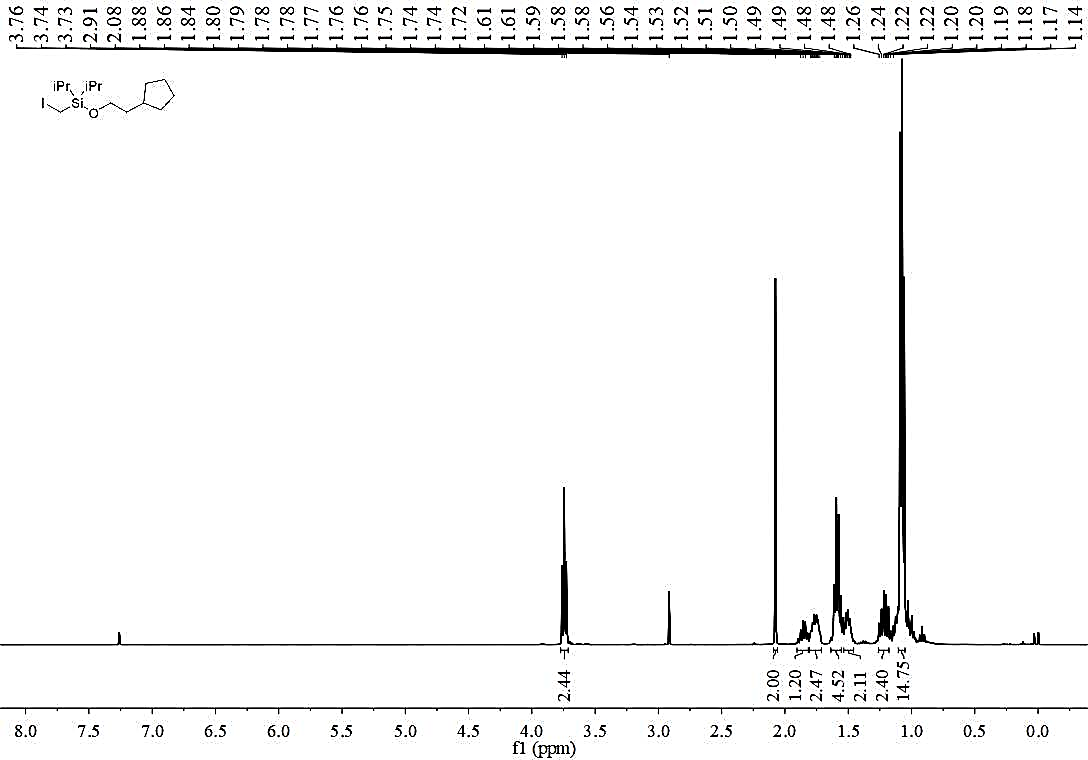


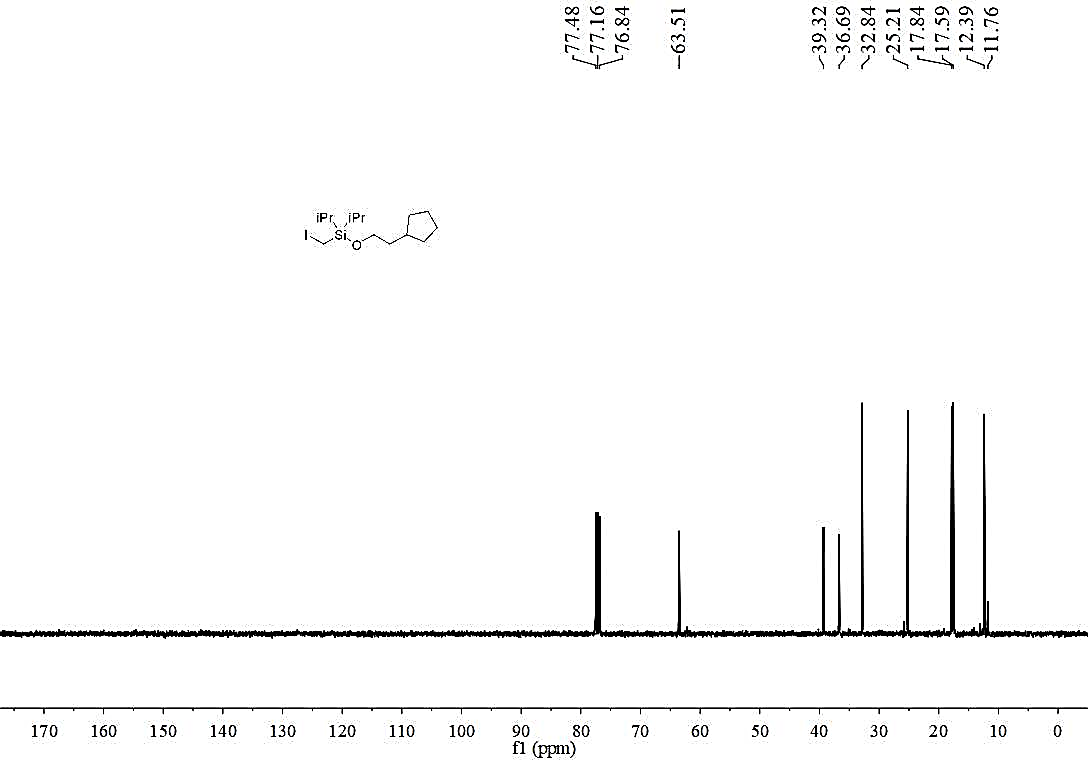


**Supplementary Fig. 13.** ^1^H NMR and ^13^C NMR spectrum of **1t.**


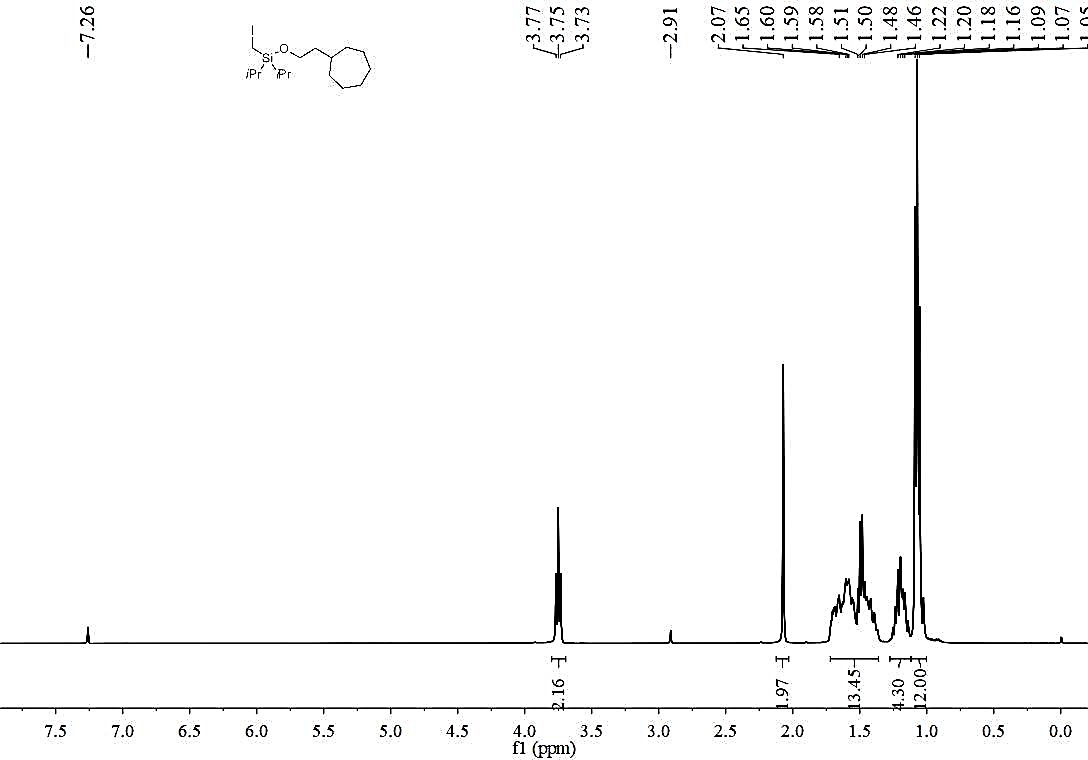


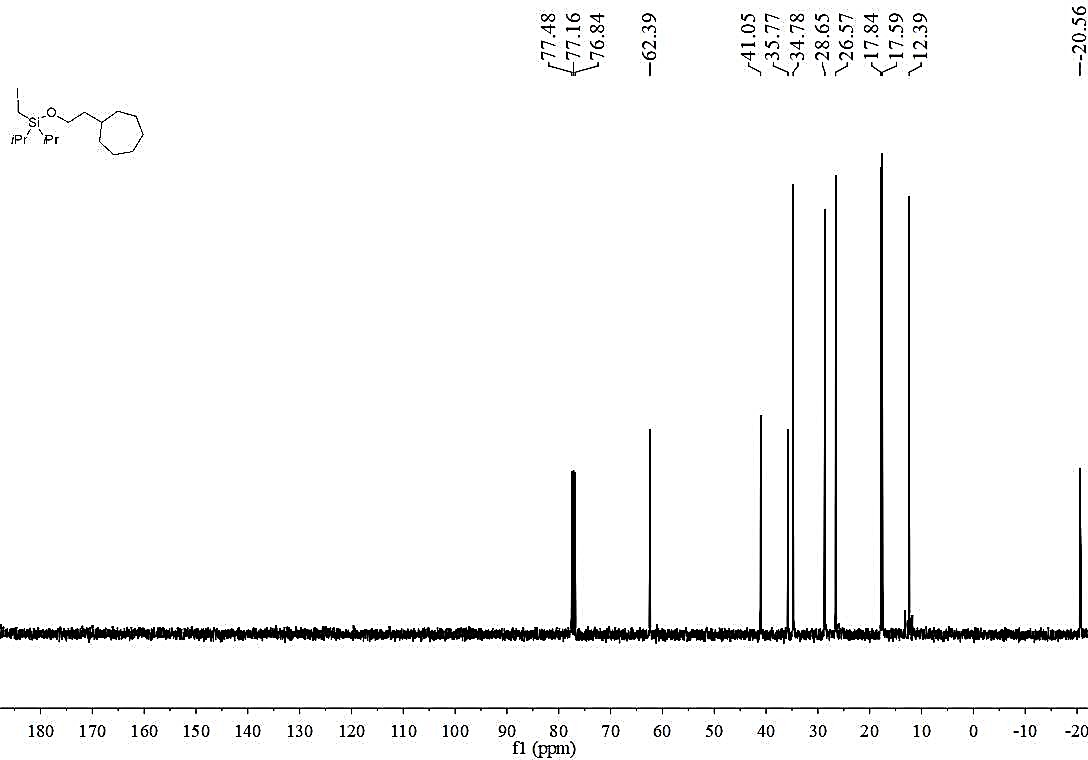


**Supplementary Fig. 14.** ^1^H NMR and ^13^C NMR spectrum of **1u.**


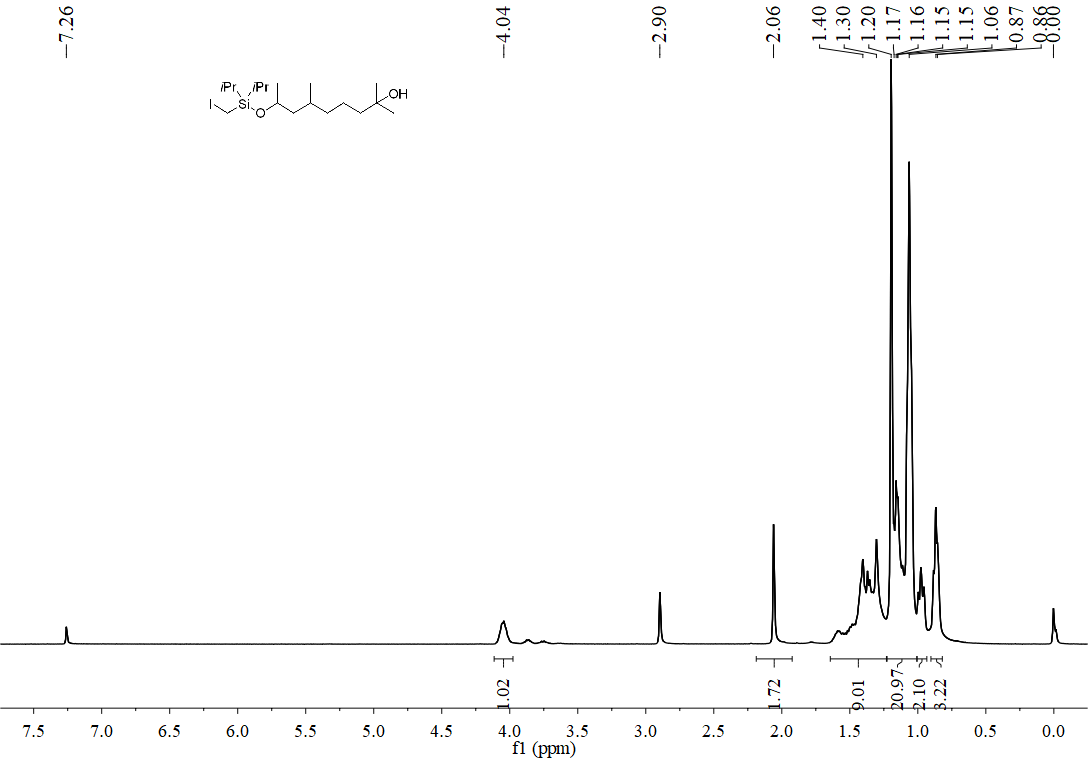


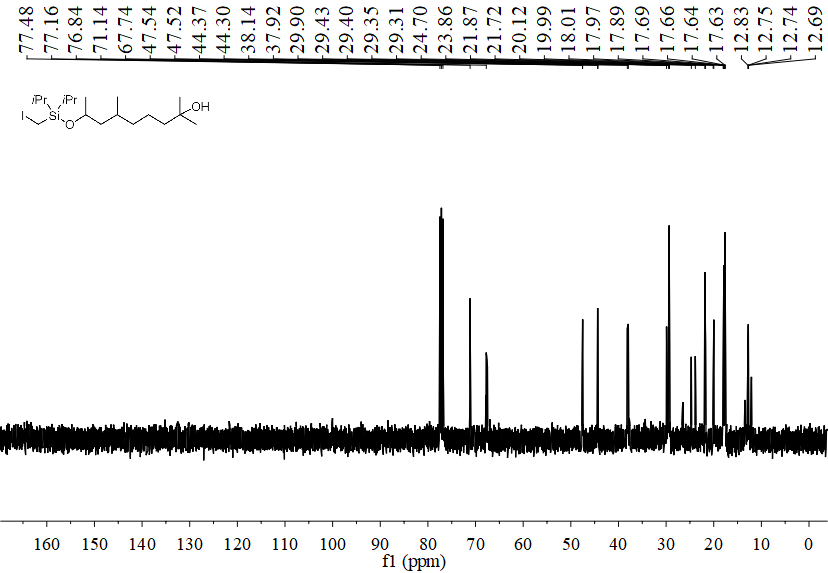


**Supplementary Fig. 15.** ^1^H NMR and ^13^C NMR spectrum of **1v.**


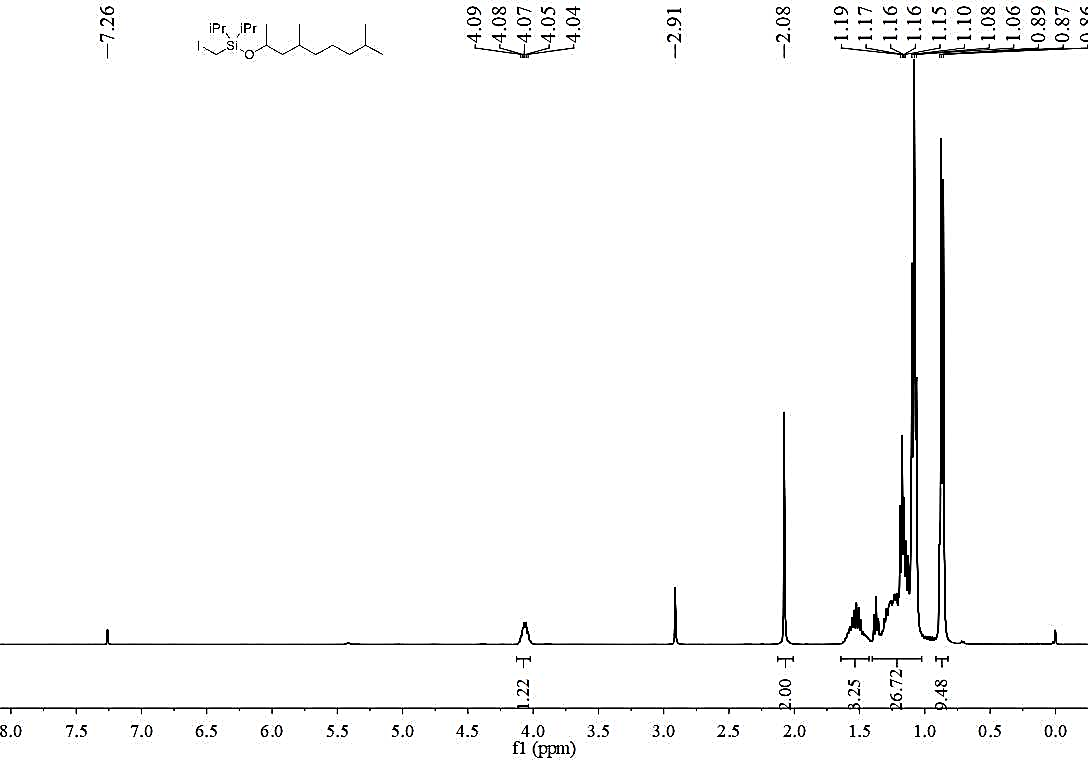


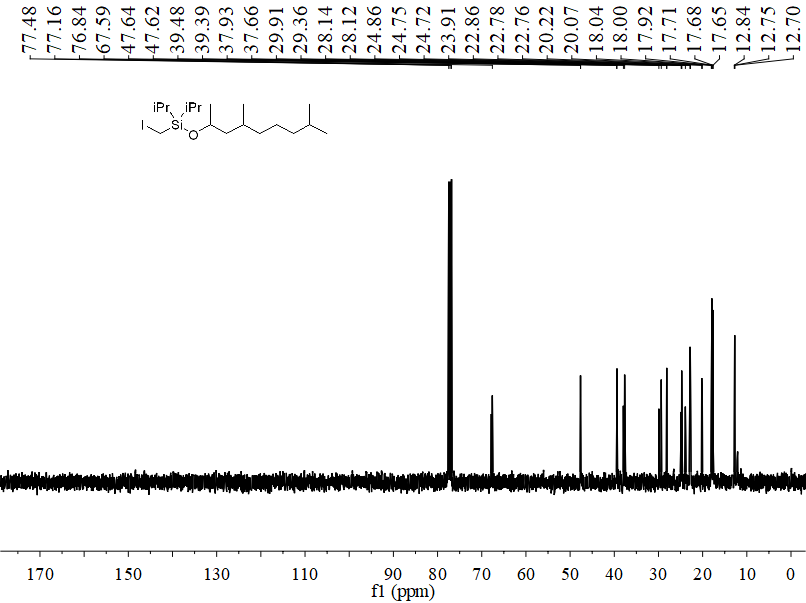


**Supplementary Fig. 16.** ^1^H NMR and ^13^C NMR spectrum of **1w.**


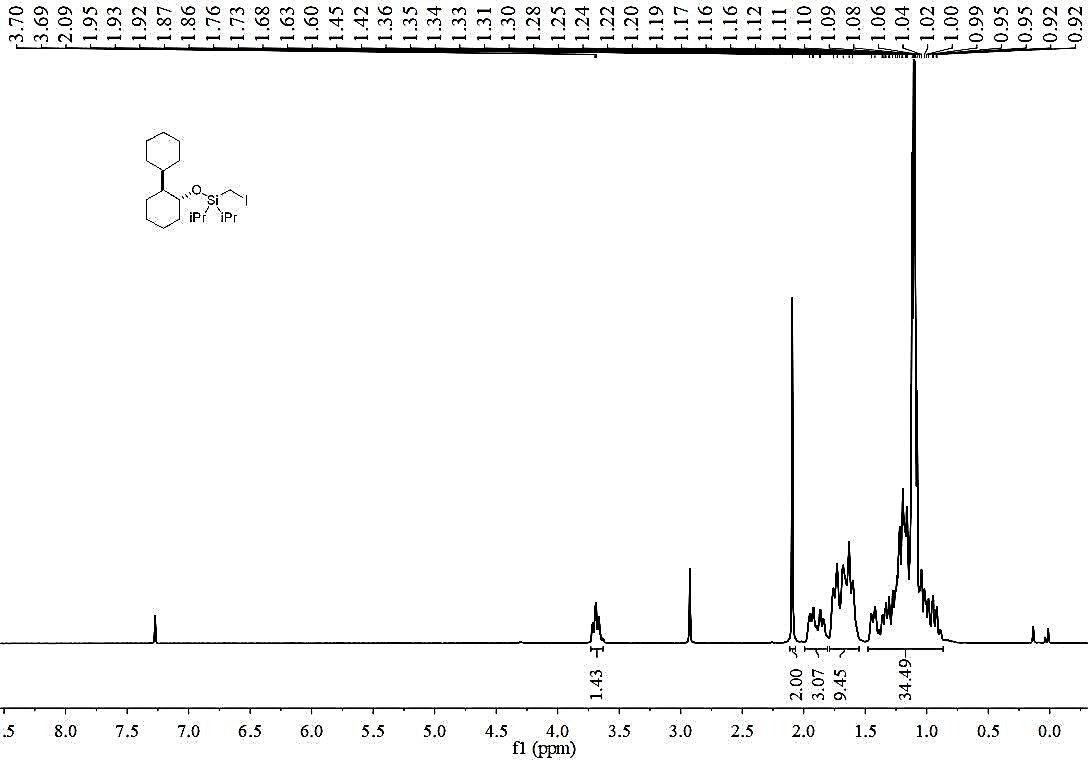


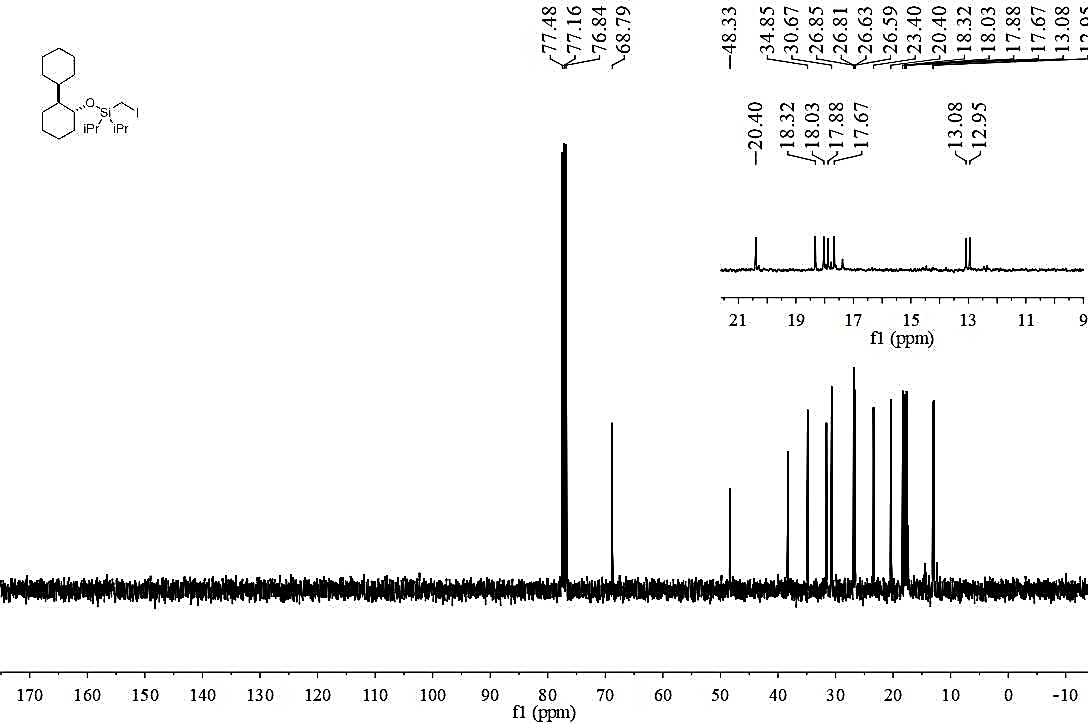


**Supplementary Fig. 17.** ^1^H NMR and ^13^C NMR spectrum of **1z.**


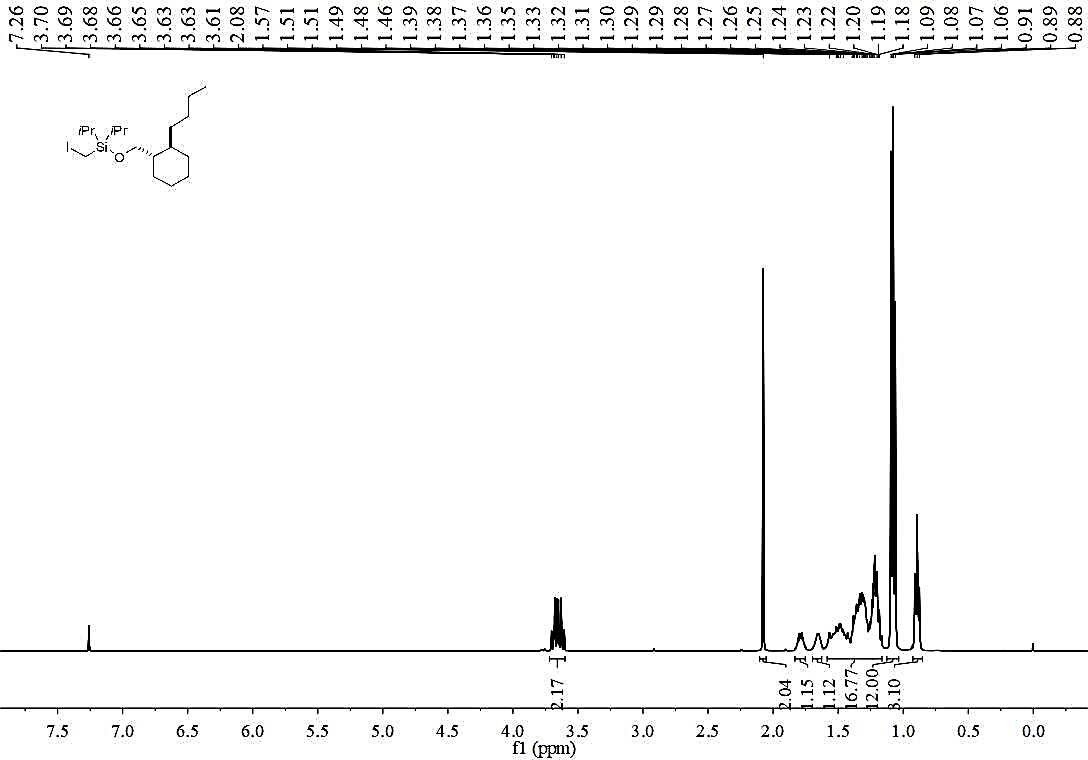


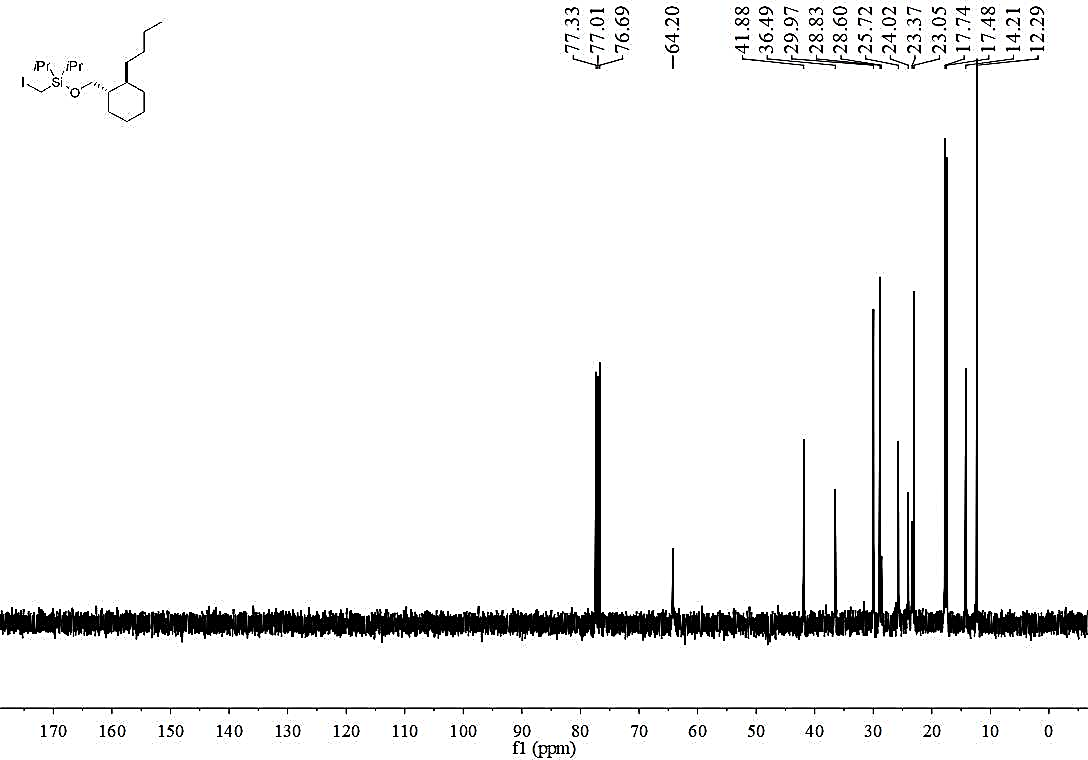


**Supplementary Fig. 18.** ^1^H NMR and ^13^C NMR spectrum of **1aa.**

**
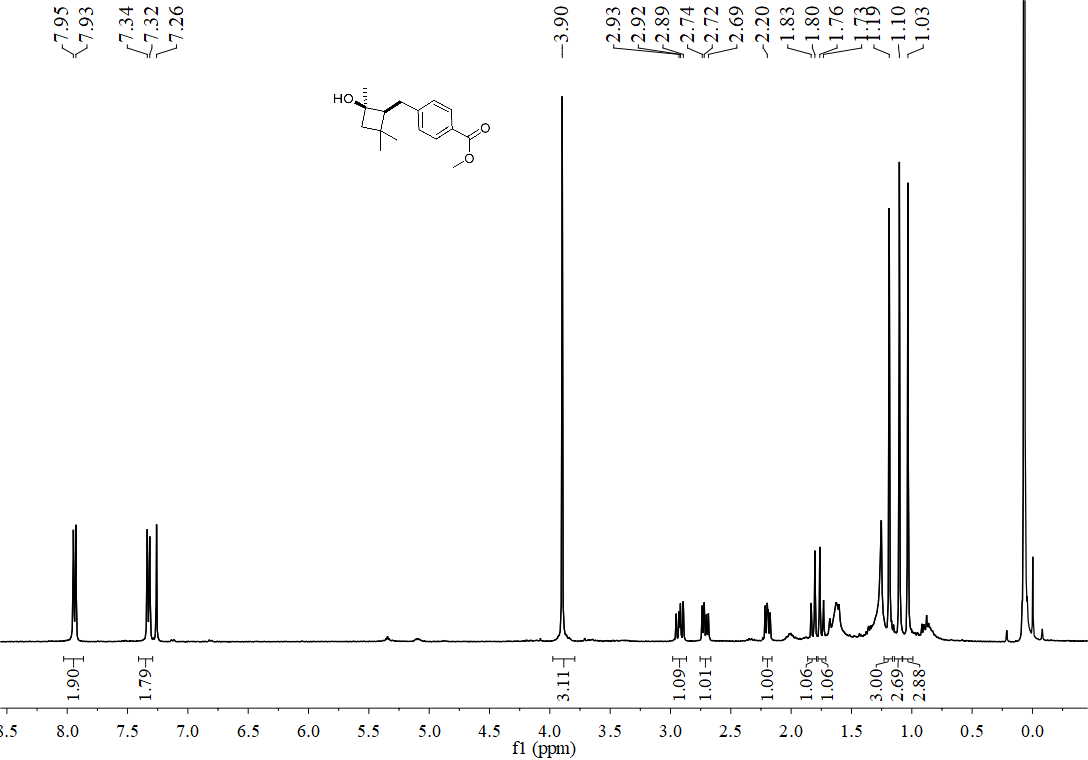
**

**
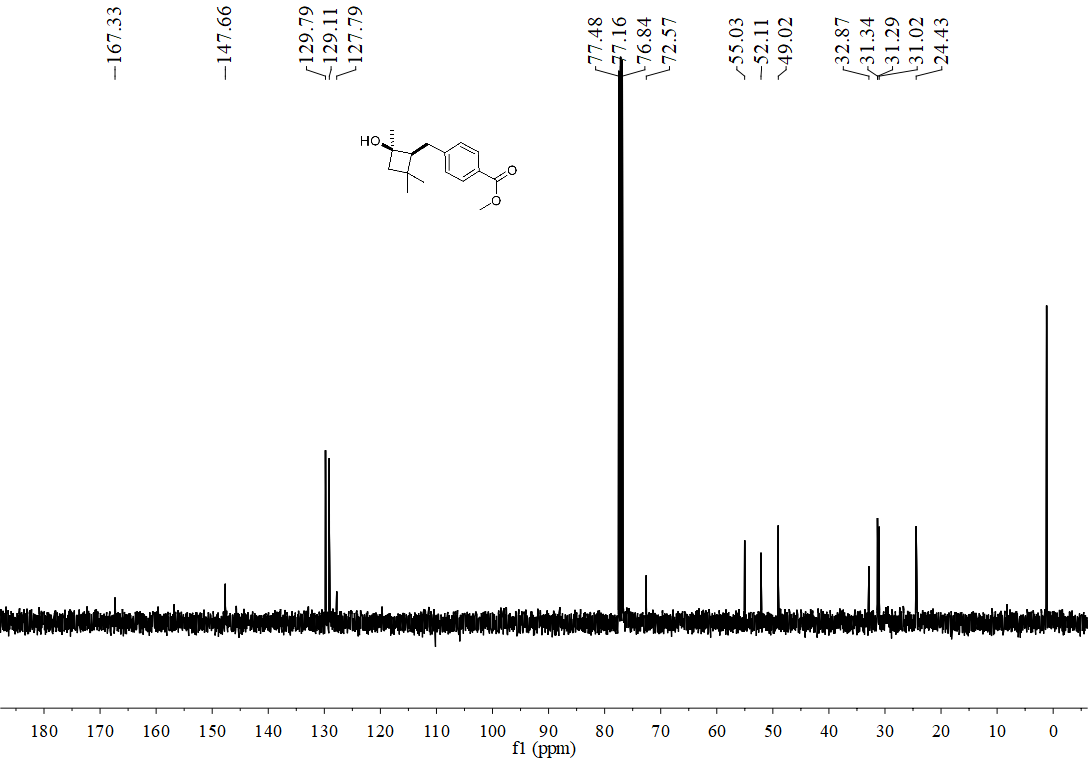
**

**Supplementary Fig. 19.** ^1^H NMR and ^13^C NMR spectrum of **3a_1_**.

**
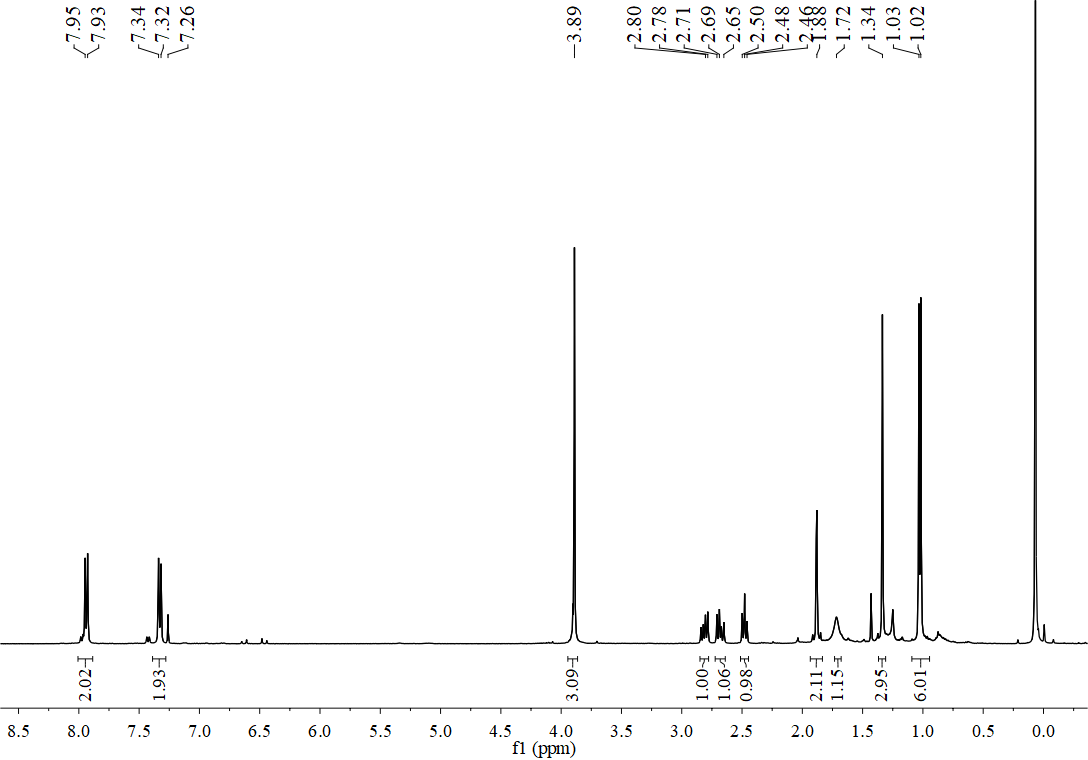
**

**
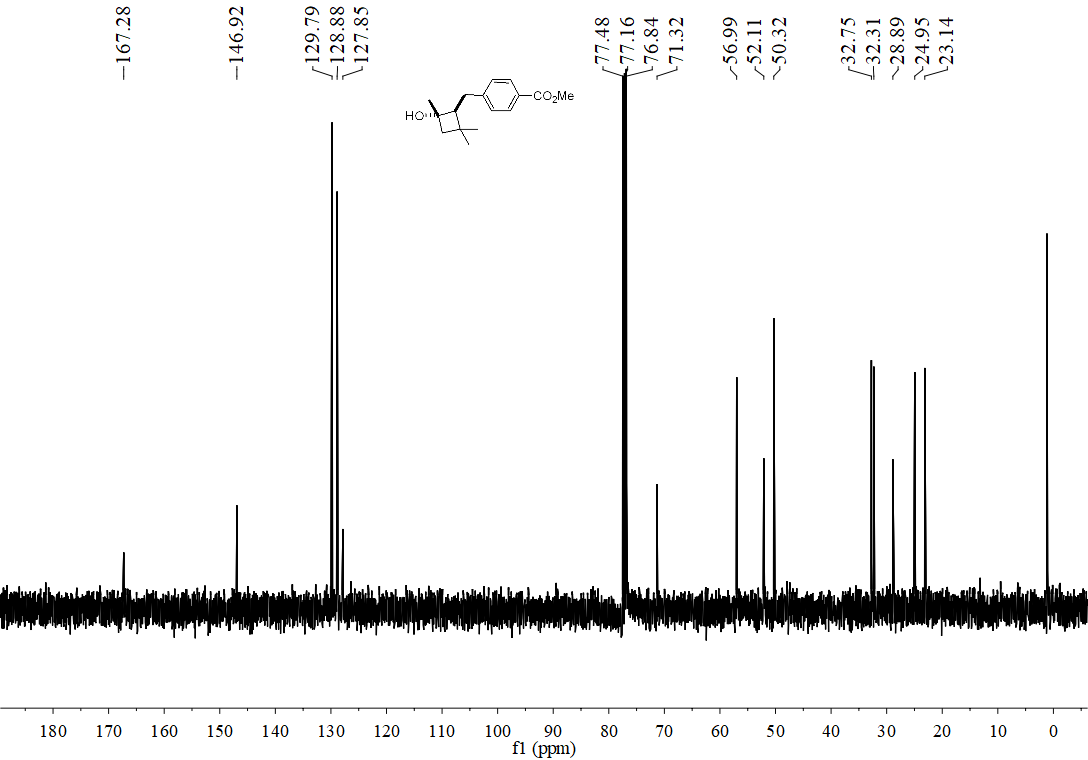
**

**Supplementary Fig. 20.** ^1^H NMR and ^13^C NMR spectrum of **3a_2_**.

**
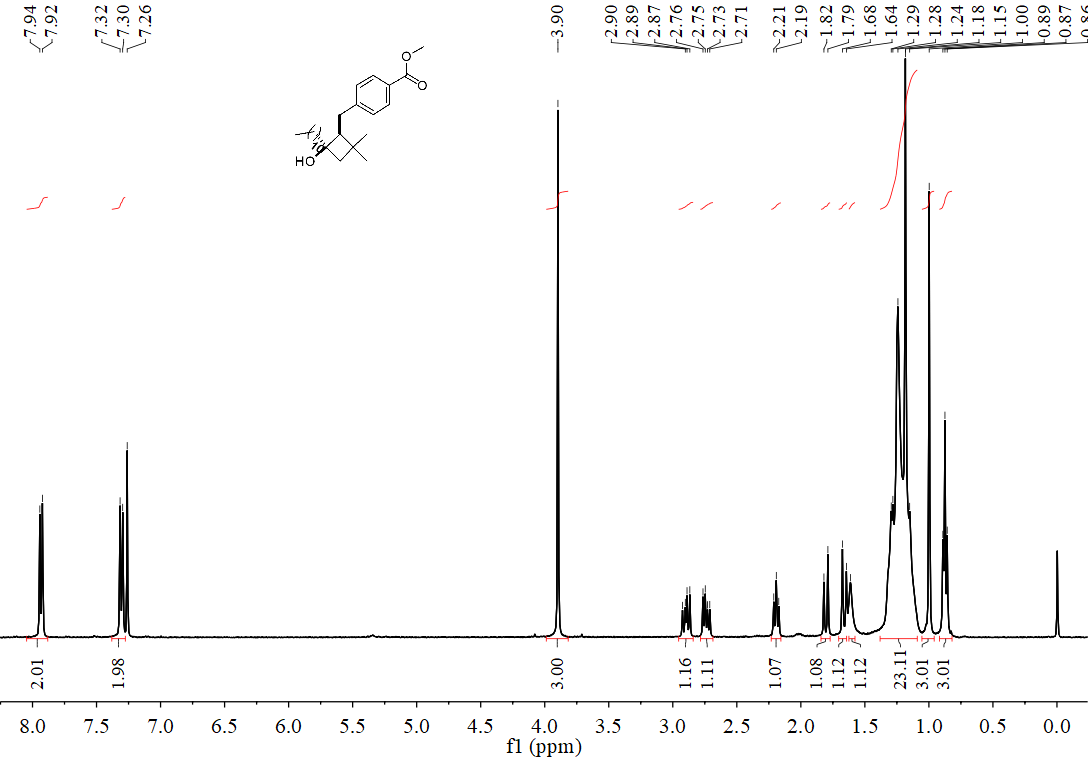
**

**
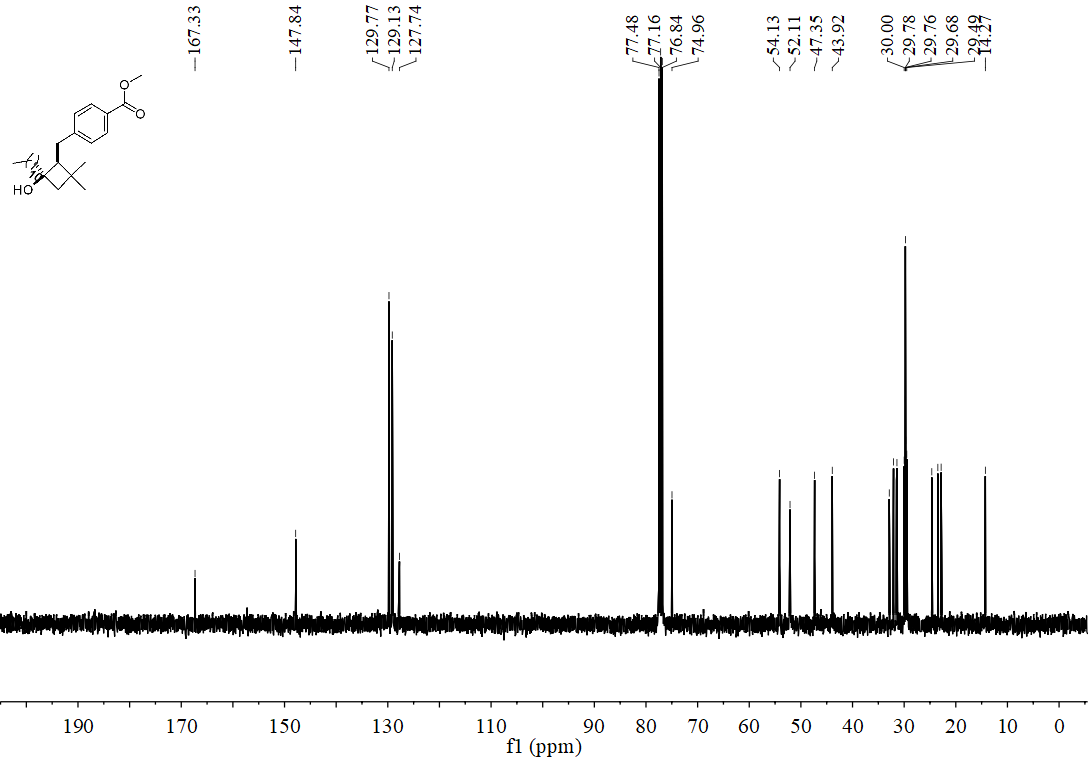
**

**Supplementary Fig. 21.** ^1^H NMR and ^13^C NMR spectrum of **3b_1_**.

**
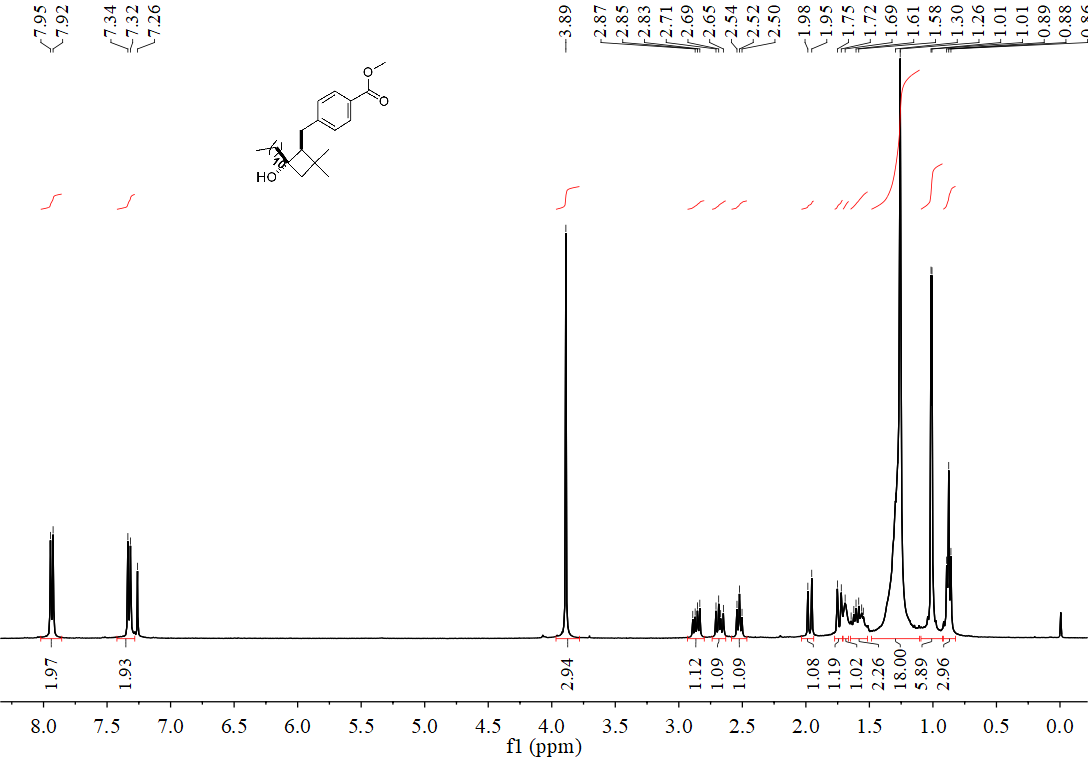
**

**
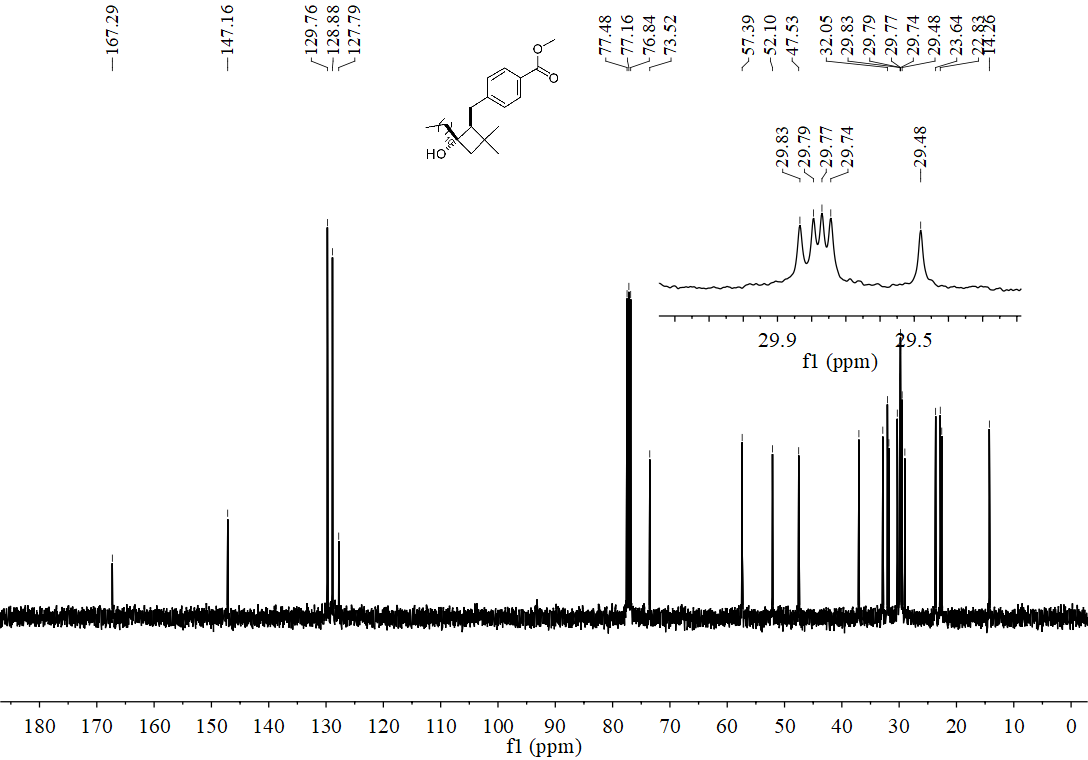
**

**Supplementary Fig. 22.** ^1^H NMR and ^13^C NMR spectrum of **3b_2_**.

**
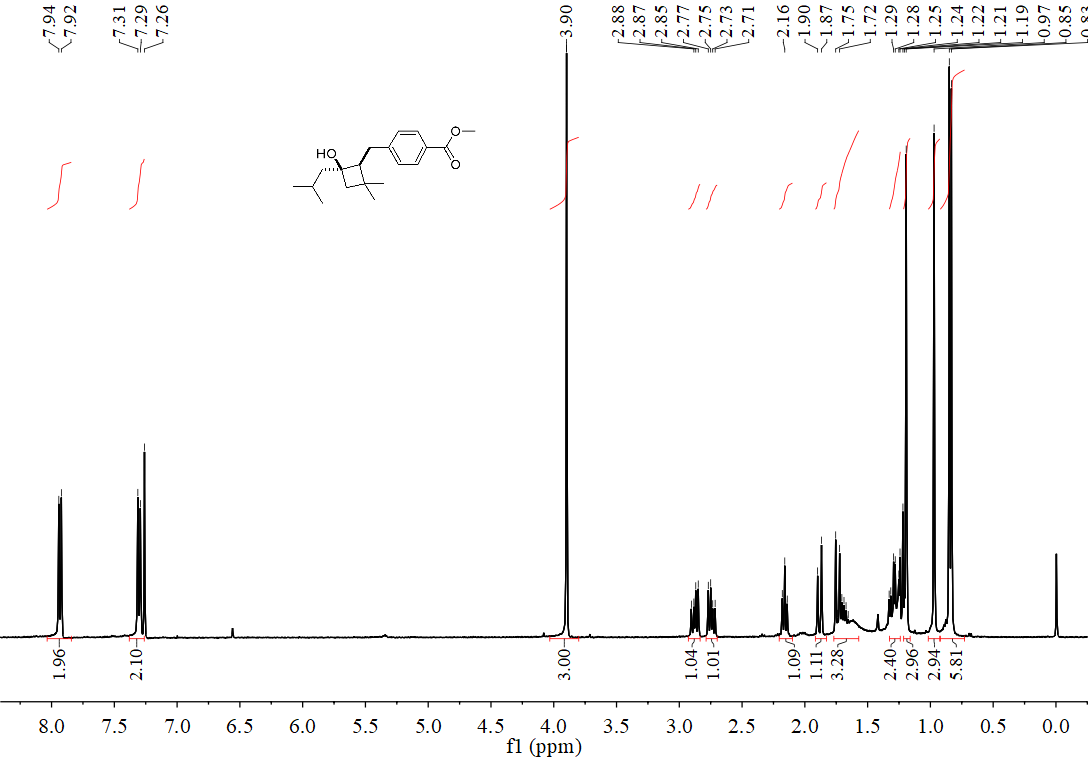
**

**
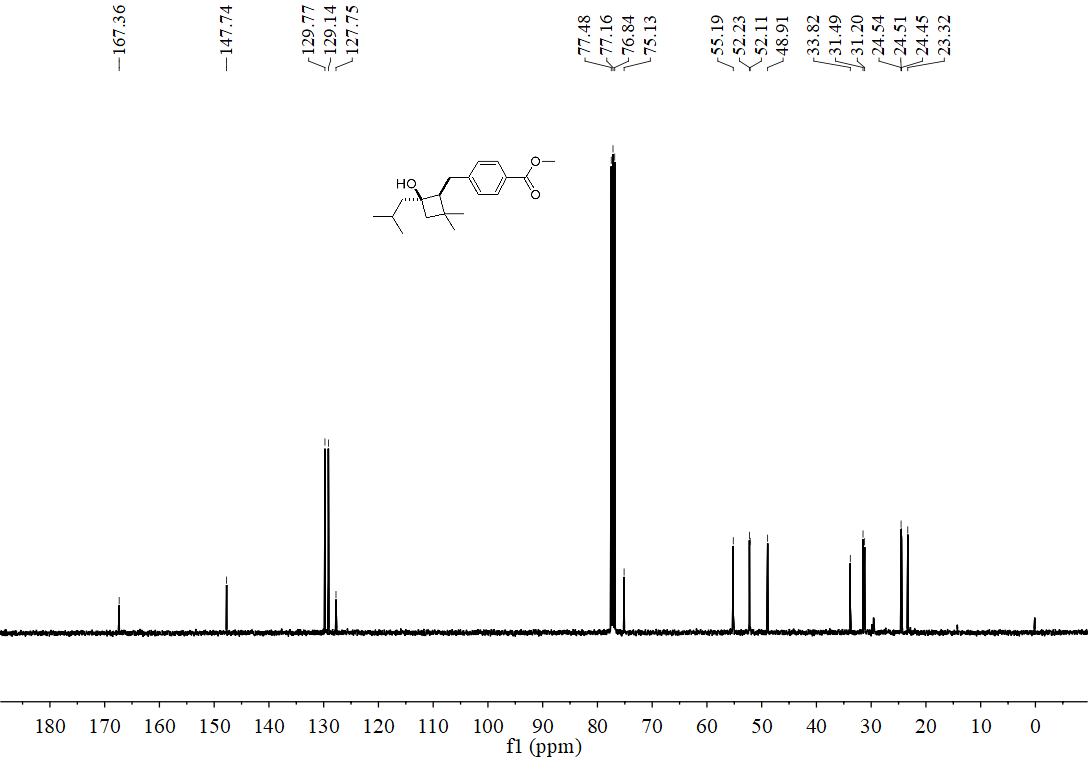
**

**Supplementary Fig. 23.** ^1^H NMR and ^13^C NMR spectrum of **3c_1_**.

**
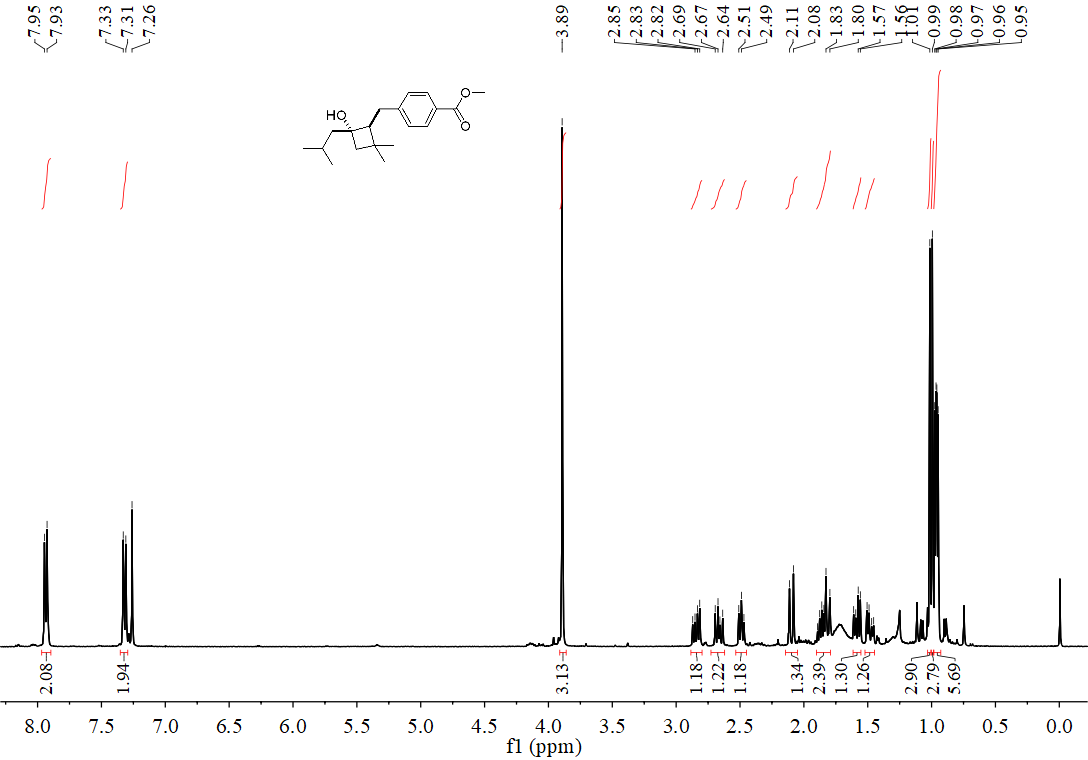
**

**
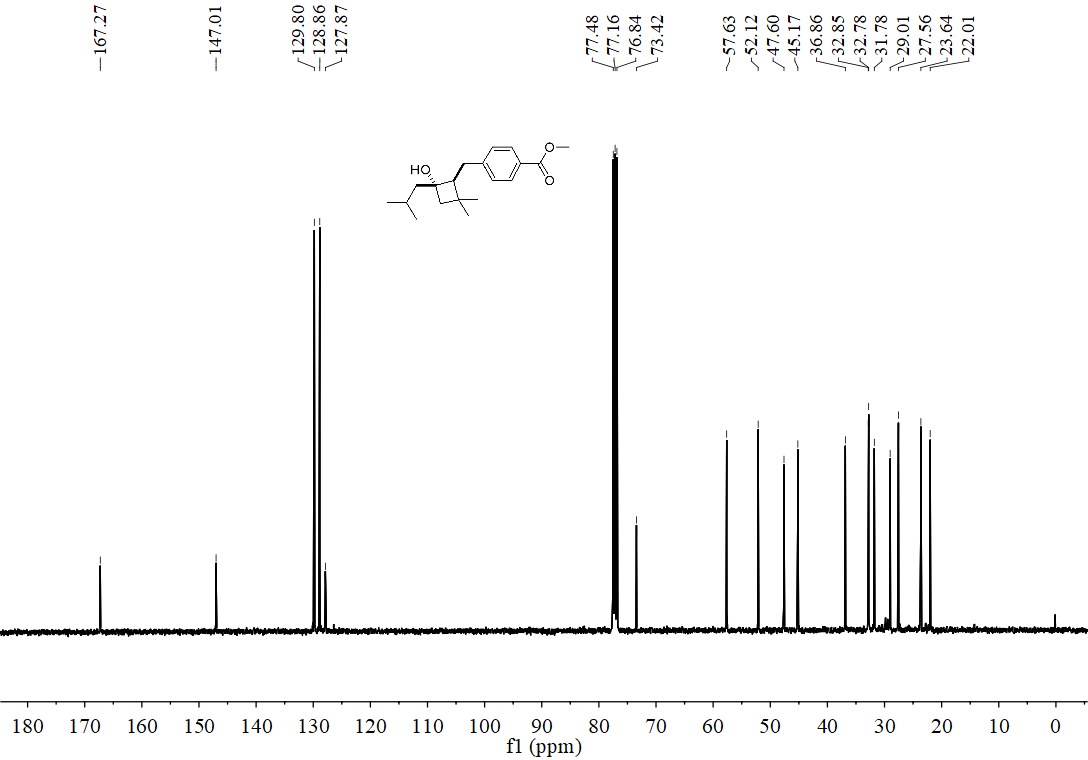
**

**Supplementary Fig. 24.** ^1^H NMR and ^13^C NMR spectrum of **3c_2_**.

**_
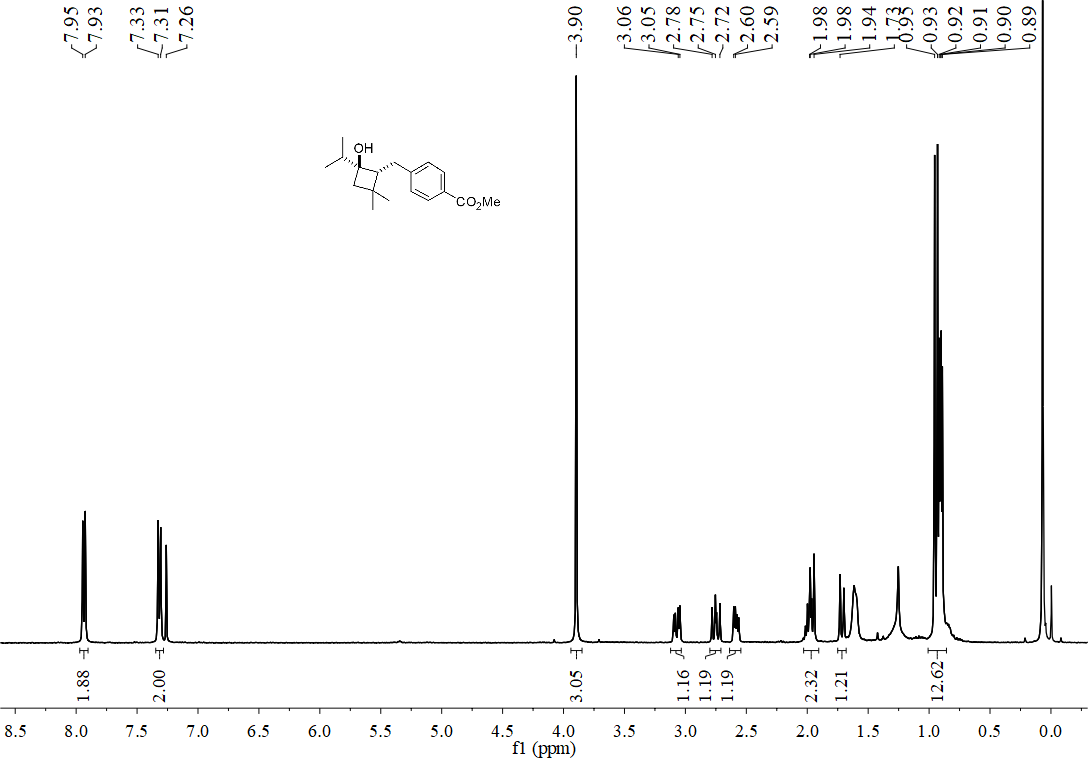
_**

**_
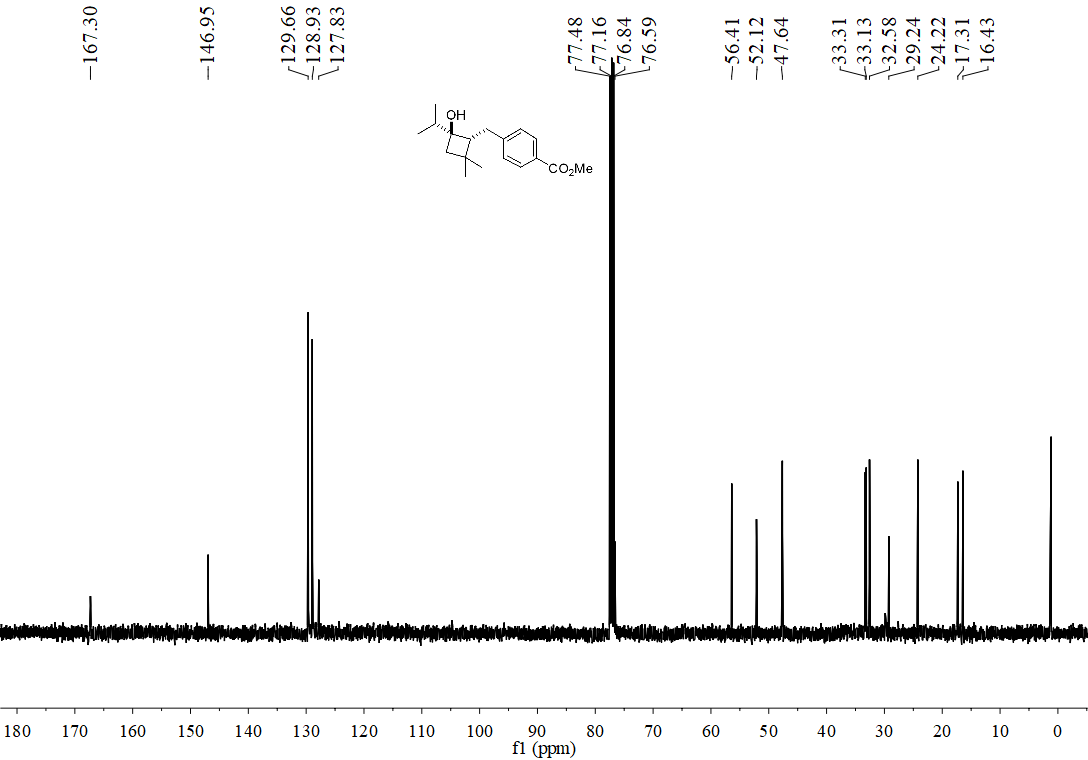
_**

**Supplementary Fig. 25.** ^1^H NMR and ^13^C NMR spectrum of **3d**.

**
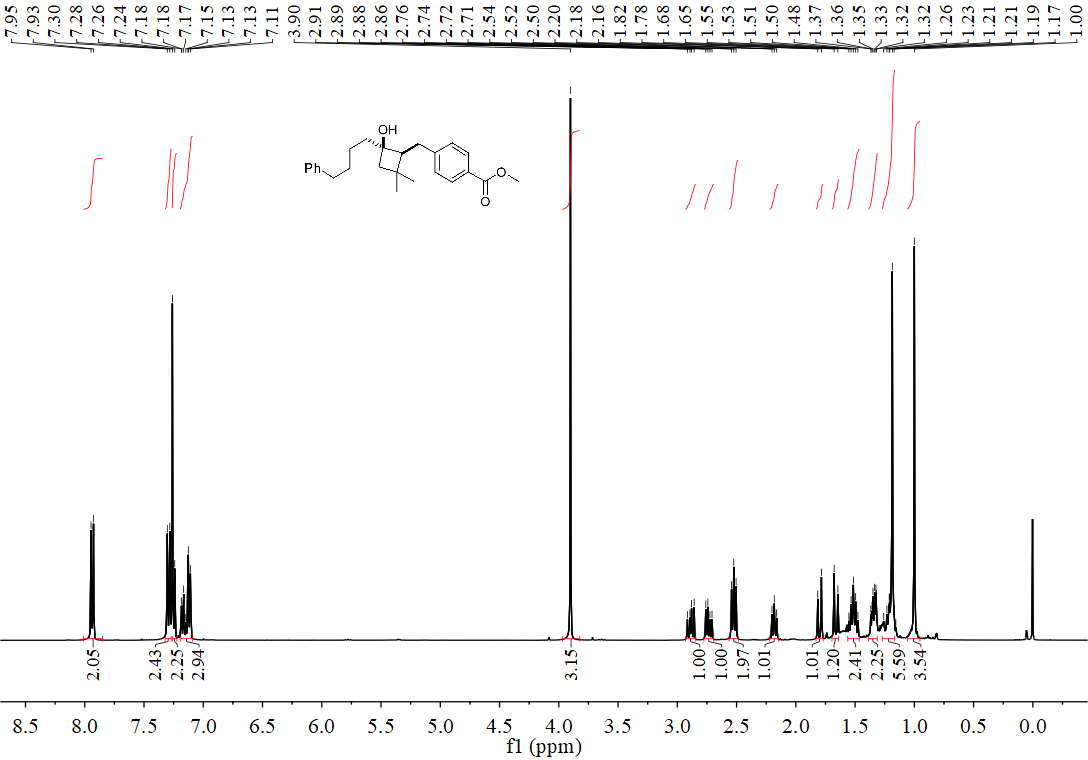
**

**
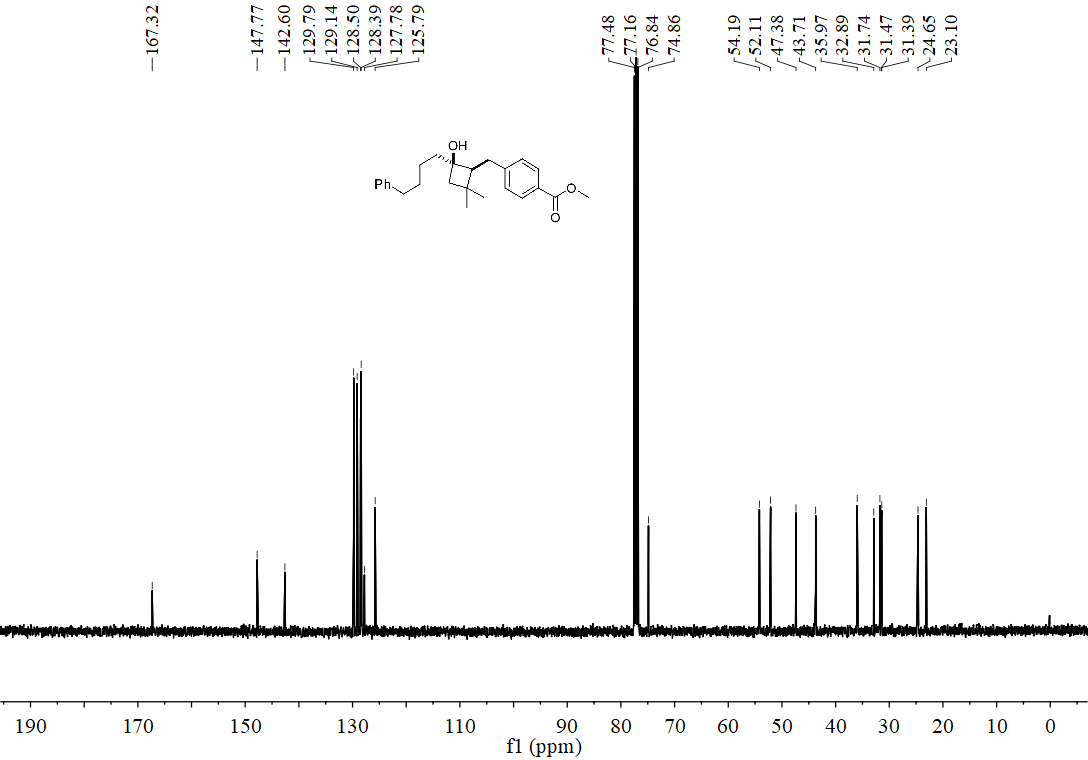
**

**Supplementary Fig. 26.** ^1^H NMR and ^13^C NMR spectrum of **3e_1_**.

**
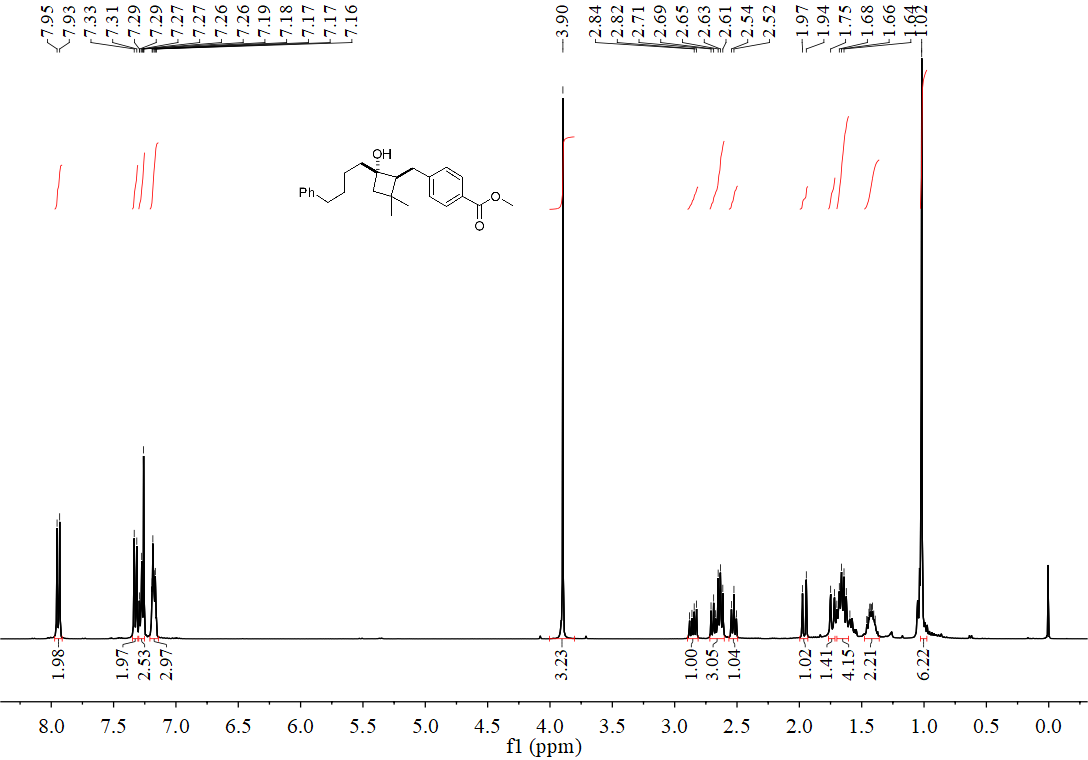
**

**
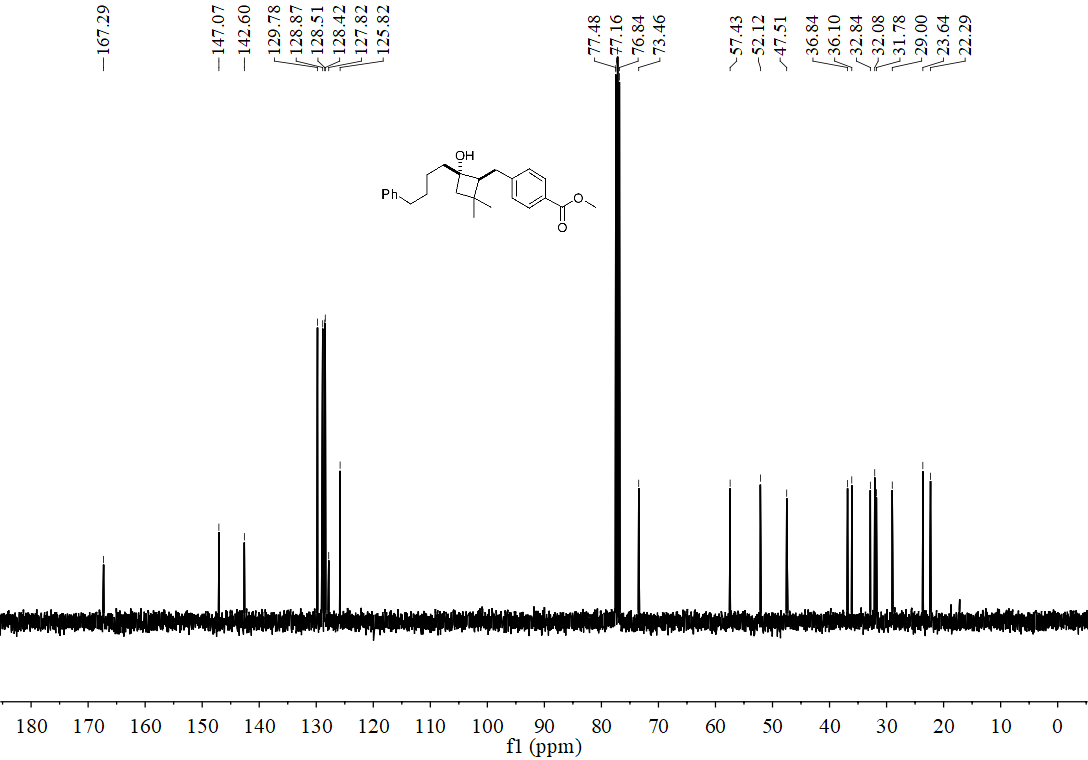
**

**Supplementary Fig. 27.** ^1^H NMR and ^13^C NMR spectrum of **3e_2_**.

**
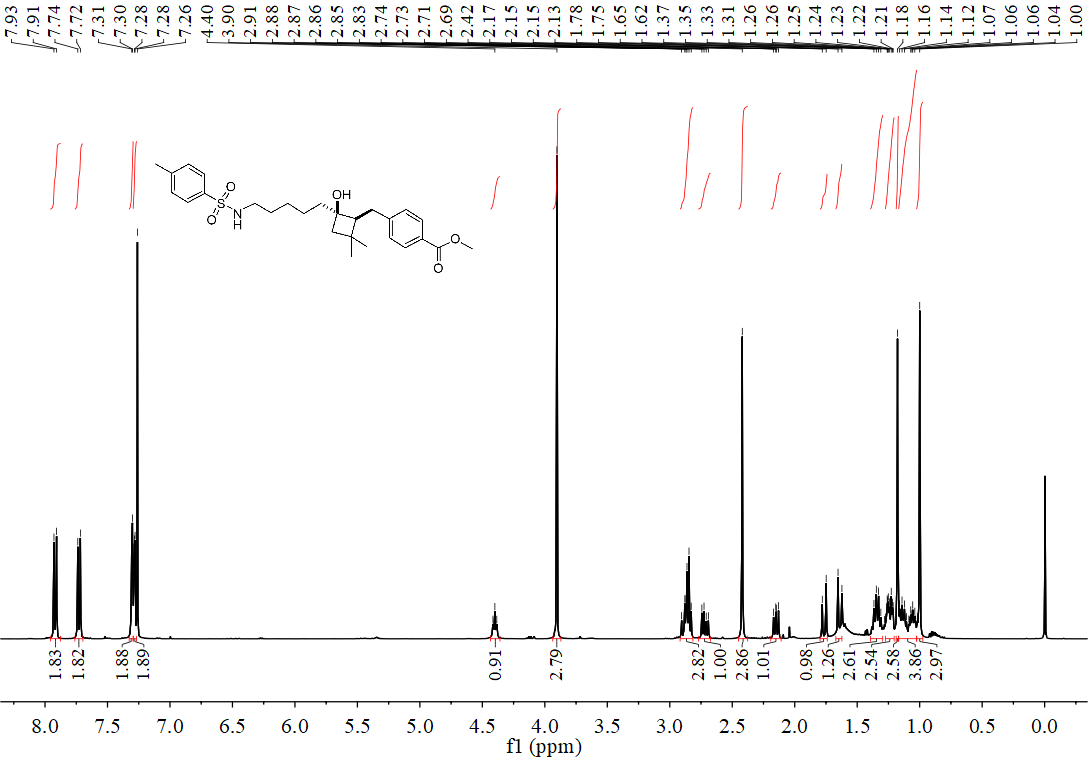
**

**
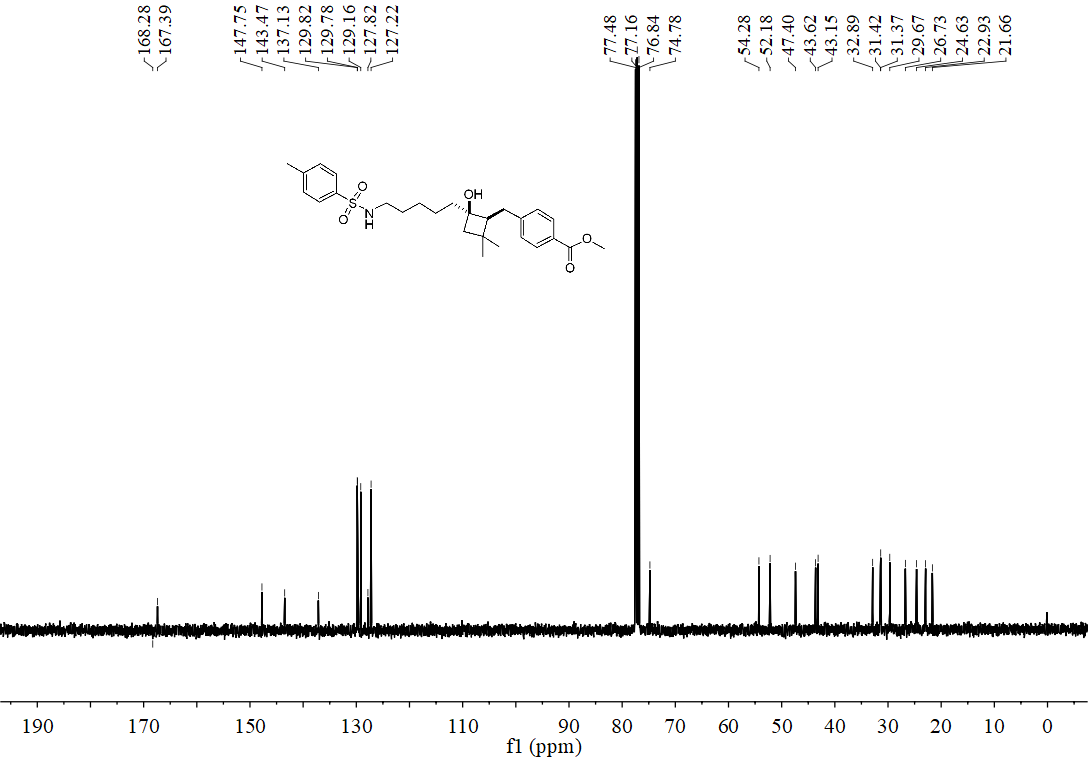
**

**Supplementary Fig. 28.** ^1^H NMR and ^13^C NMR spectrum of **3f_1_**.

**
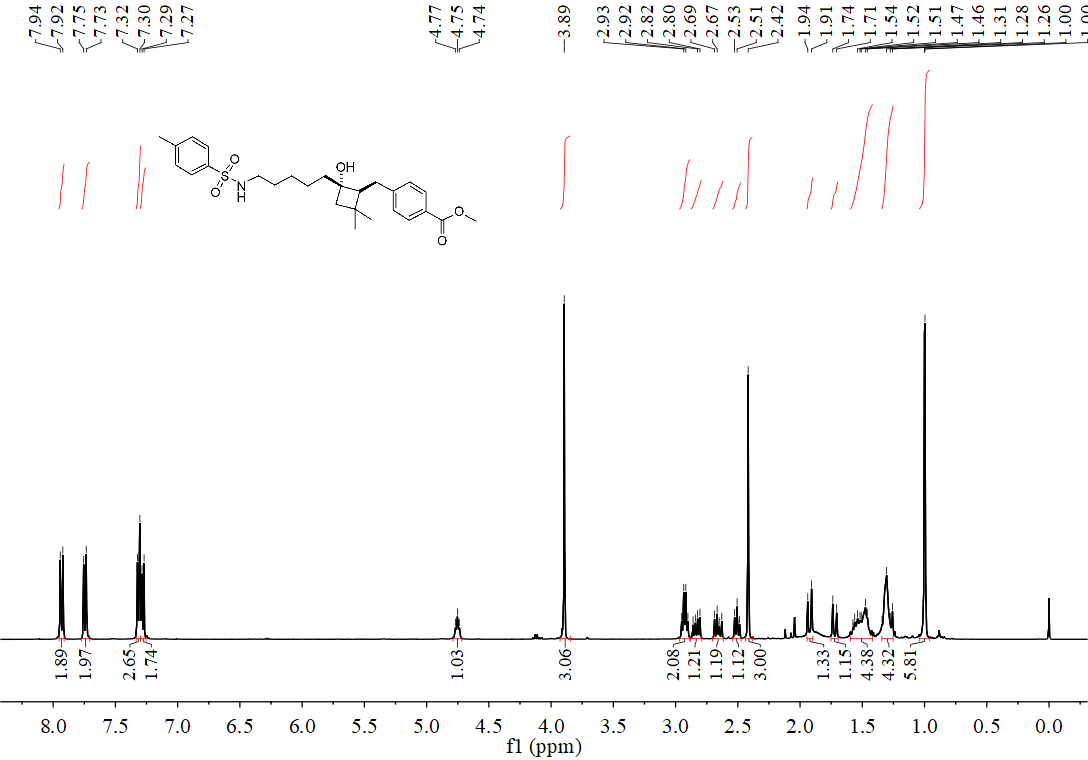
**

**
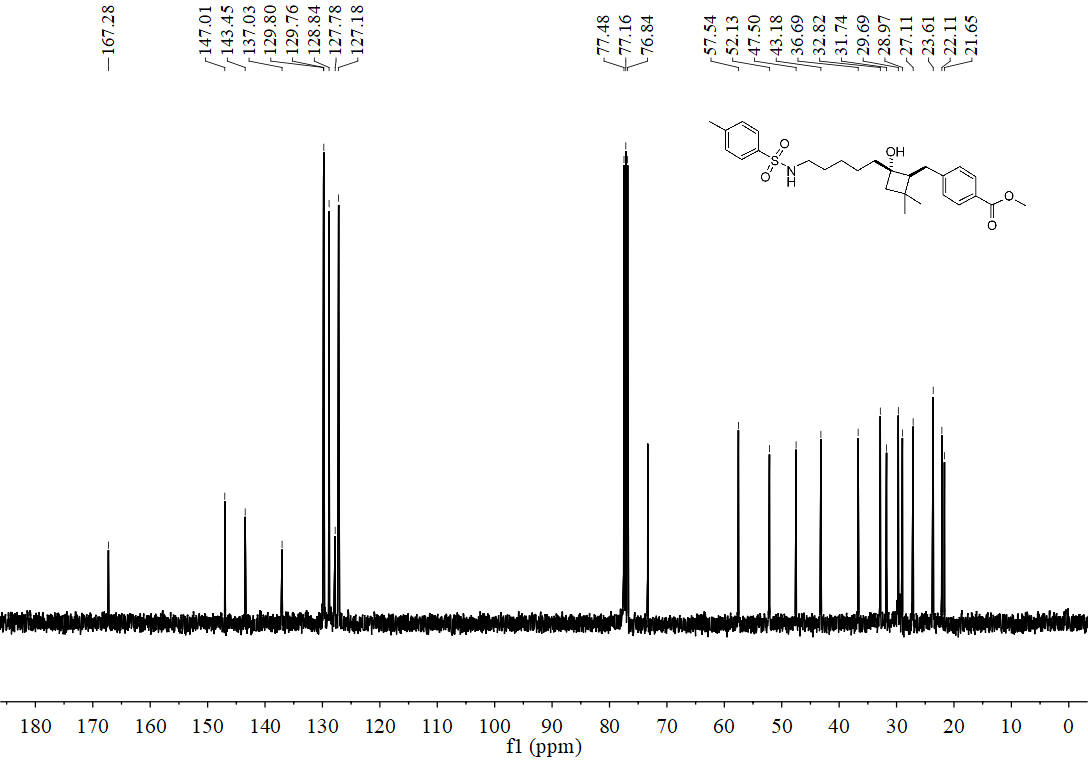
**

**Supplementary Fig. 29.** ^1^H NMR and ^13^C NMR spectrum of **3f_2_**.

**
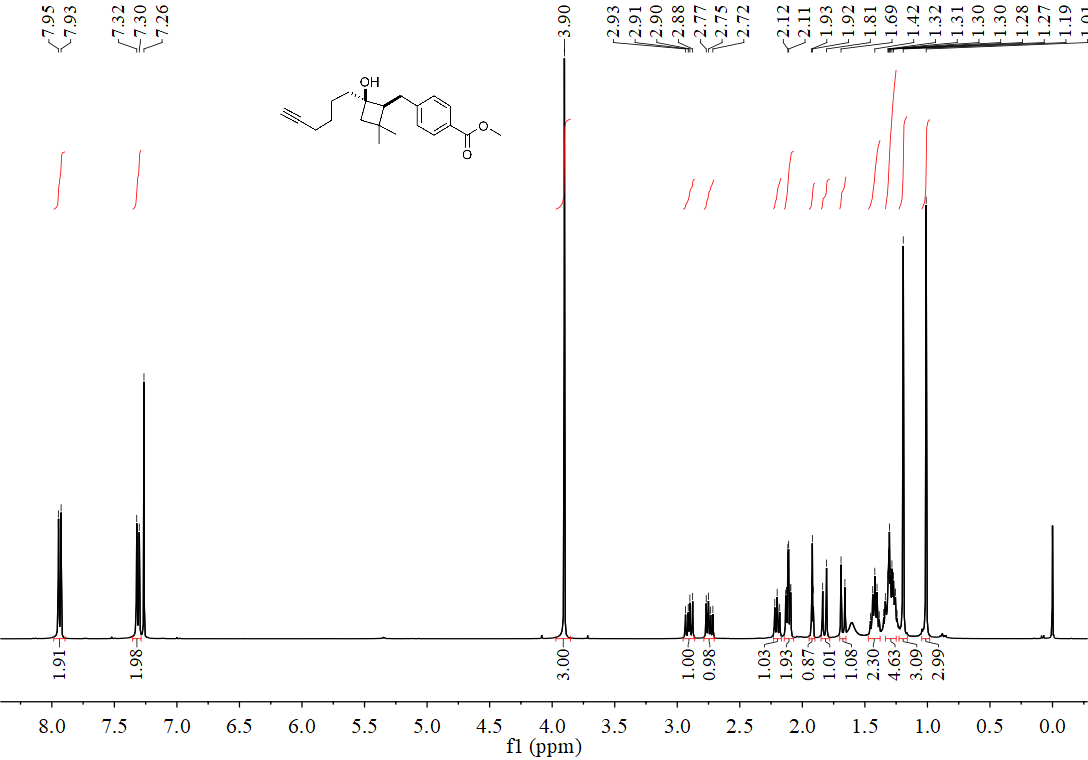
**

**
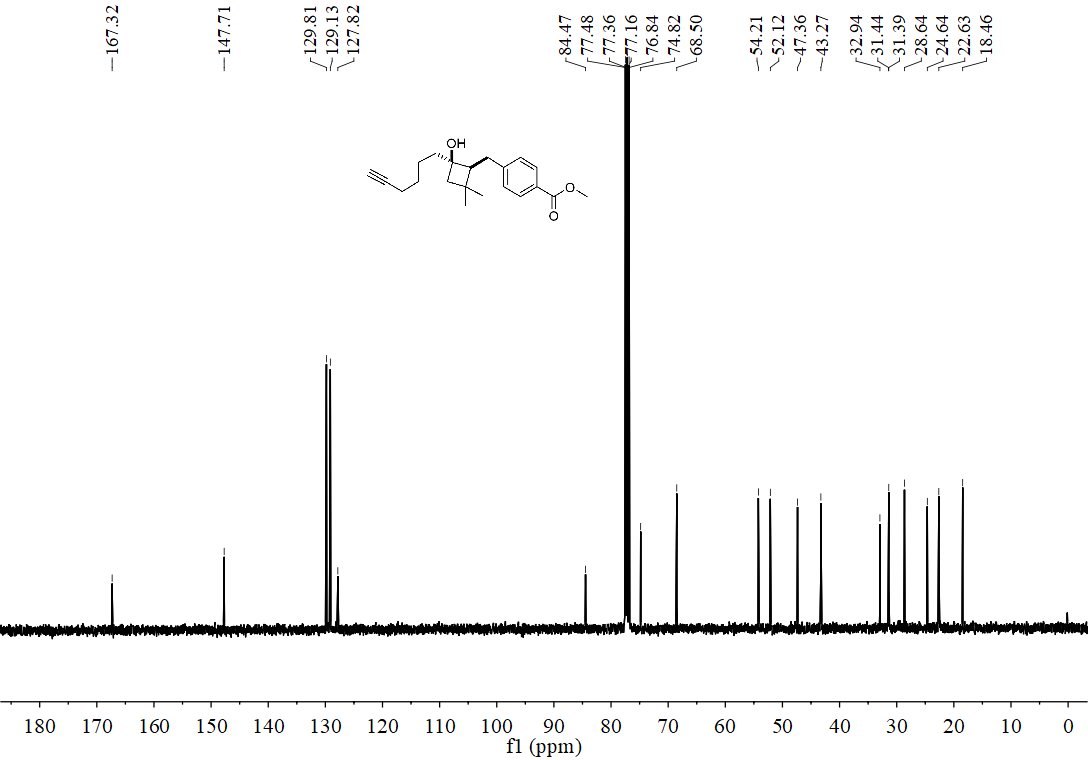
**

**Supplementary Fig. 30.** ^1^H NMR and ^13^C NMR spectrum of **3g_1_**.

**
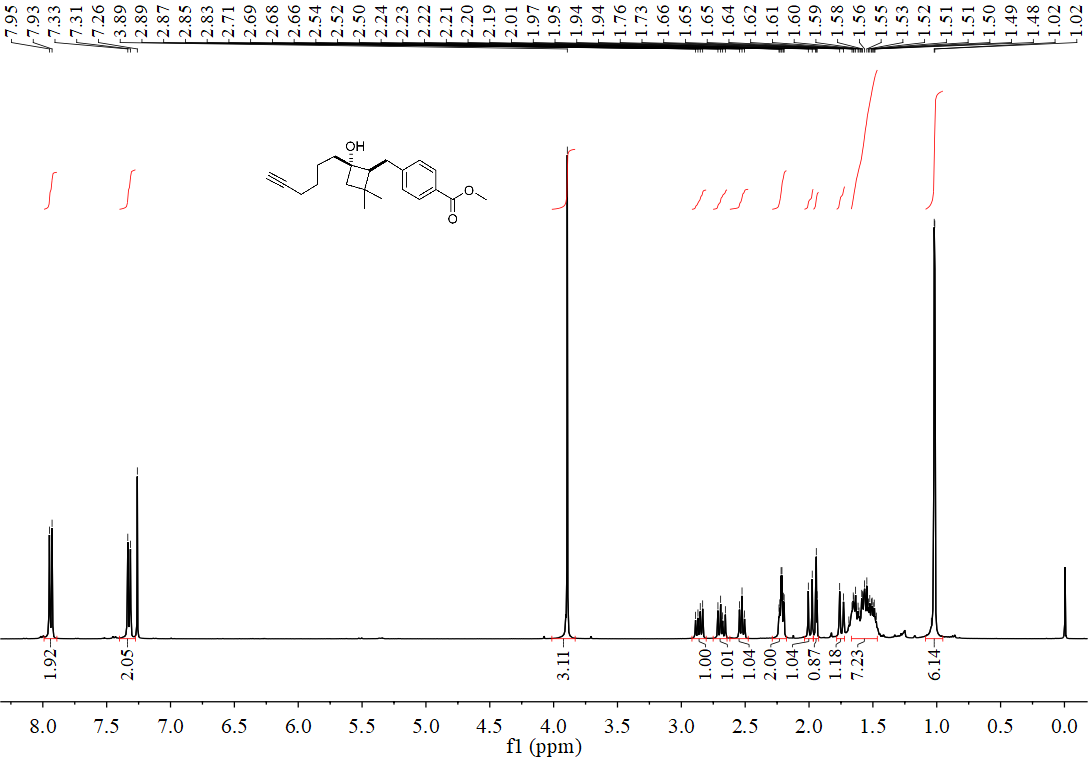
**

**
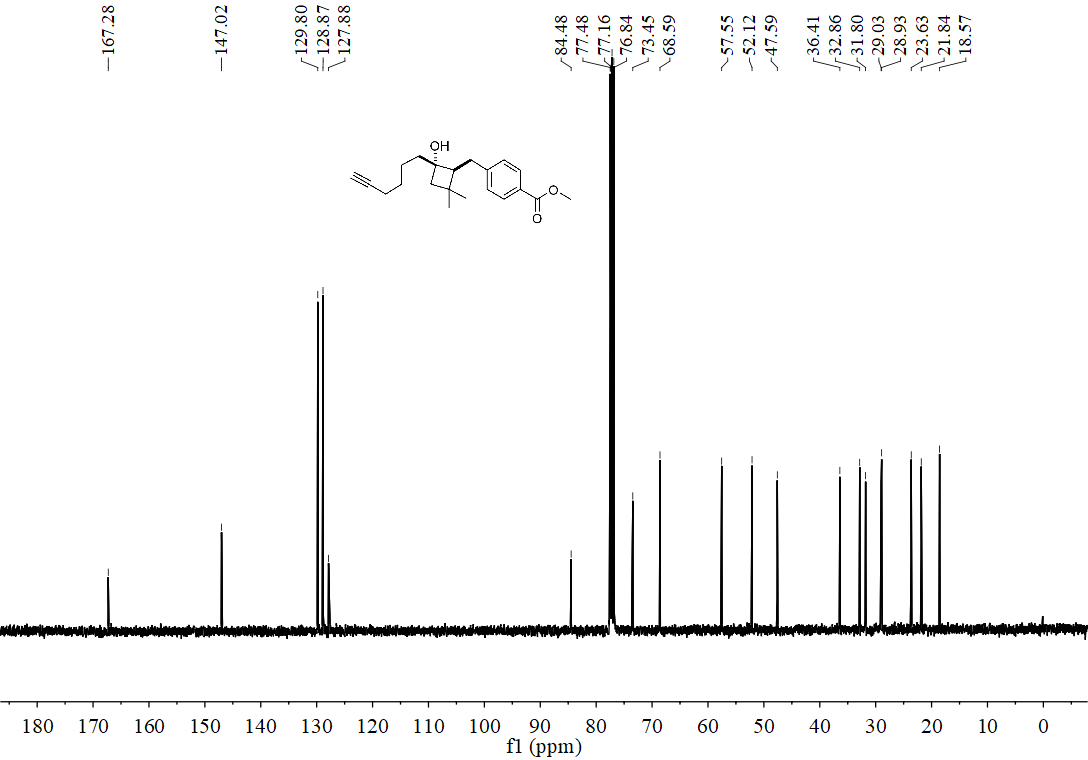
**

**Supplementary Fig. 31.** ^1^H NMR and ^13^C NMR spectrum of **3g_2_**.

**
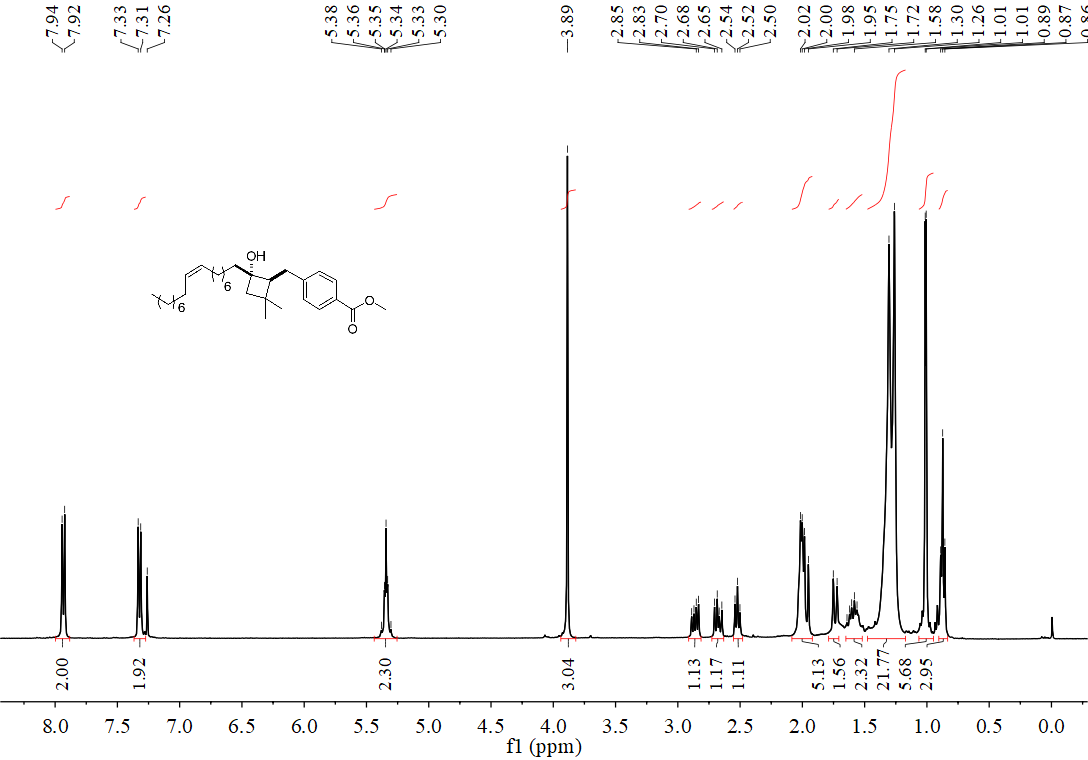
**

**
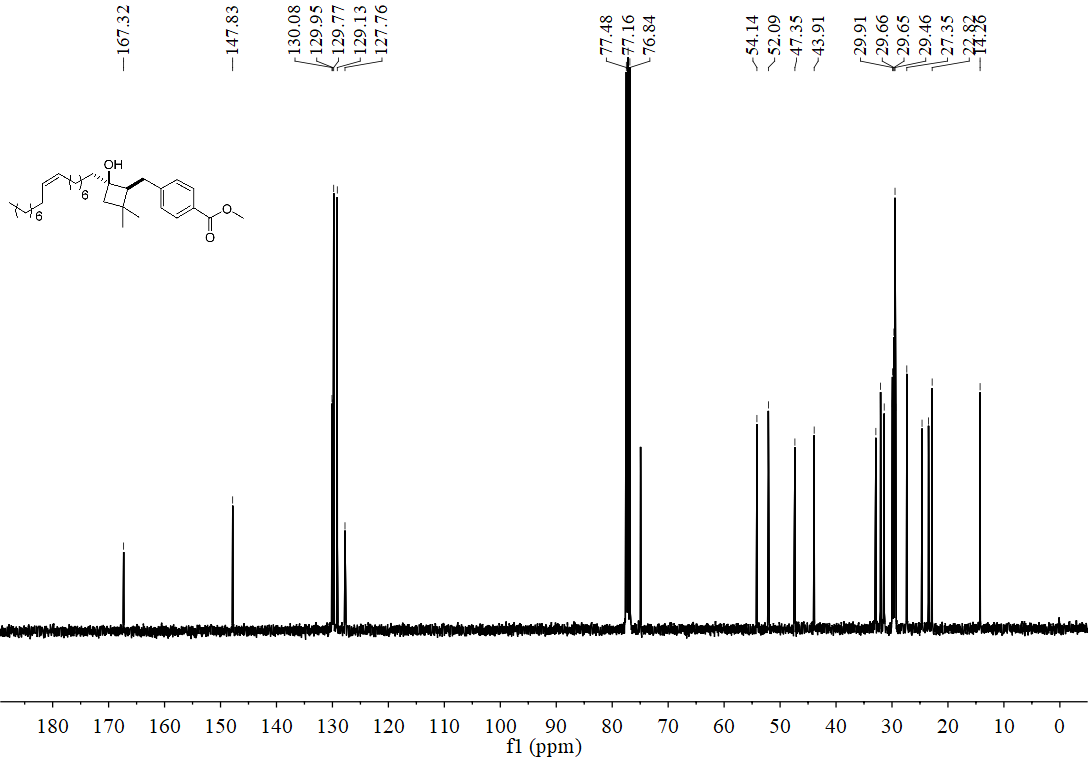
**

**Supplementary Fig. 32.** ^1^H NMR and ^13^C NMR spectrum of **3h_1_**.

**
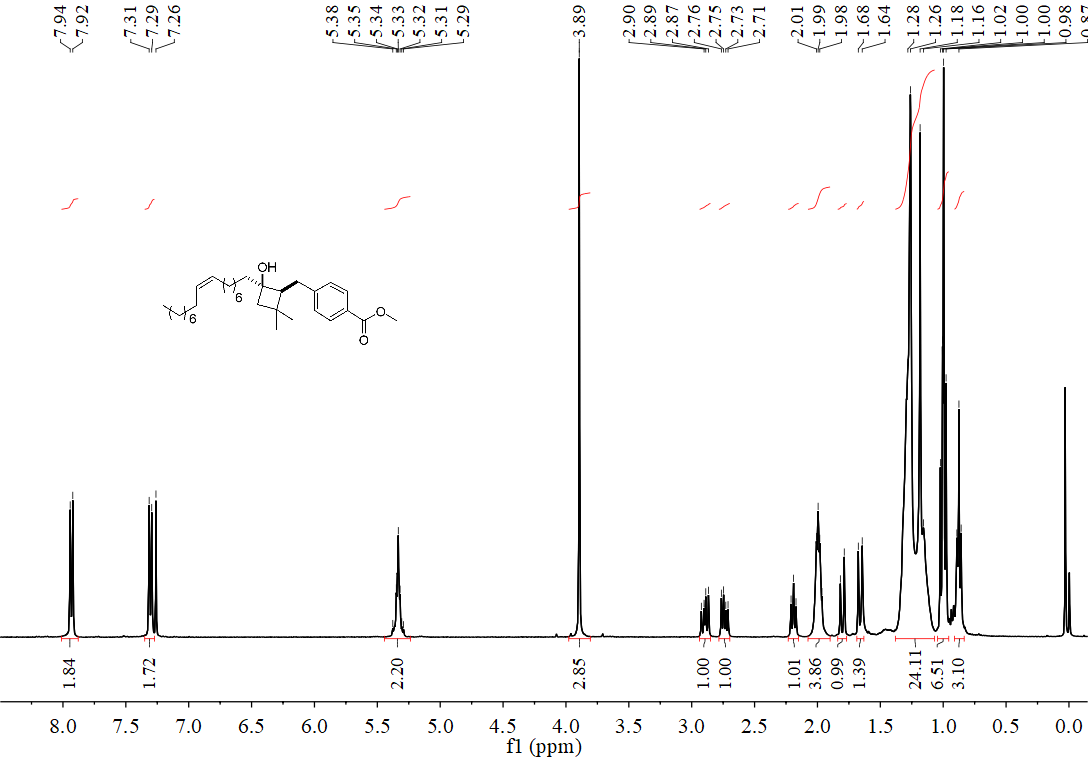
**

**
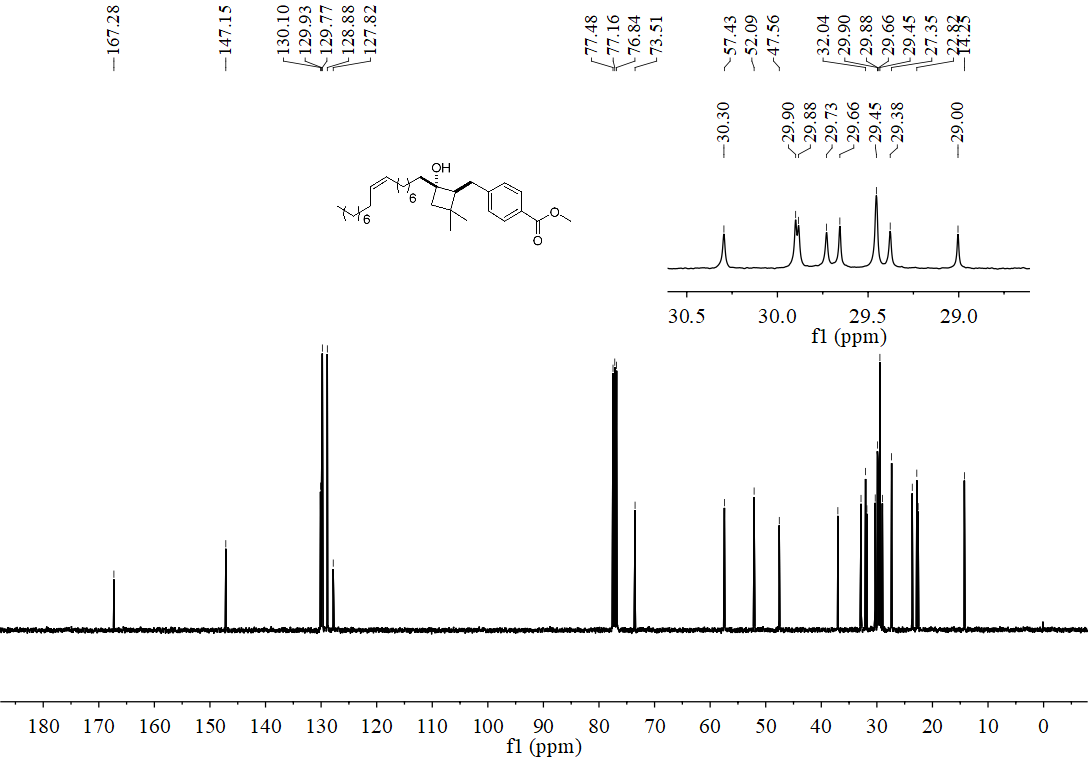
**

**Supplementary Fig. 33.** ^1^H NMR and ^13^C NMR spectrum of **3h_2_**.

**
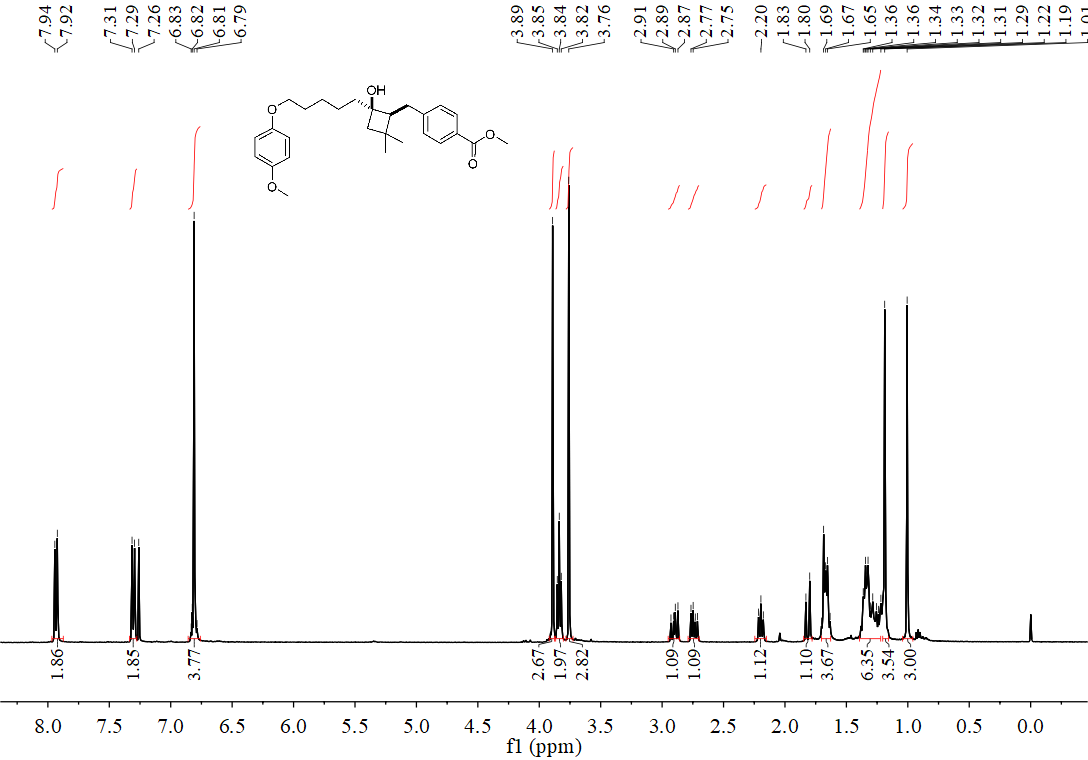
**

**
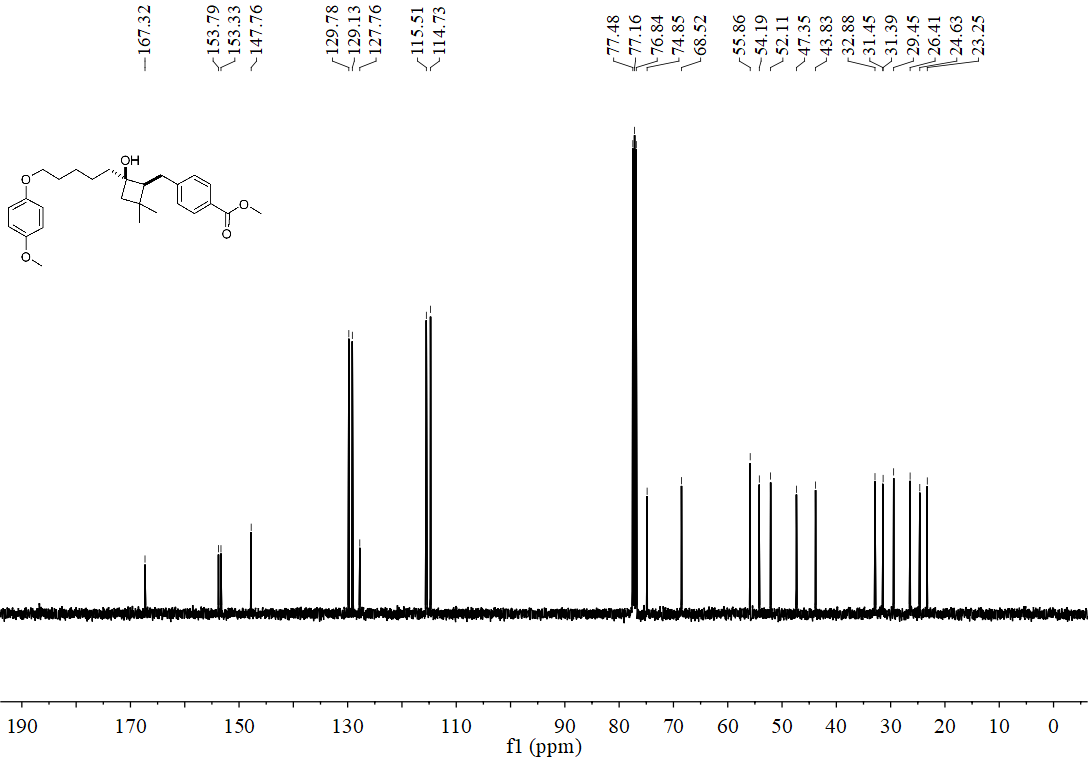
**

**Supplementary Fig. 34.** ^1^H NMR and ^13^C NMR spectrum of **3i_1_**.

**
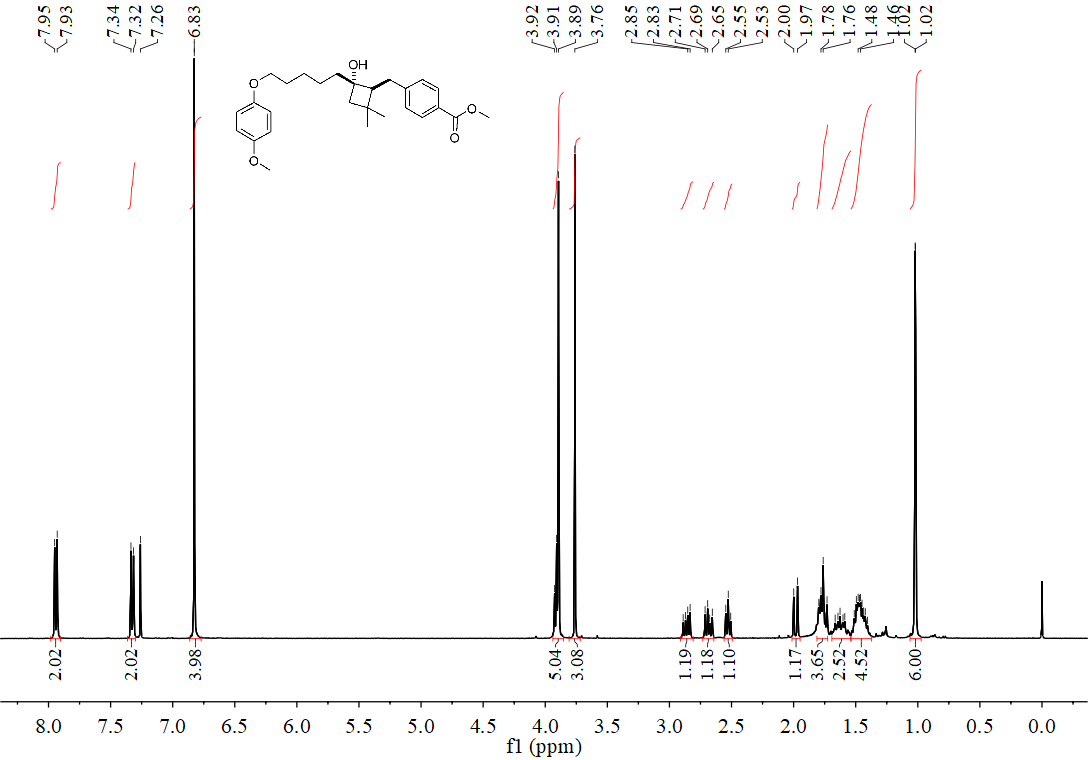
**


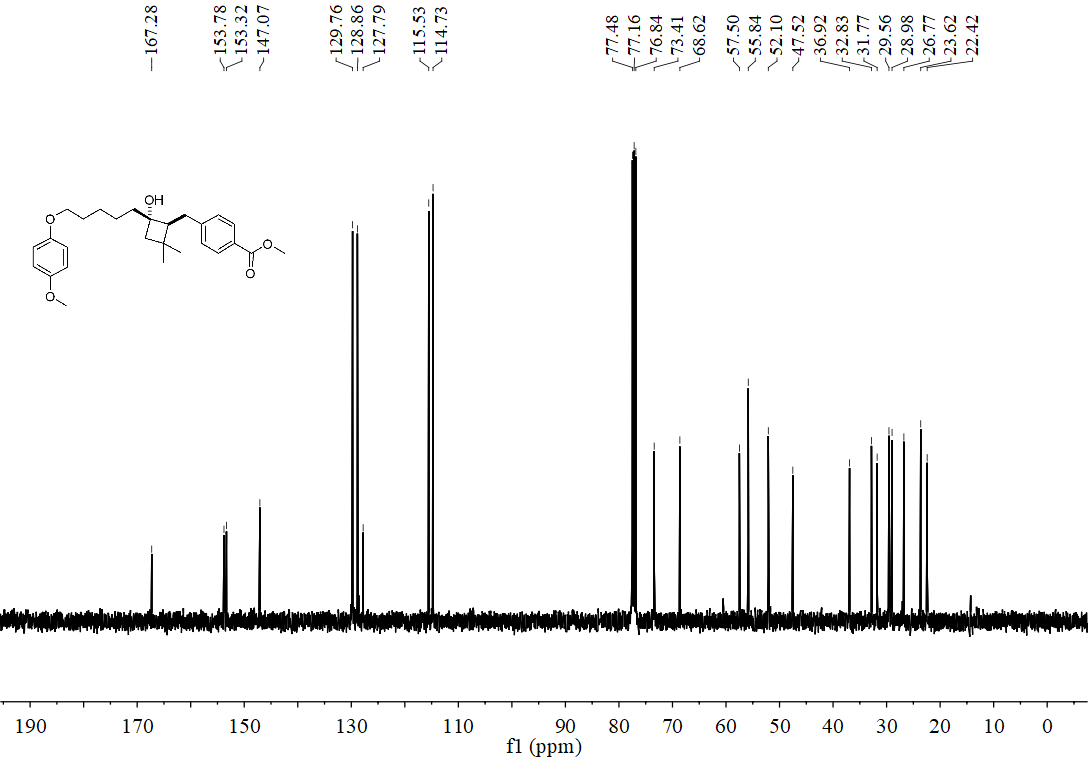


**Supplementary Fig. 35.** ^1^H NMR and ^13^C NMR spectrum of **3i_2_**.

**
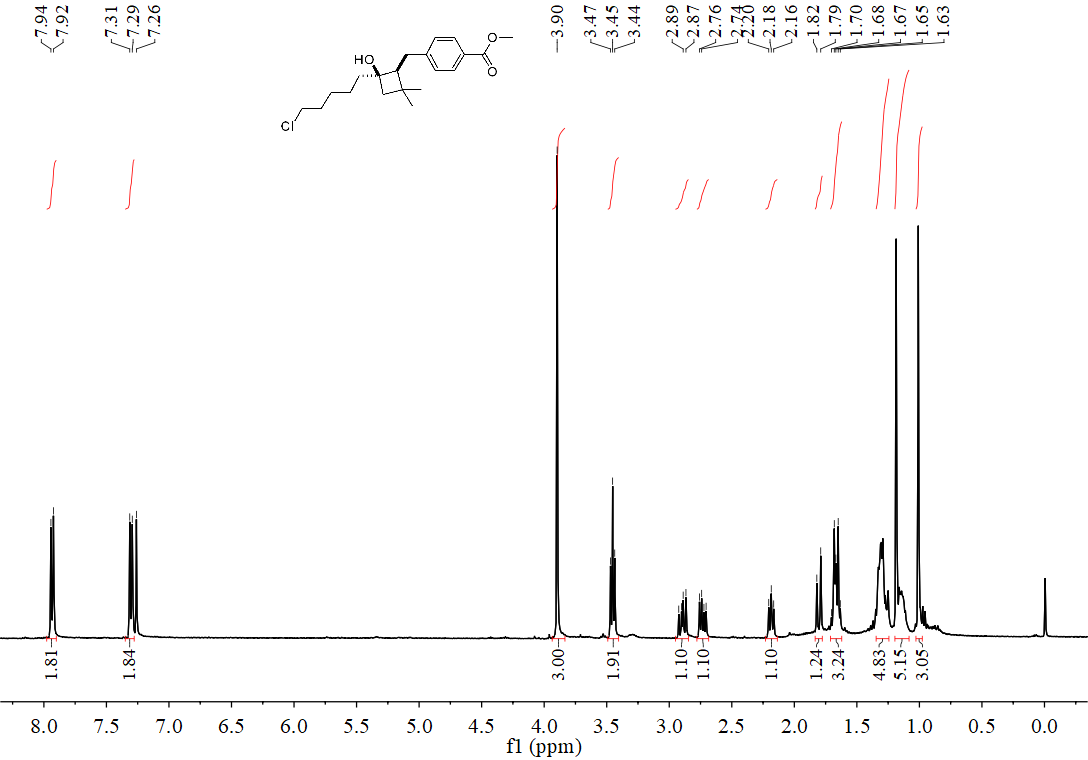
**

**
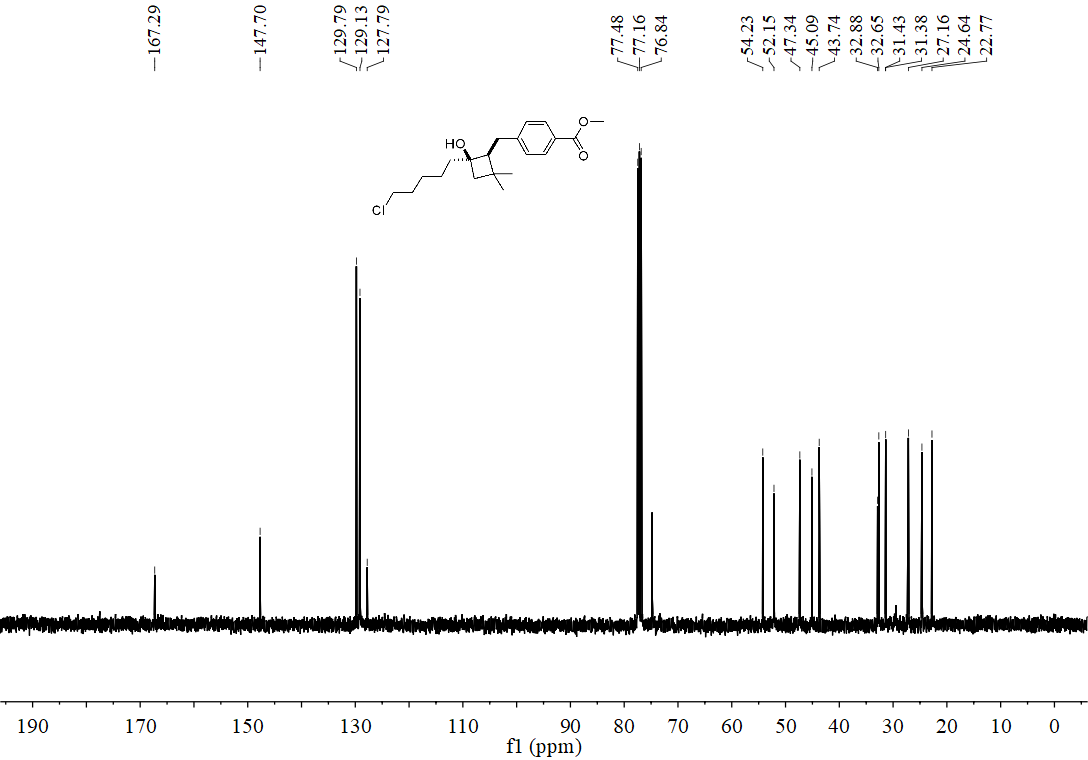
**

**Supplementary Fig. 36.** ^1^H NMR and ^13^C NMR spectrum of **3j_1_**.

**
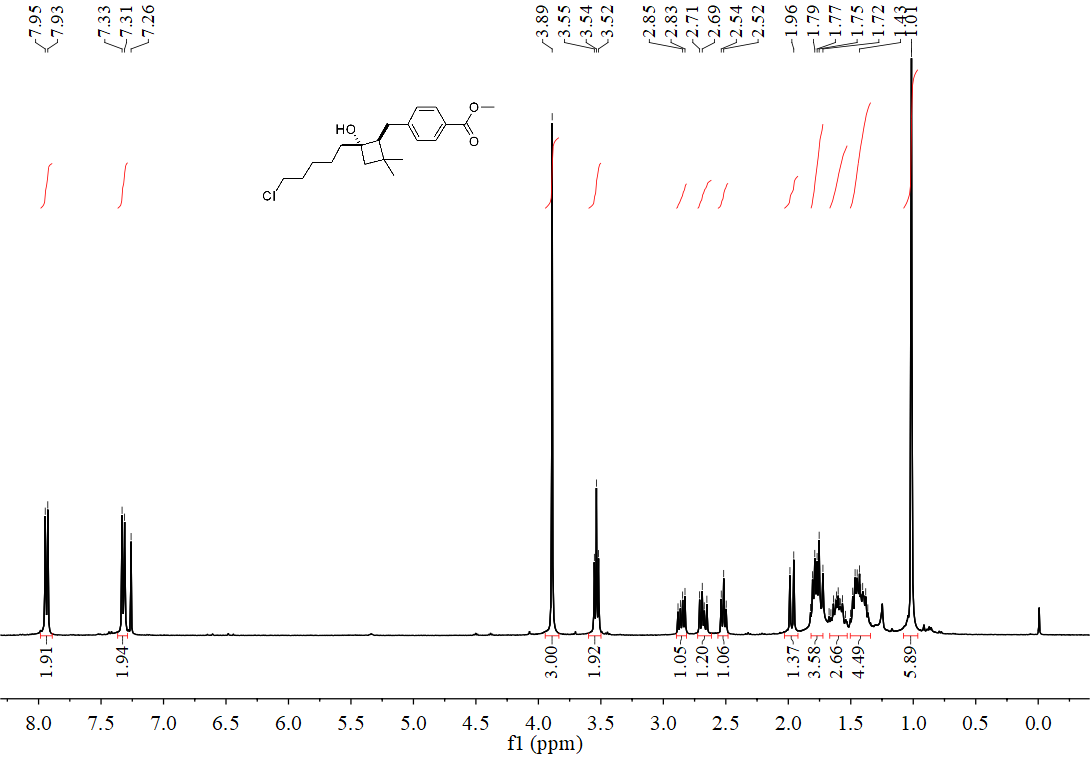
**

**
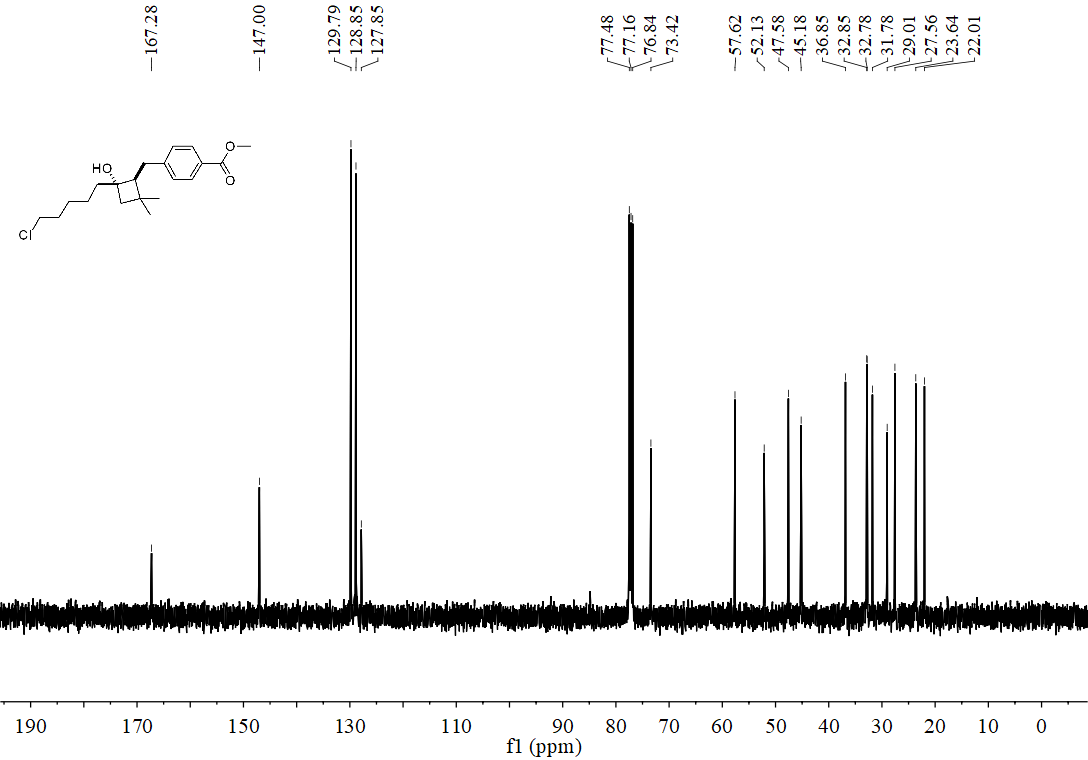
**

**Supplementary Fig. 37.** ^1^H NMR and ^13^C NMR spectrum of **3j_2_**.

**
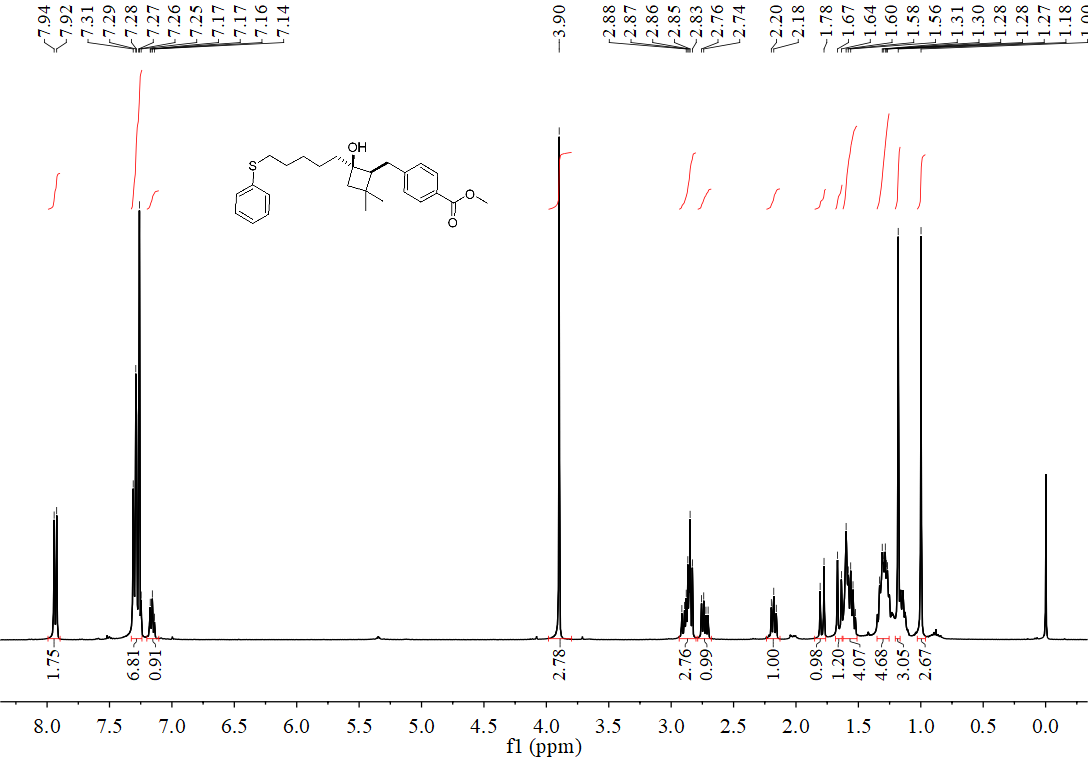
**

**
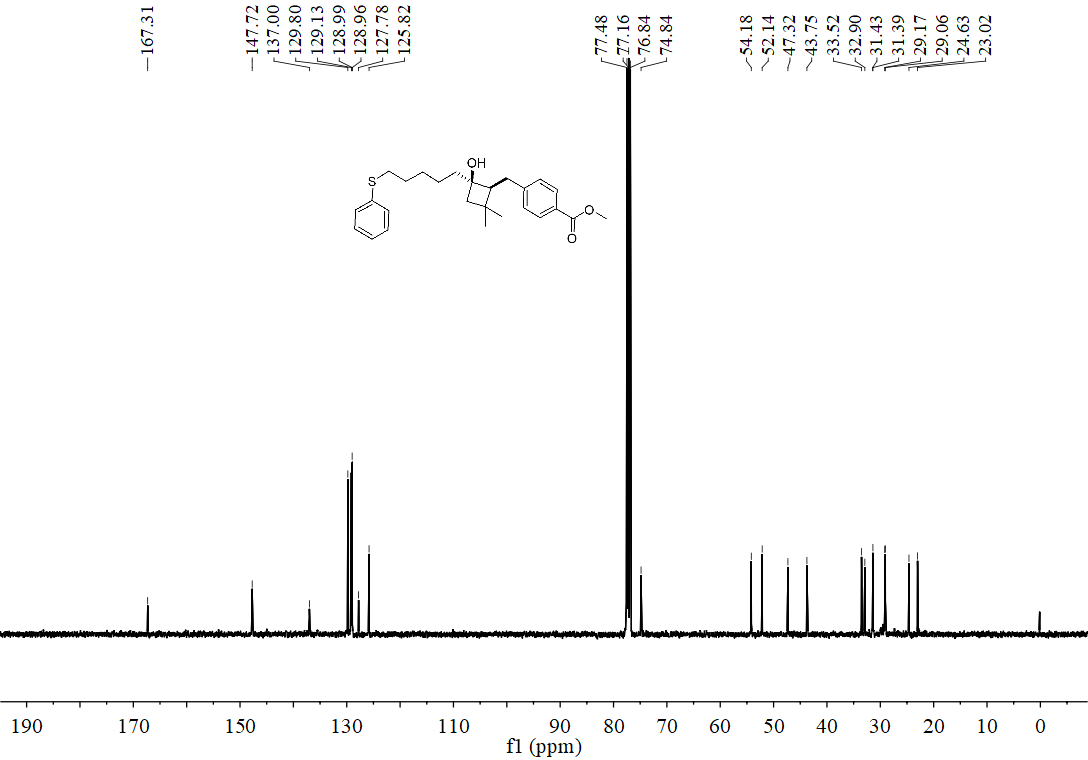
**

**Supplementary Fig. 38.** ^1^H NMR and ^13^C NMR spectrum of **3k_1_**.

**
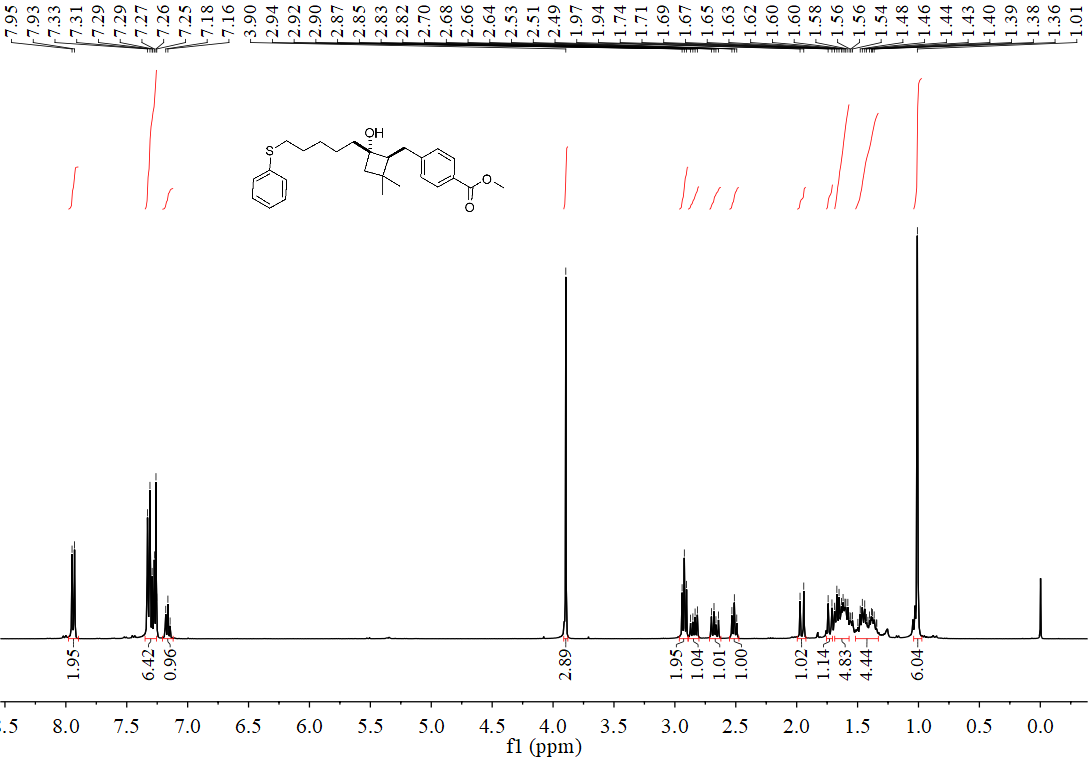
**

**
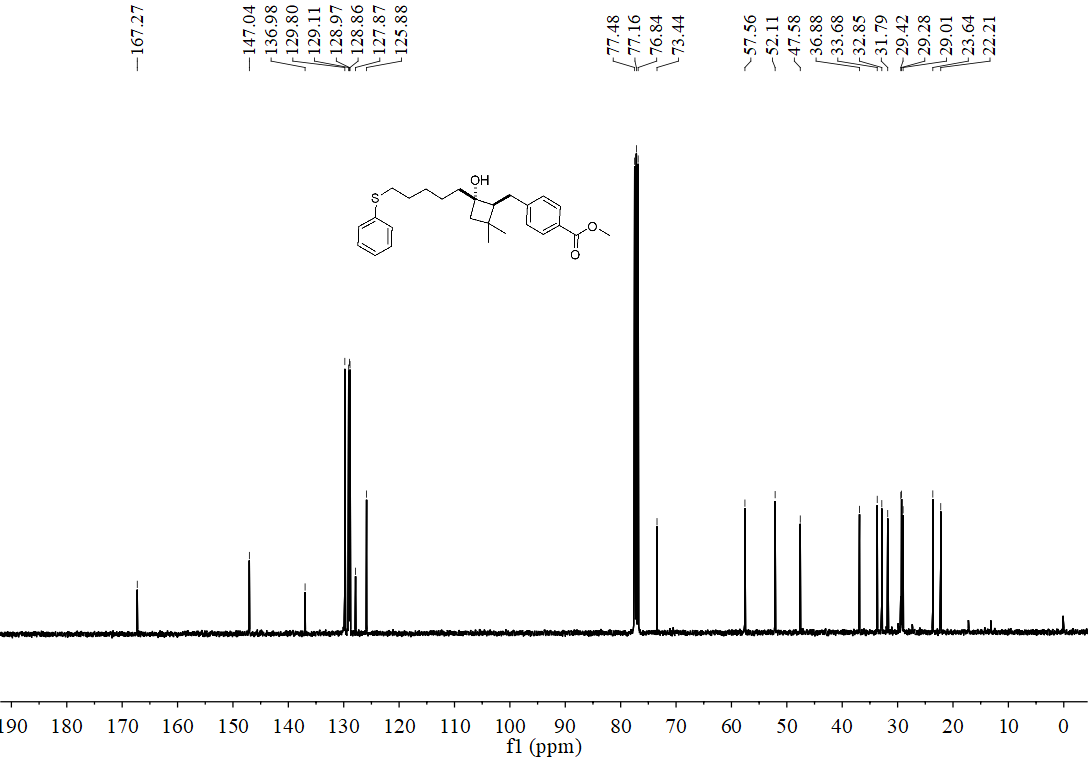
**

**Supplementary Fig. 39.** ^1^H NMR and ^13^C NMR spectrum of **3k_2_**.

**
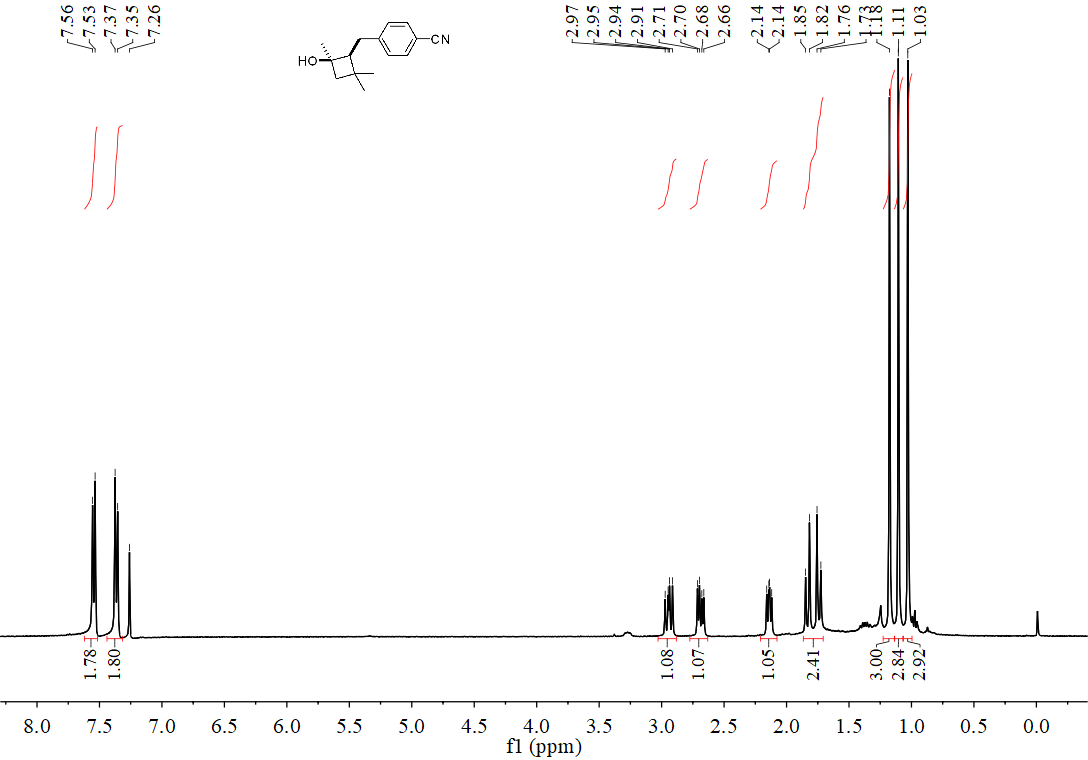
**

**
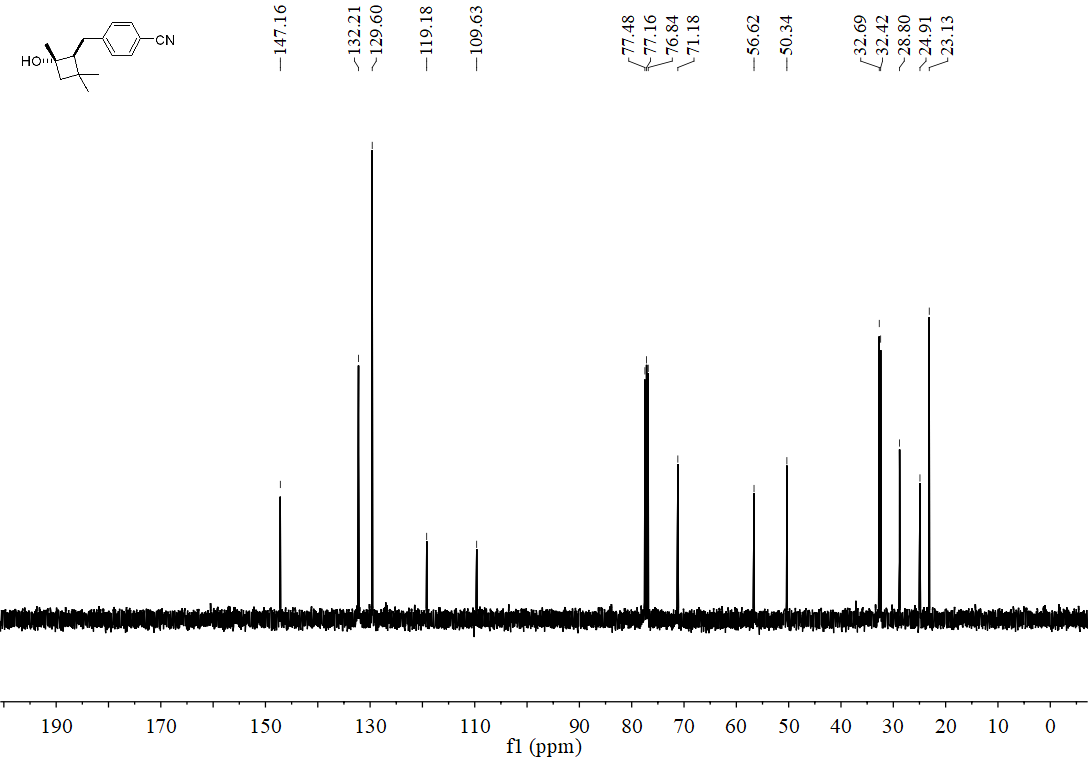
**

**Supplementary Fig. 40.** ^1^H NMR and ^13^C NMR spectrum of **3l_1_**.

**
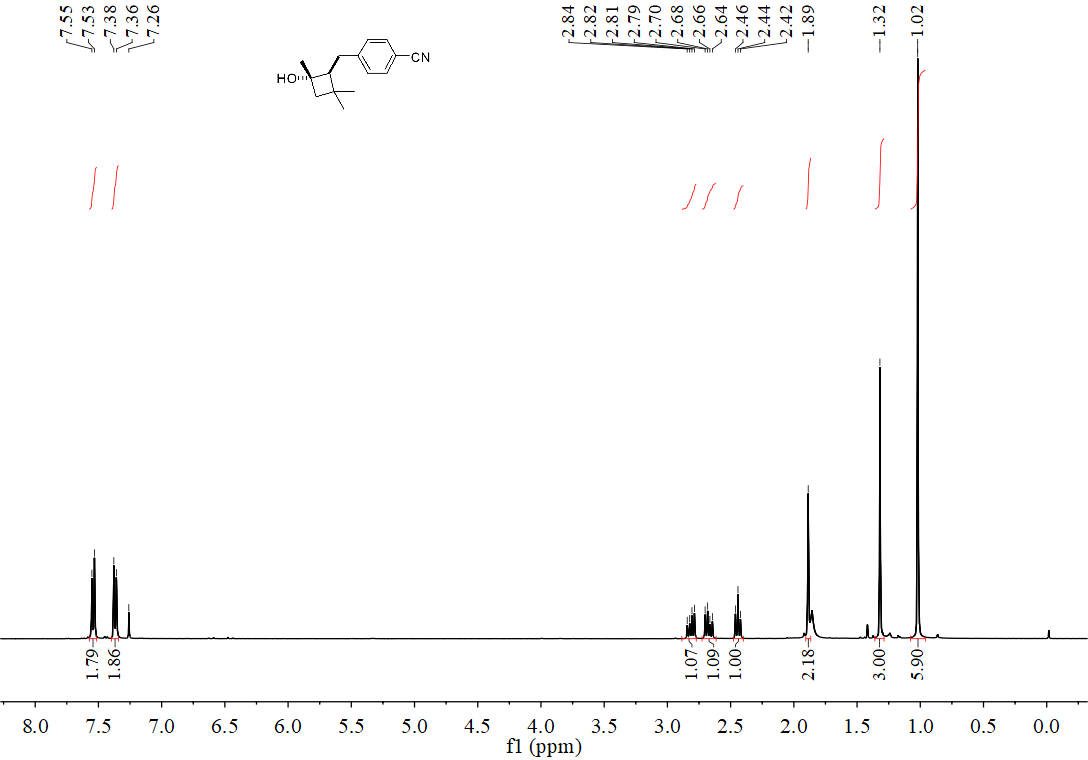
**

**
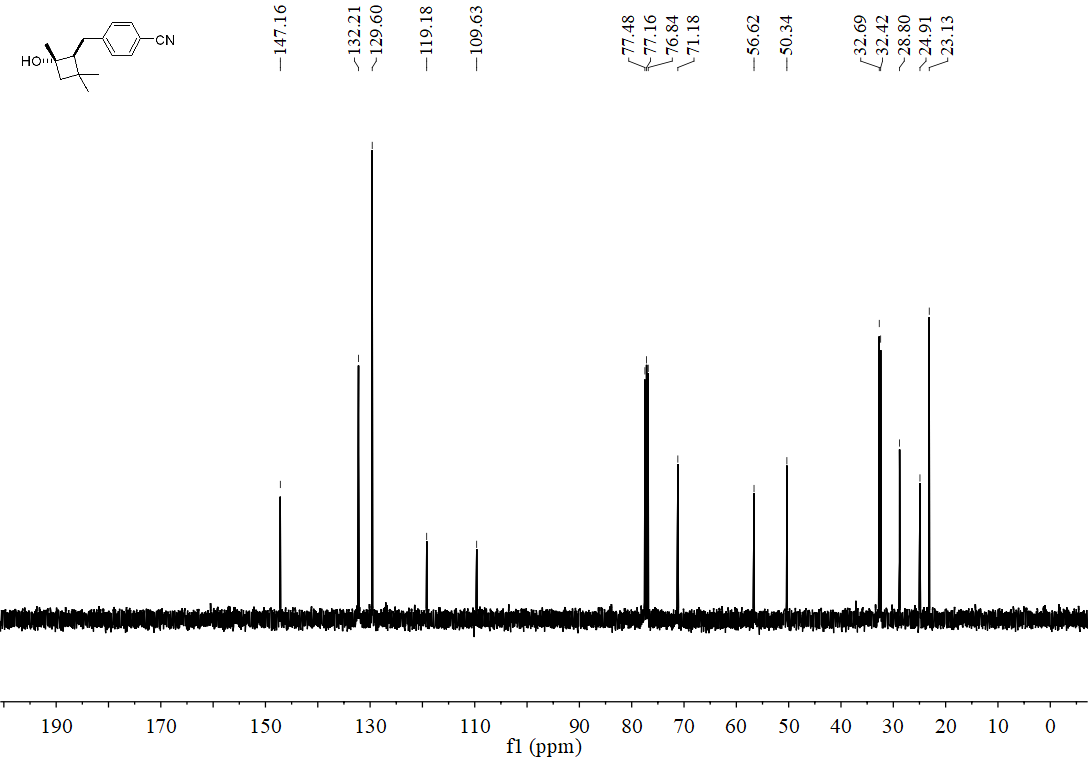
**

**Supplementary Fig. 41.** ^1^H NMR and ^13^C NMR spectrum of **3i_2_**.

**
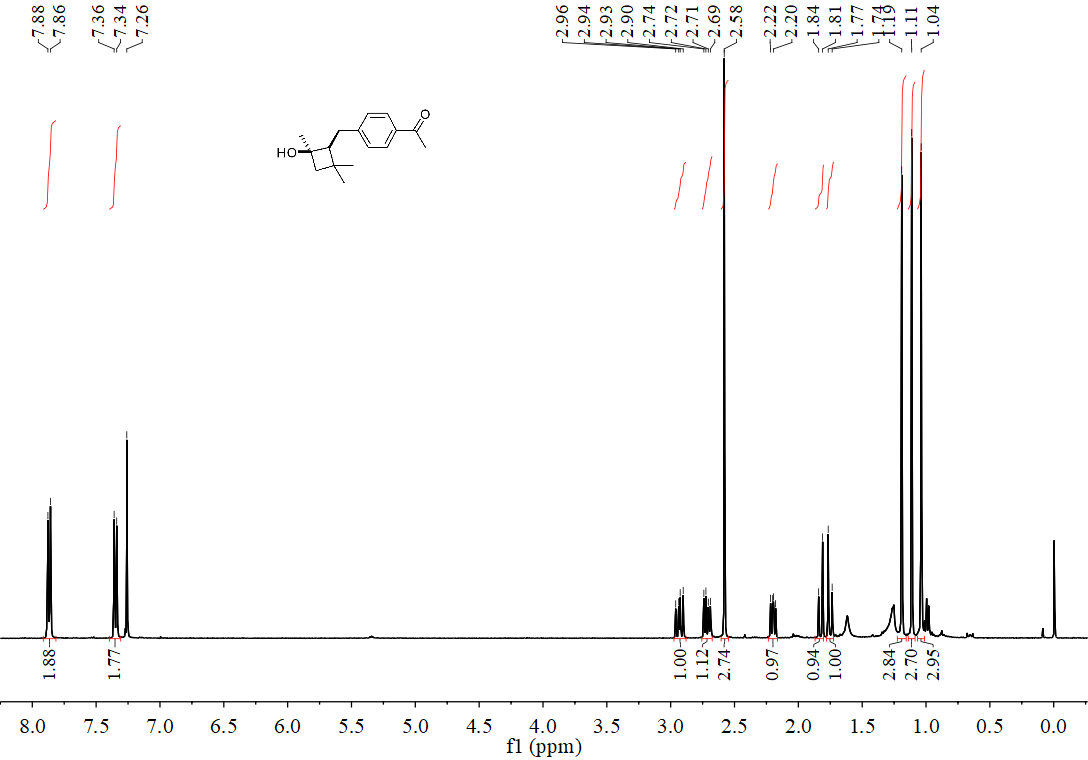
**

**
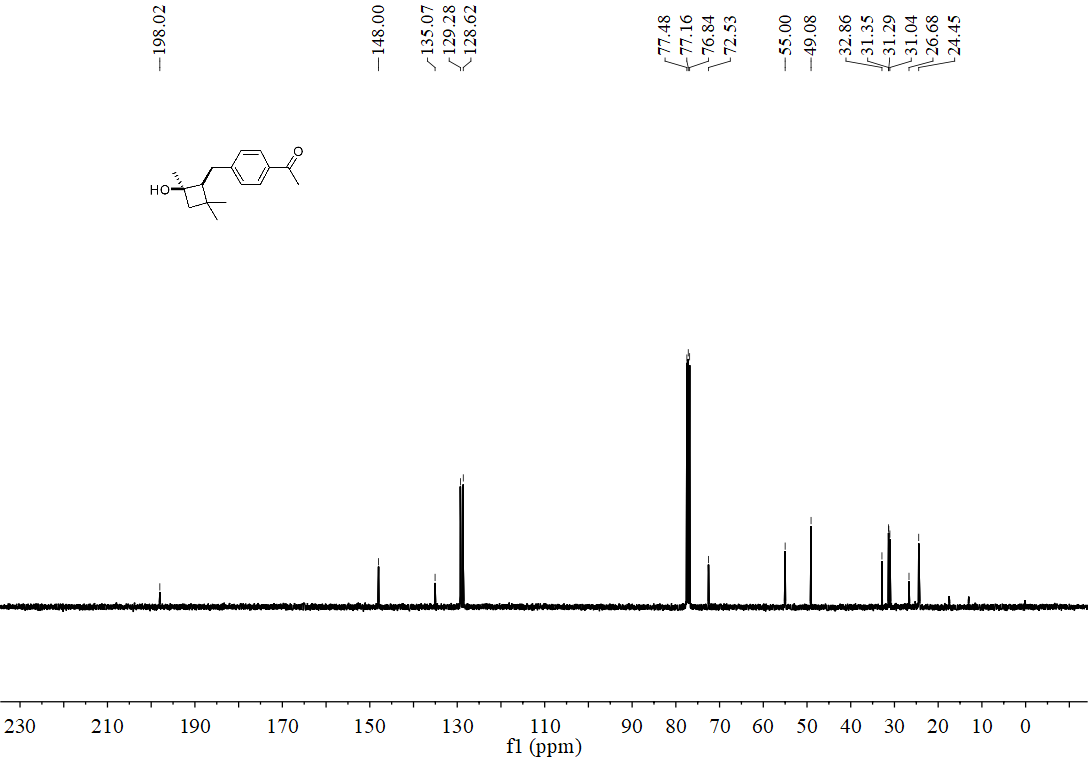
**

**Supplementary Fig. 42.** ^1^H NMR and ^13^C NMR spectrum of **3m_1_**.

**
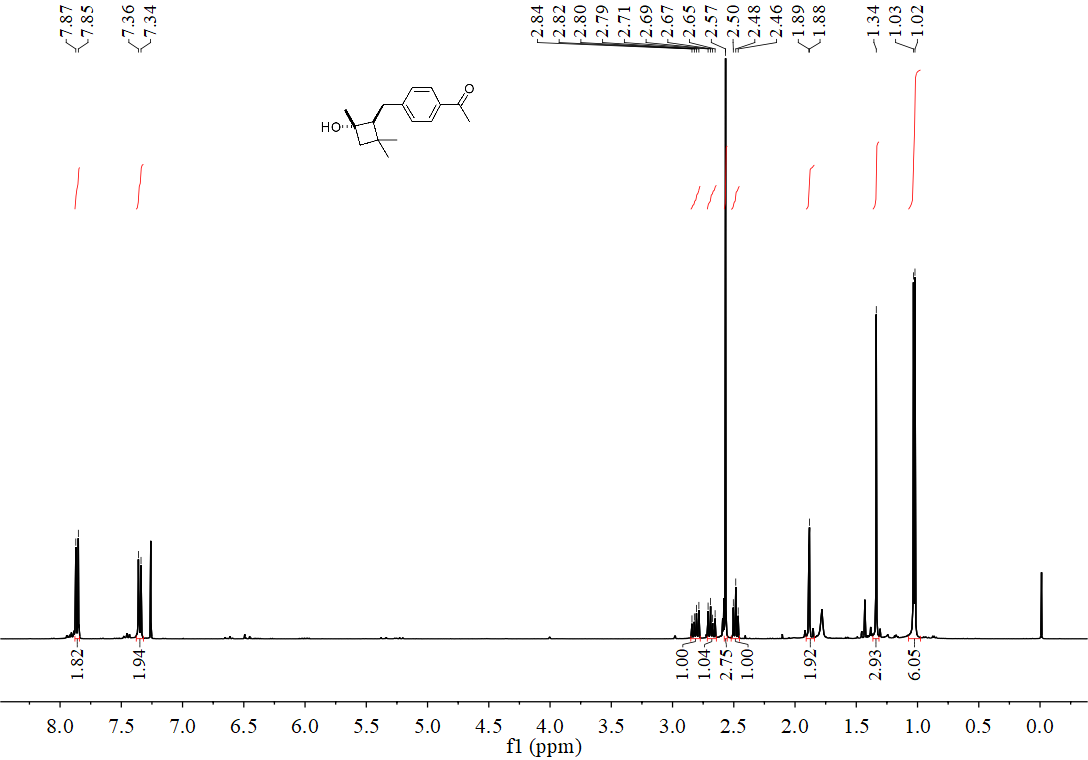
**

**
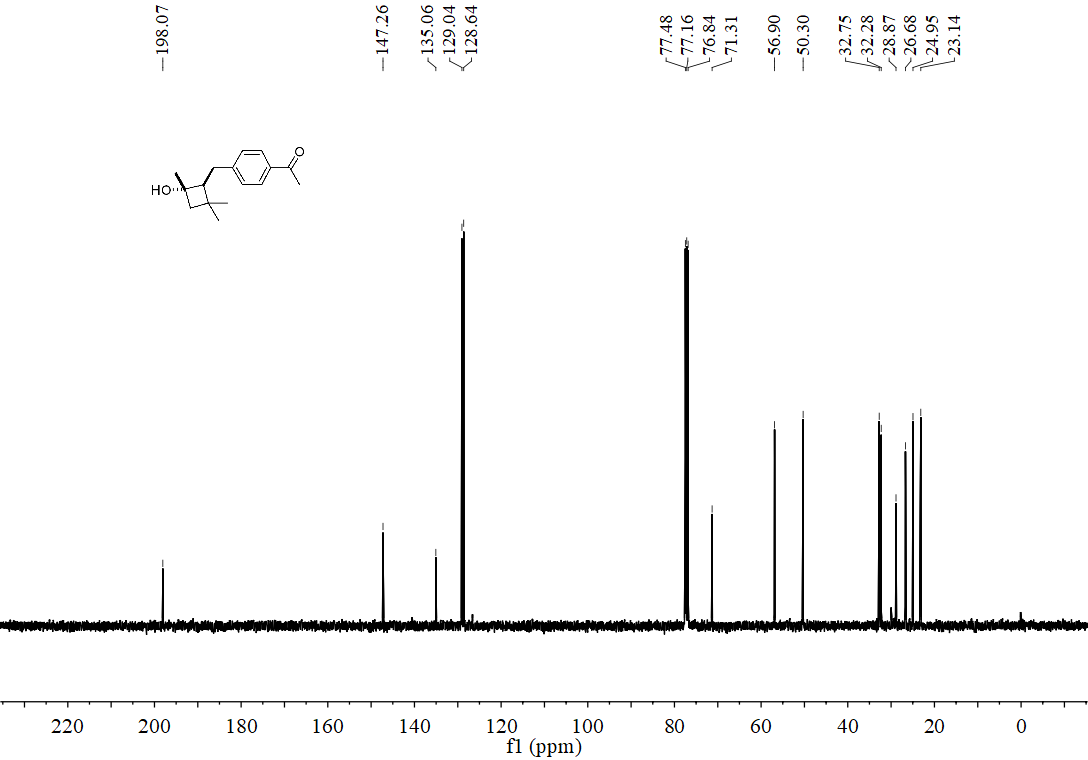
**

**Supplementary Fig. 43.** ^1^H NMR and ^13^C NMR spectrum of **3m_2_**.

**
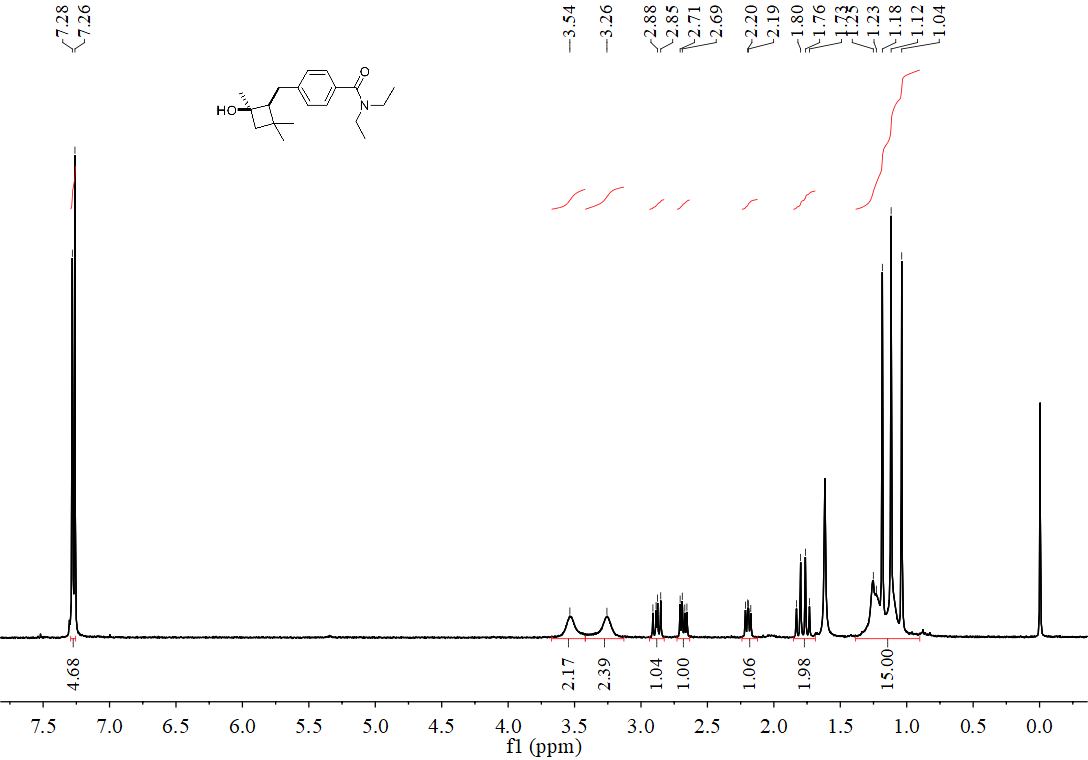
**

**
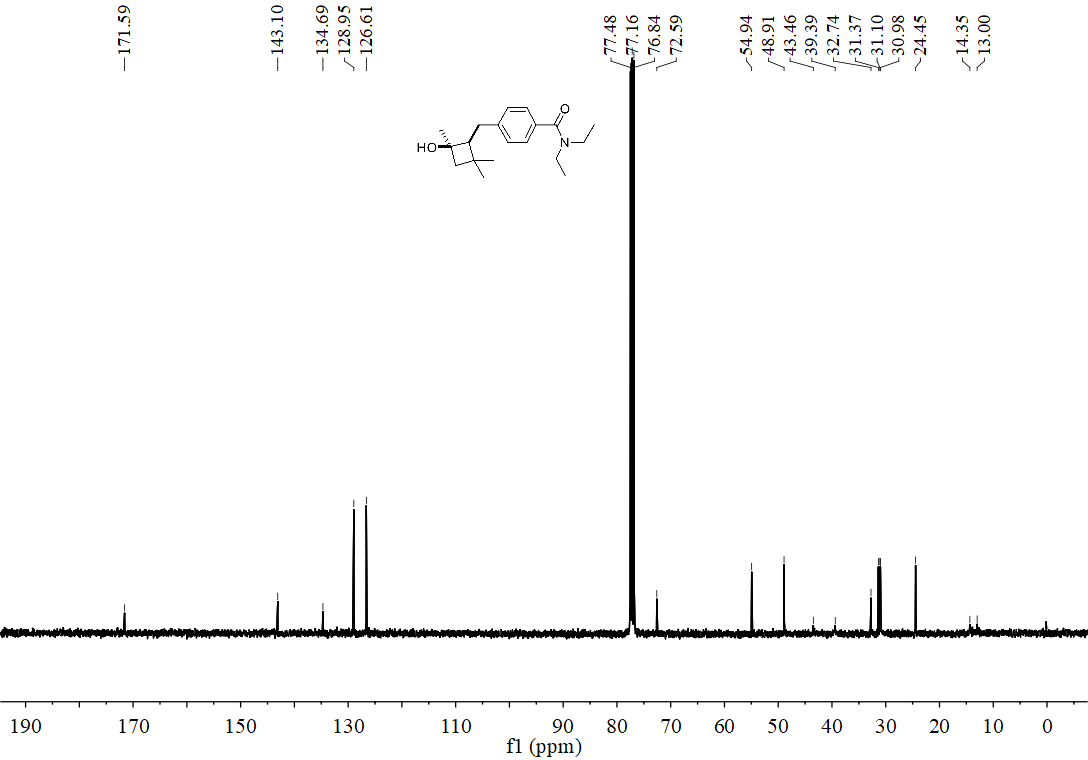
**

**Supplementary Fig. 44.** ^1^H NMR and ^13^C NMR spectrum of **3n_1_**.

**
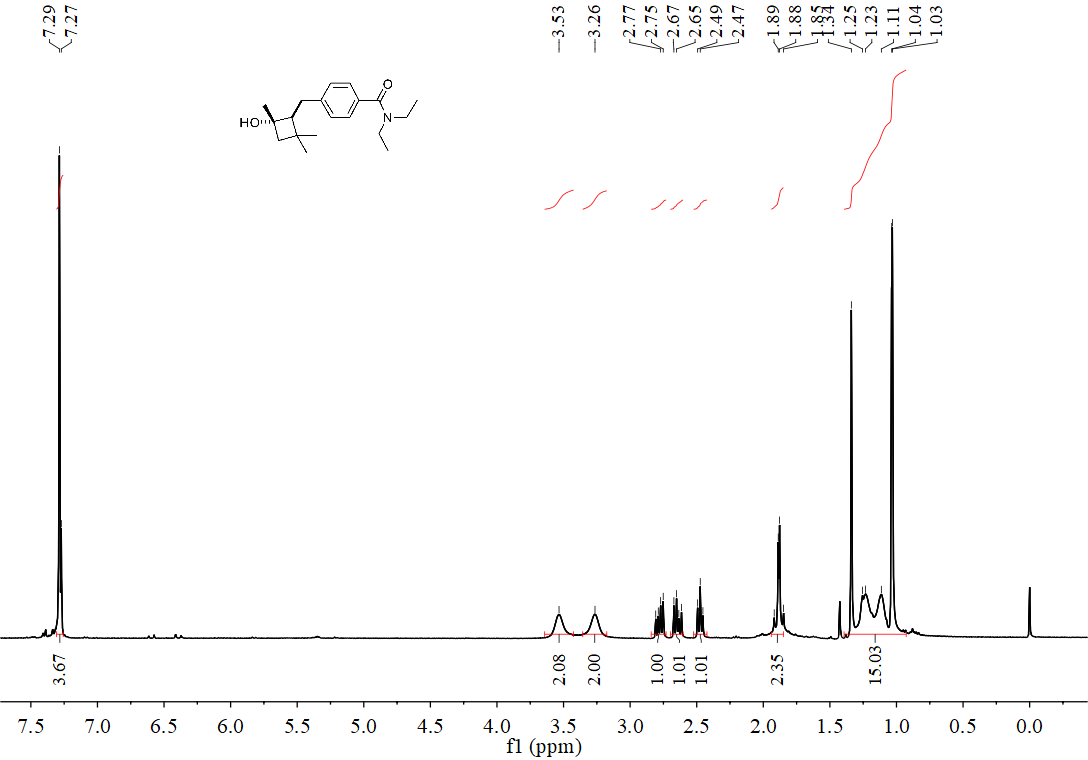
**

**
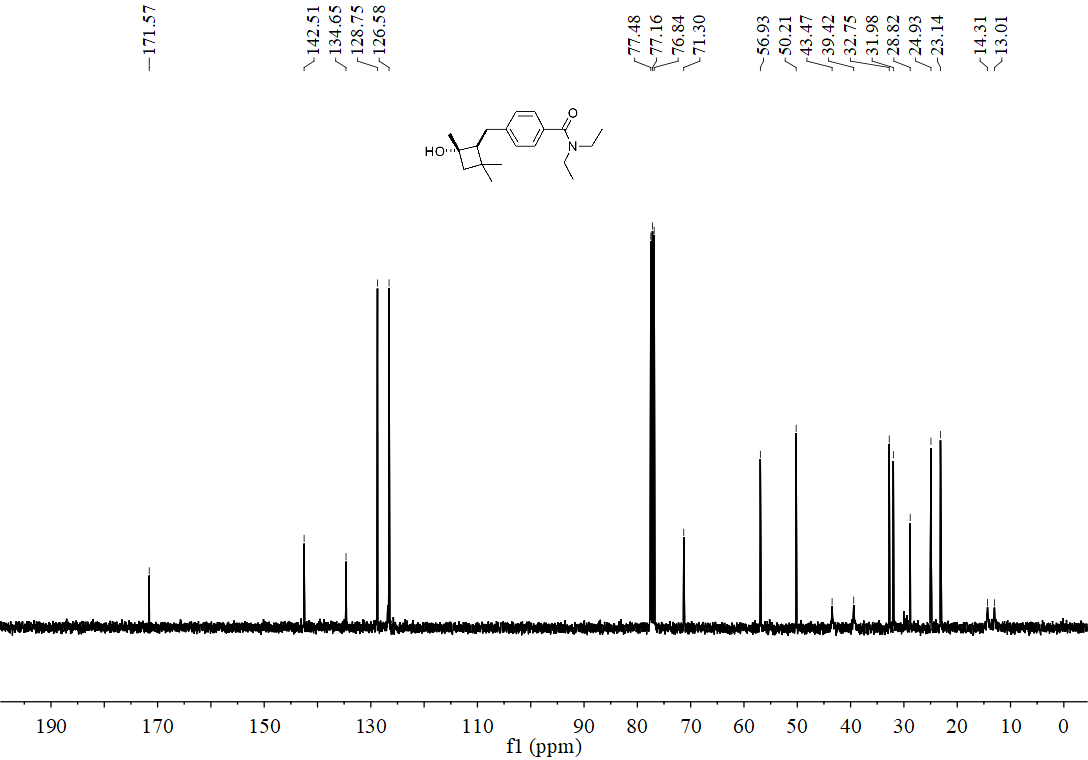
**

**Supplementary Fig. 45.** ^1^H NMR and ^13^C NMR spectrum of **3n_2_**.

**
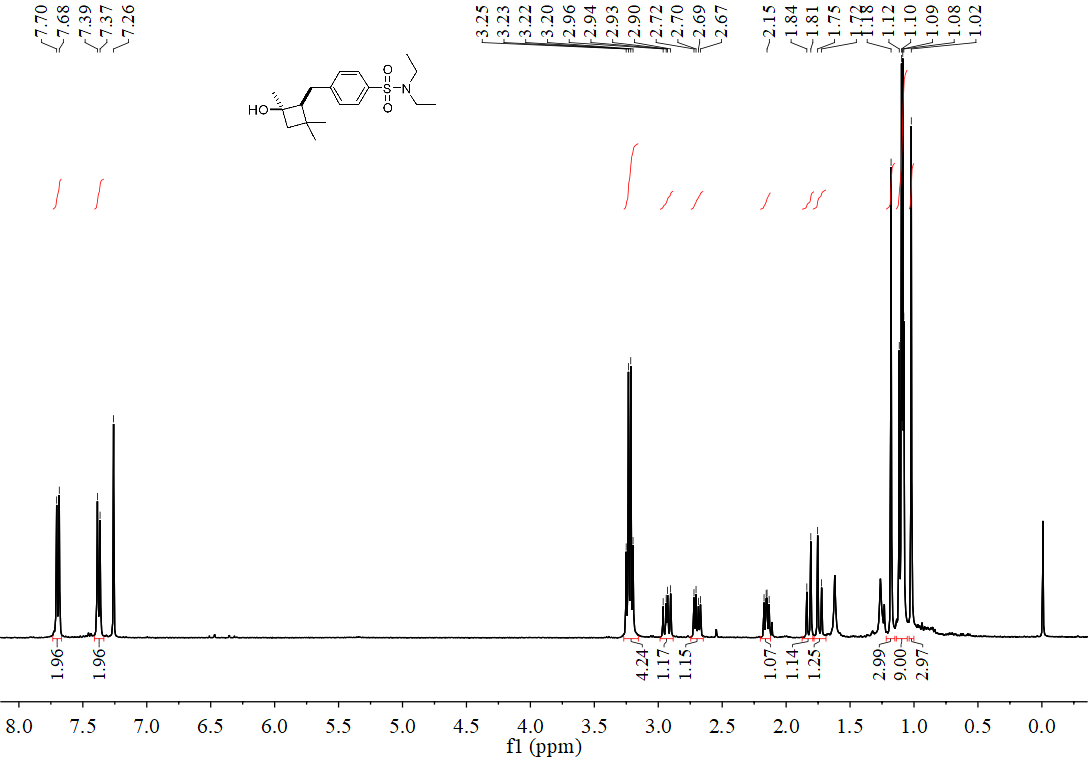
**

**
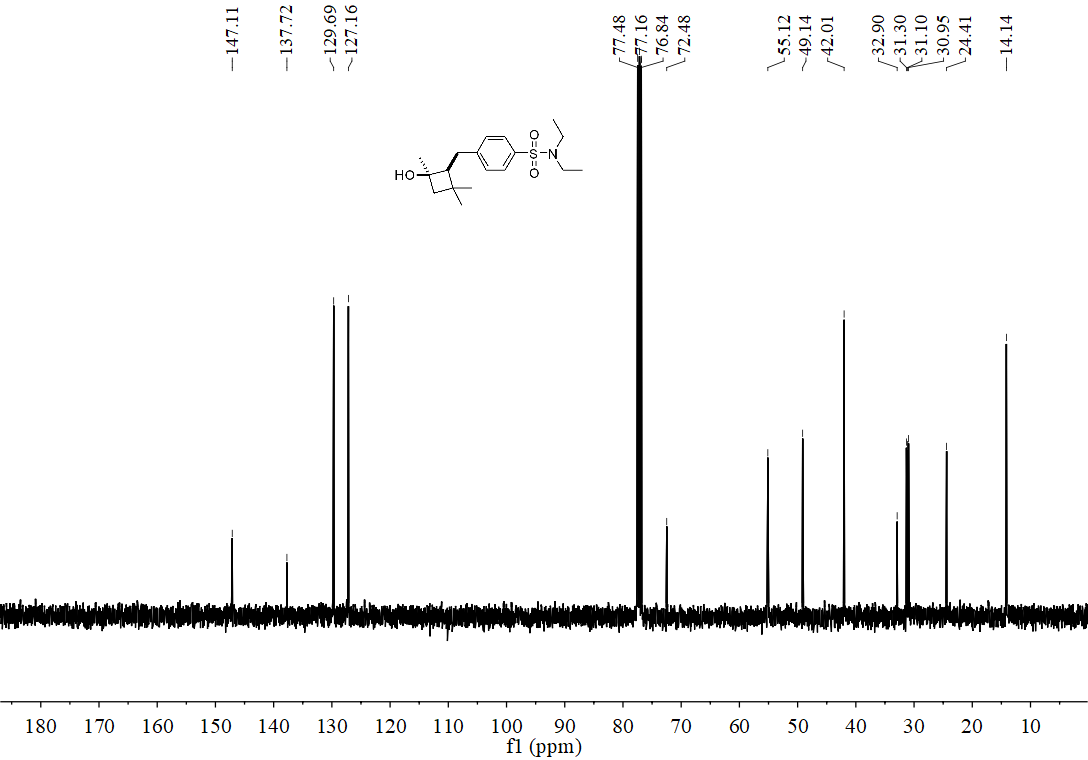
**

**Supplementary Fig. 46.** ^1^H NMR and ^13^C NMR spectrum of **3o_1_**.

**
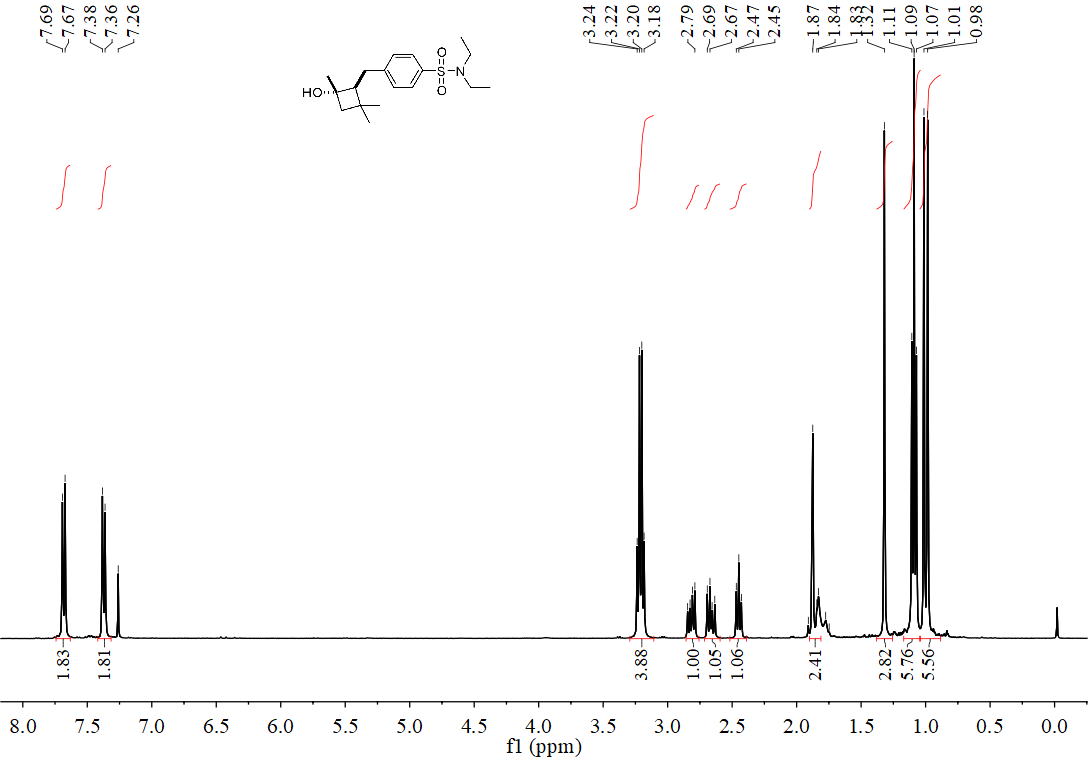
**

**
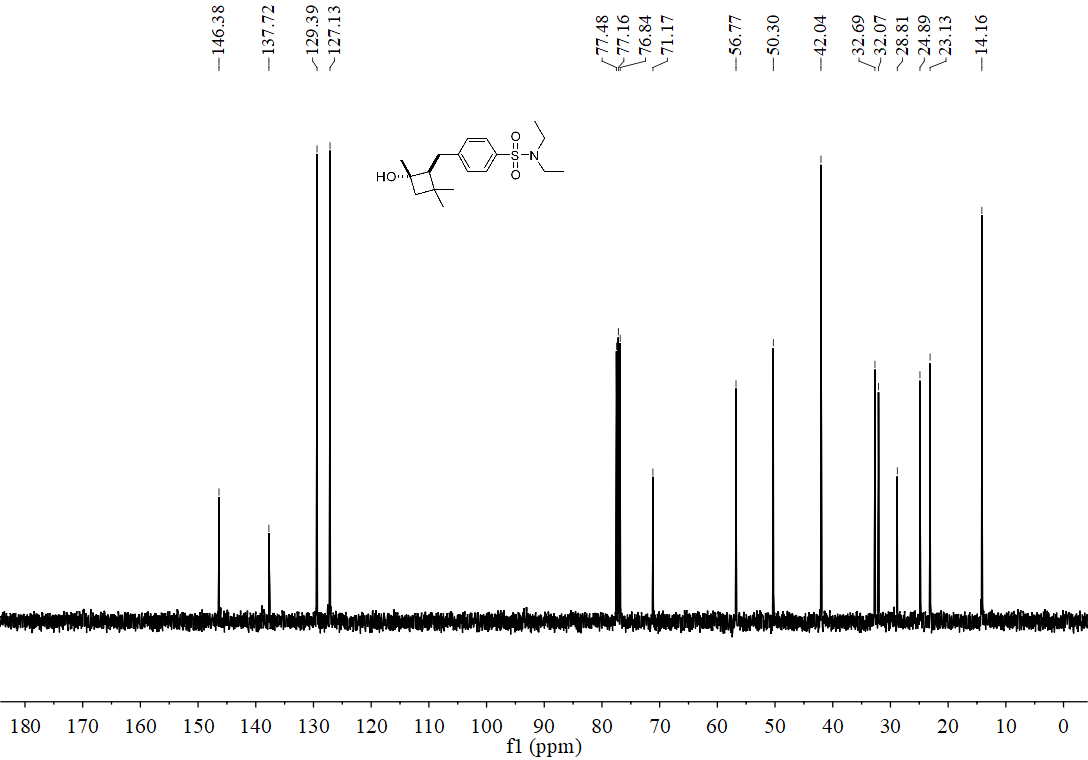
**

**Supplementary Fig. 47.** ^1^H NMR and ^13^C NMR spectrum of **3o_2_**.

**_
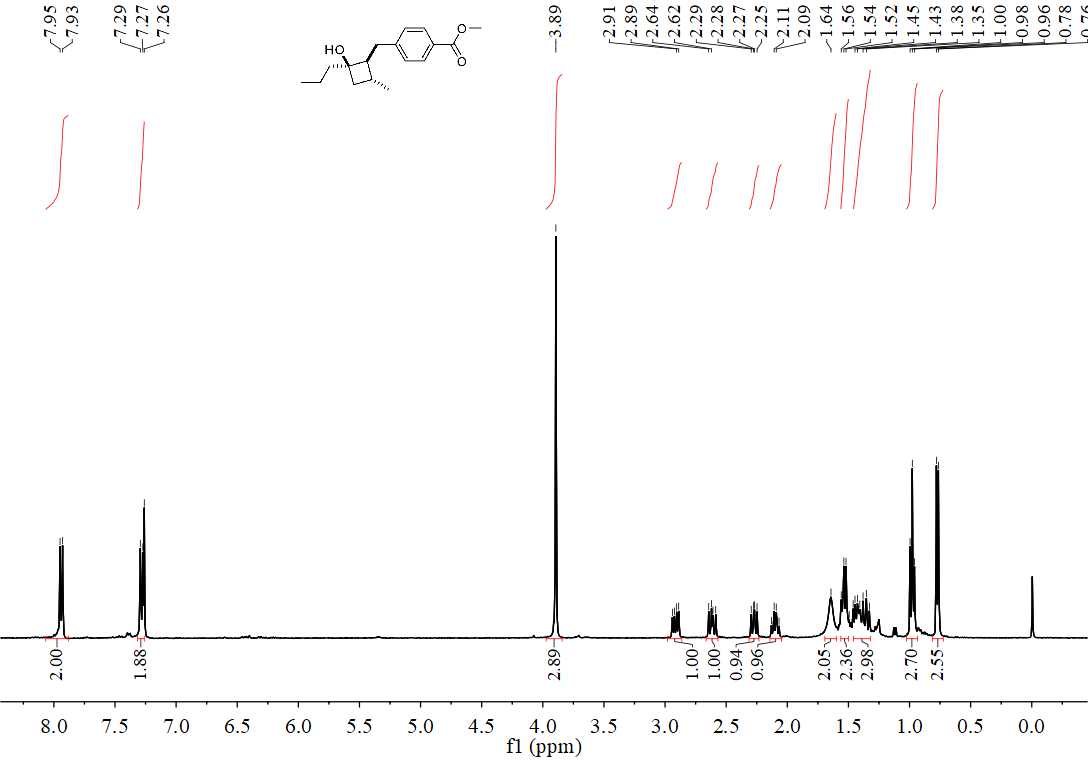
_**

**_
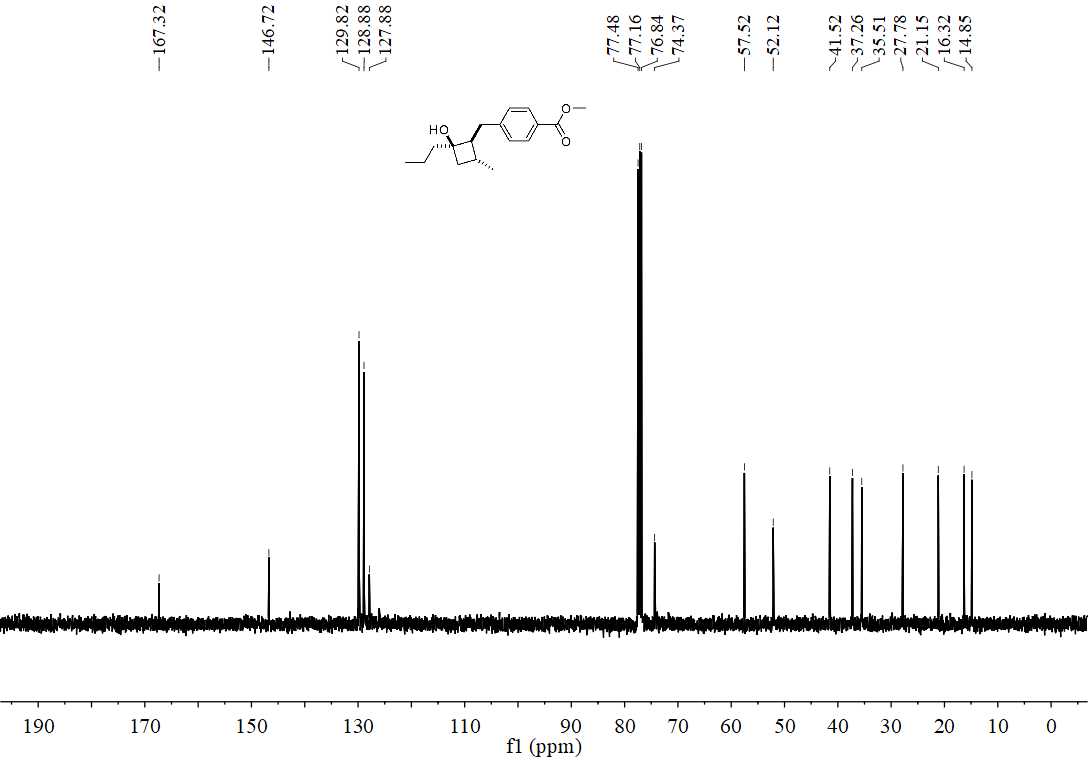
_**

**Supplementary Fig. 48.** ^1^H NMR and ^13^C NMR spectrum of **3p**.

**
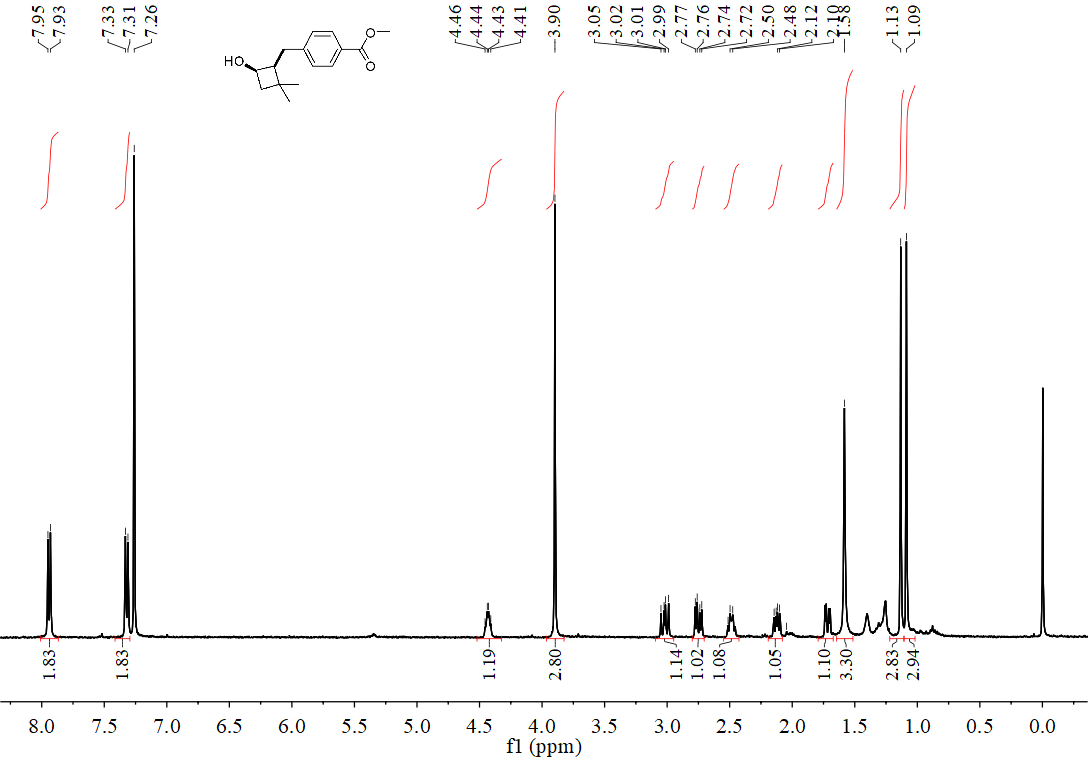
**

**
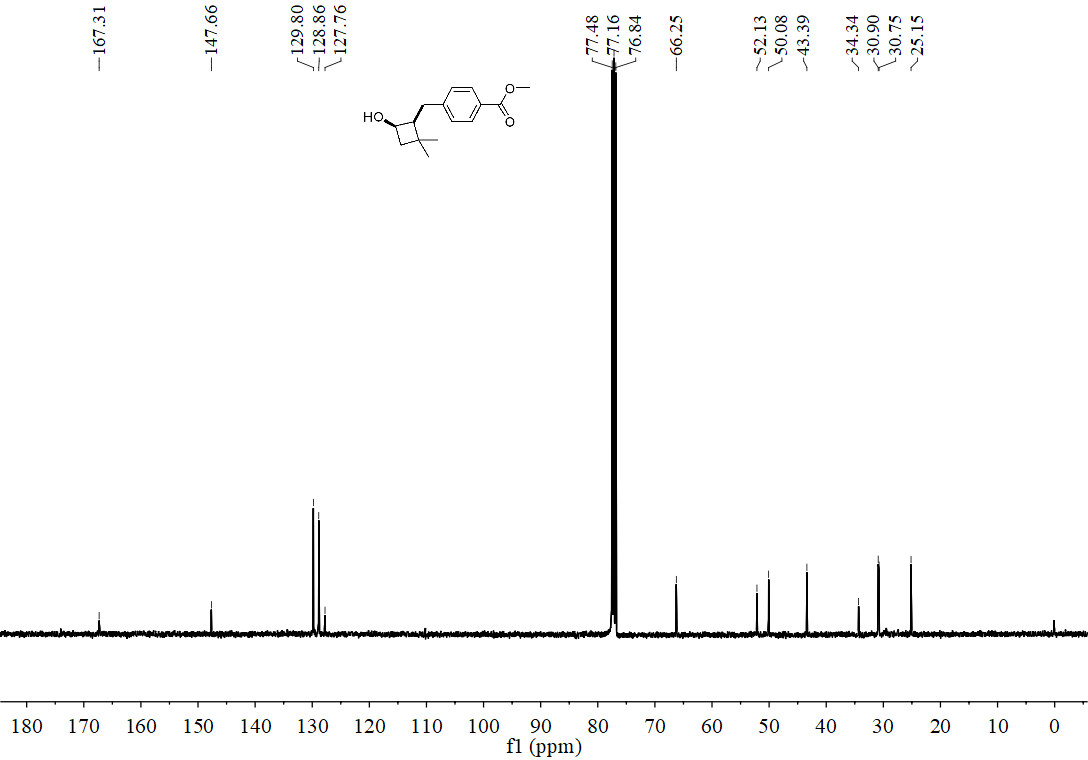
**

**Supplementary Fig. 49.** ^1^H NMR and ^13^C NMR spectrum of **3q_1_**.

**
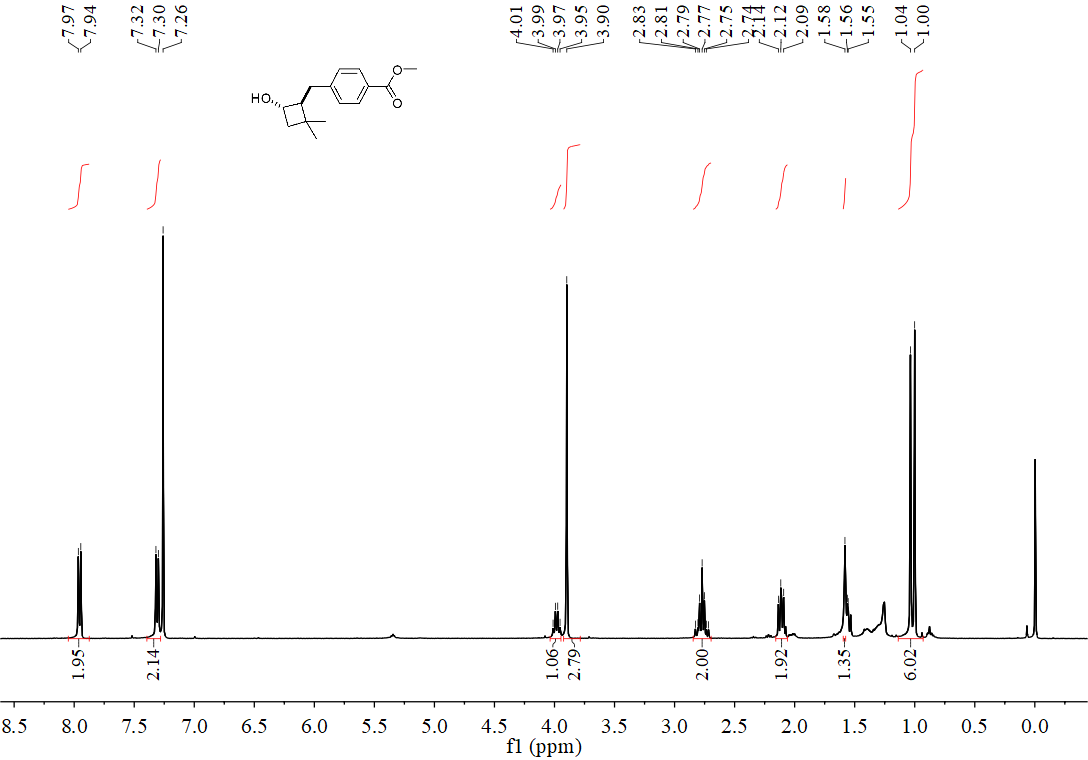
**

**
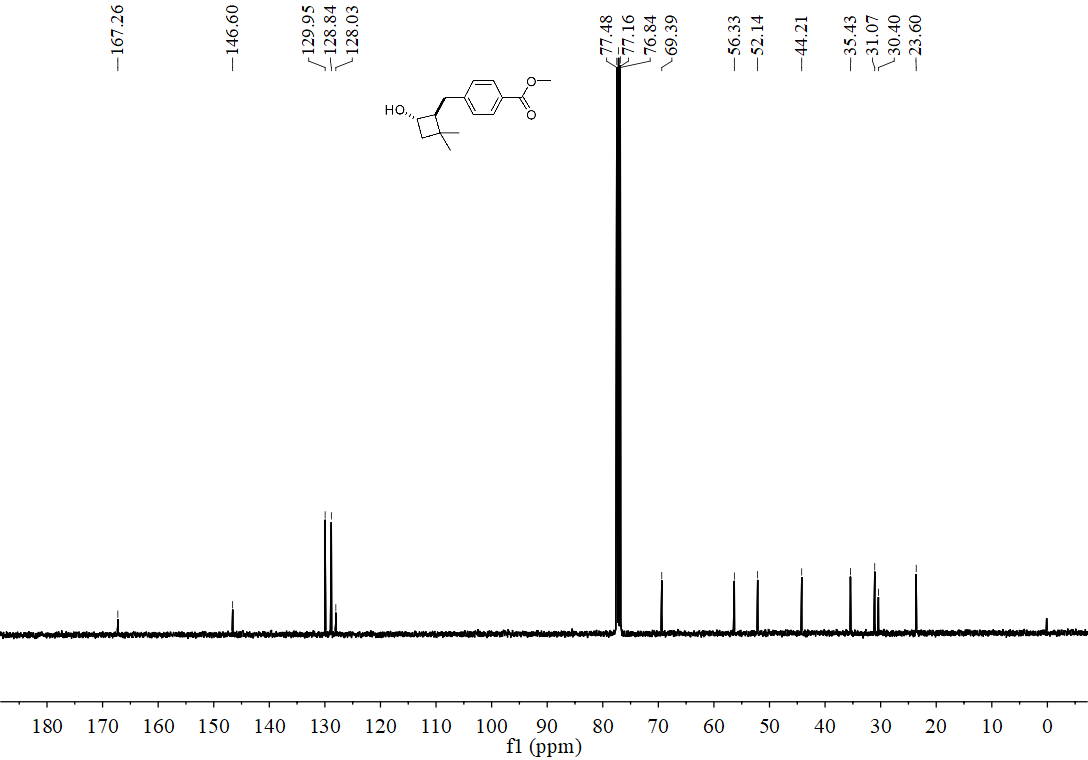
**

**Supplementary Fig. 50.** ^1^H NMR and ^13^C NMR spectrum of **3q_2_**.

**
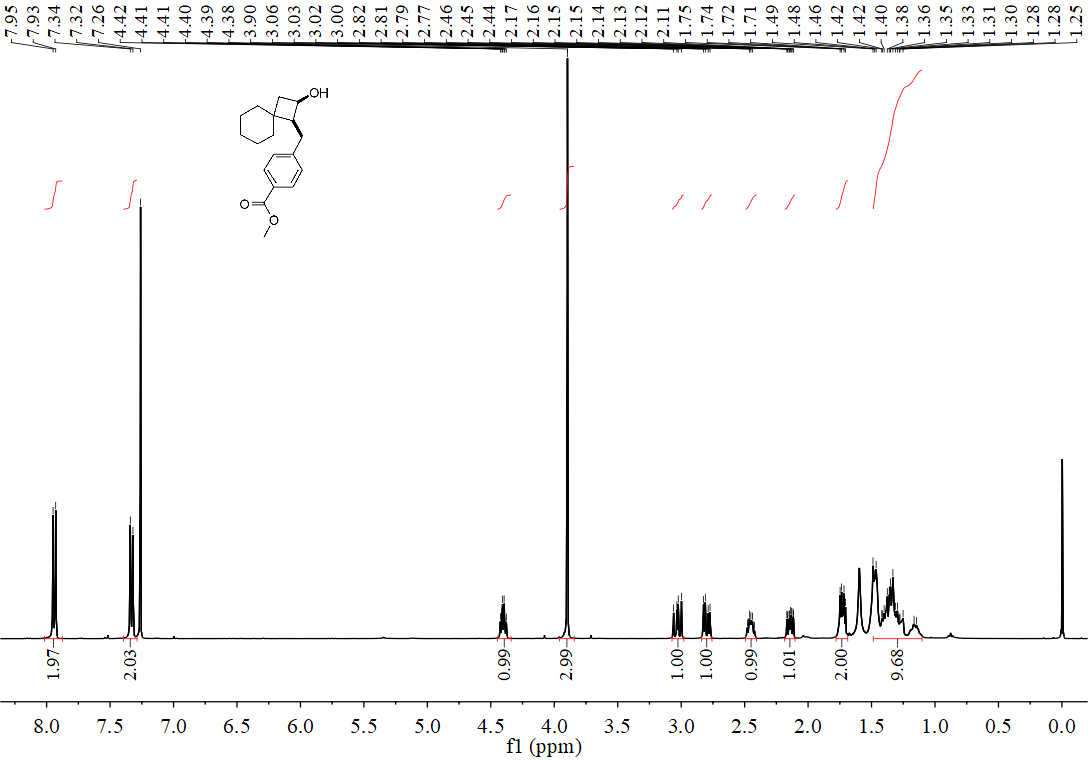
**

**
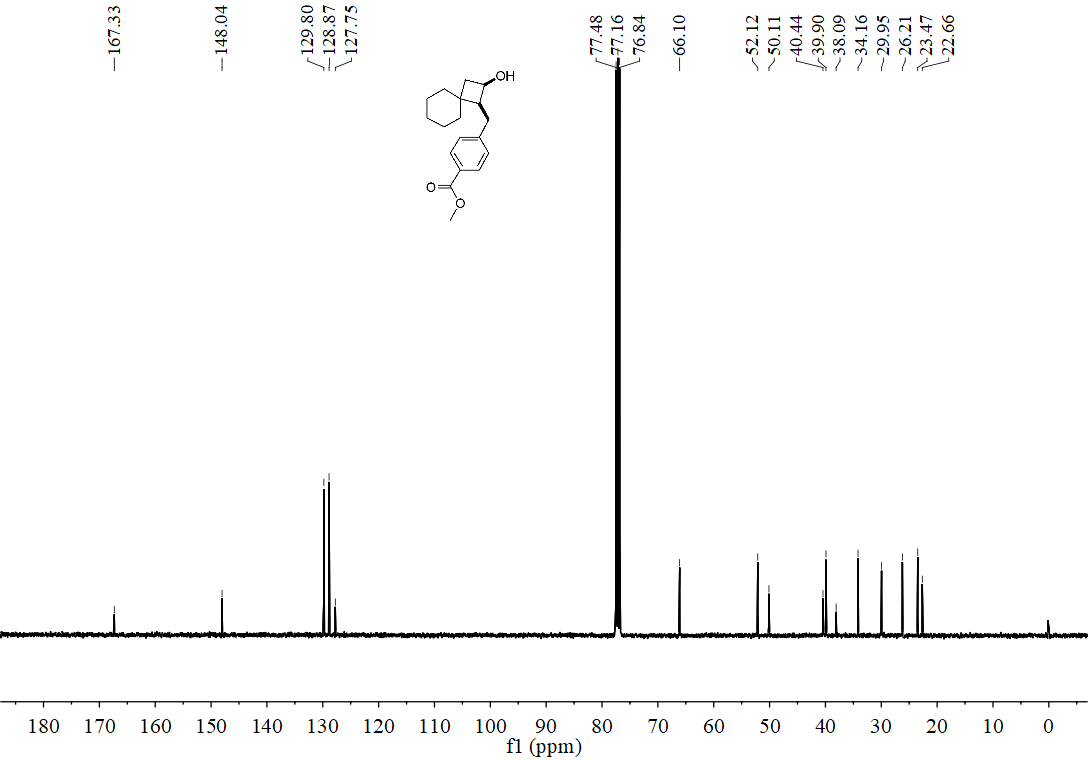
**

**Supplementary Fig. 51.** ^1^H NMR and ^13^C NMR spectrum of **3r_1_**.

**
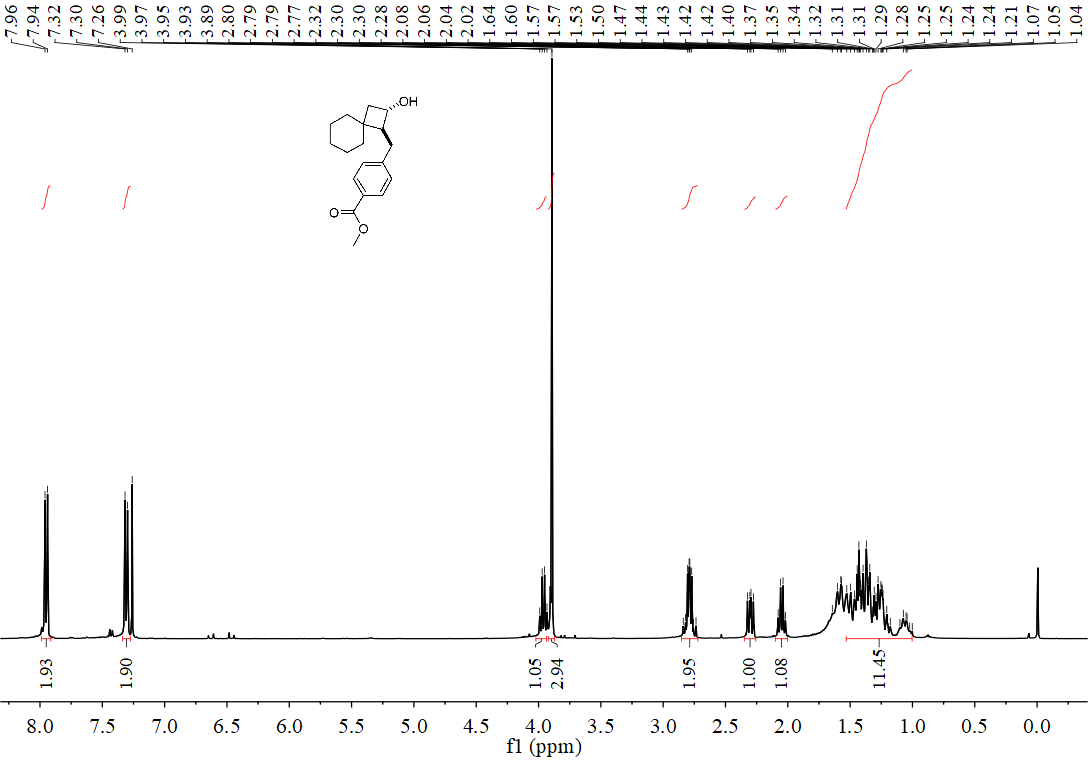
**

**
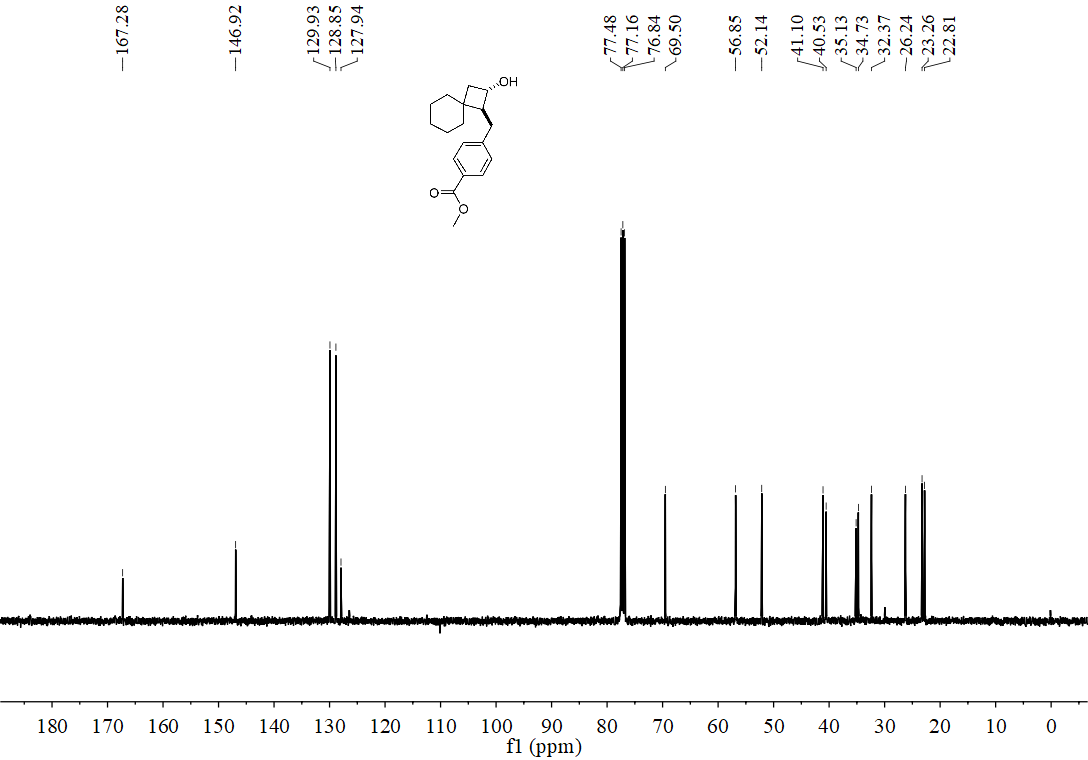
**

**Supplementary Fig. 52.** ^1^H NMR and ^13^C NMR spectrum of **3r_2_**.

**
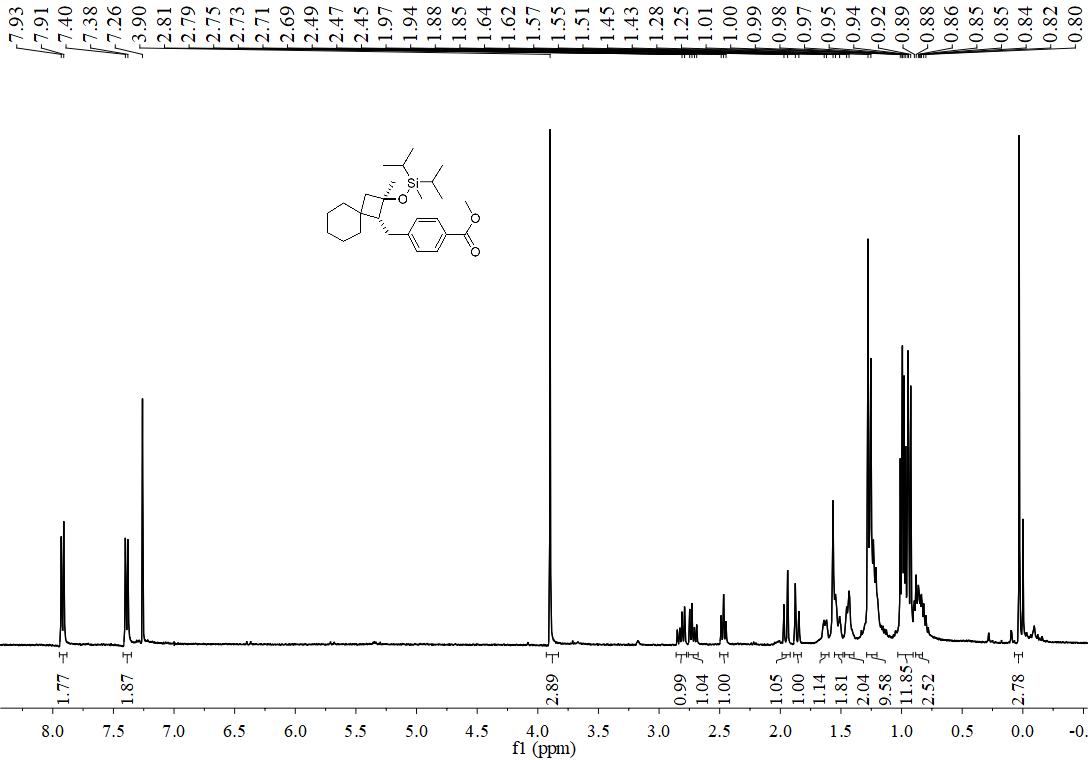
**

**
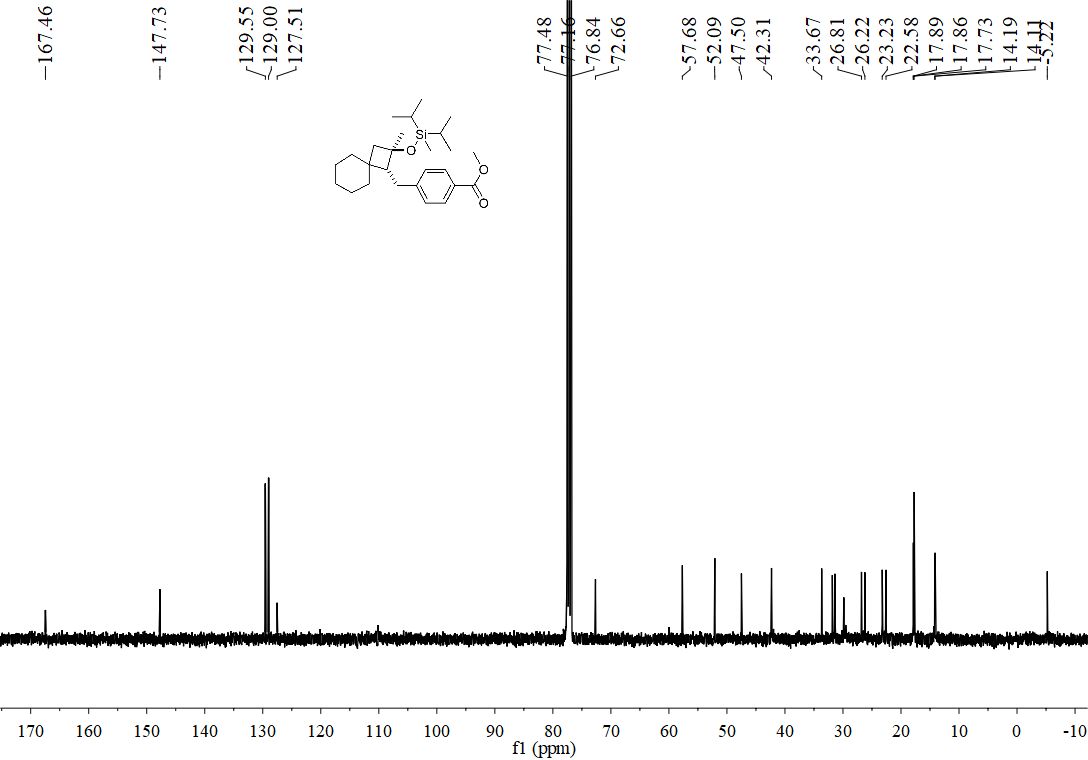
**

**Supplementary Fig. 53.** ^1^H NMR and ^13^C NMR spectrum of **3s**.

**
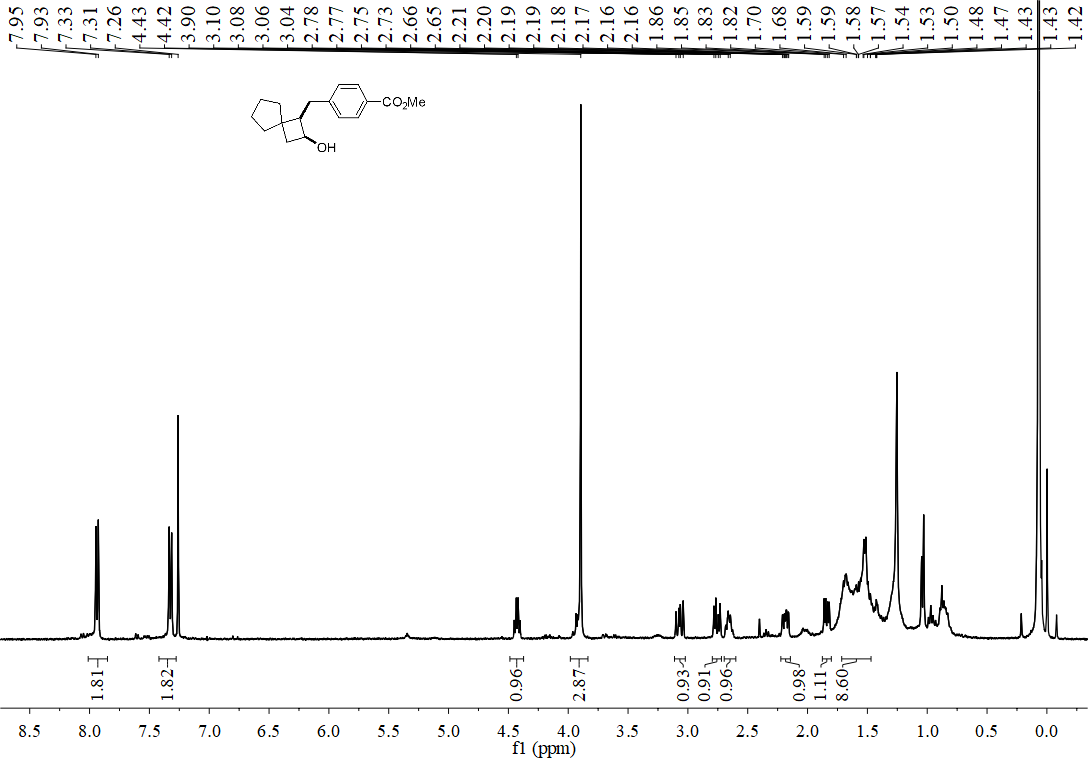
**

**
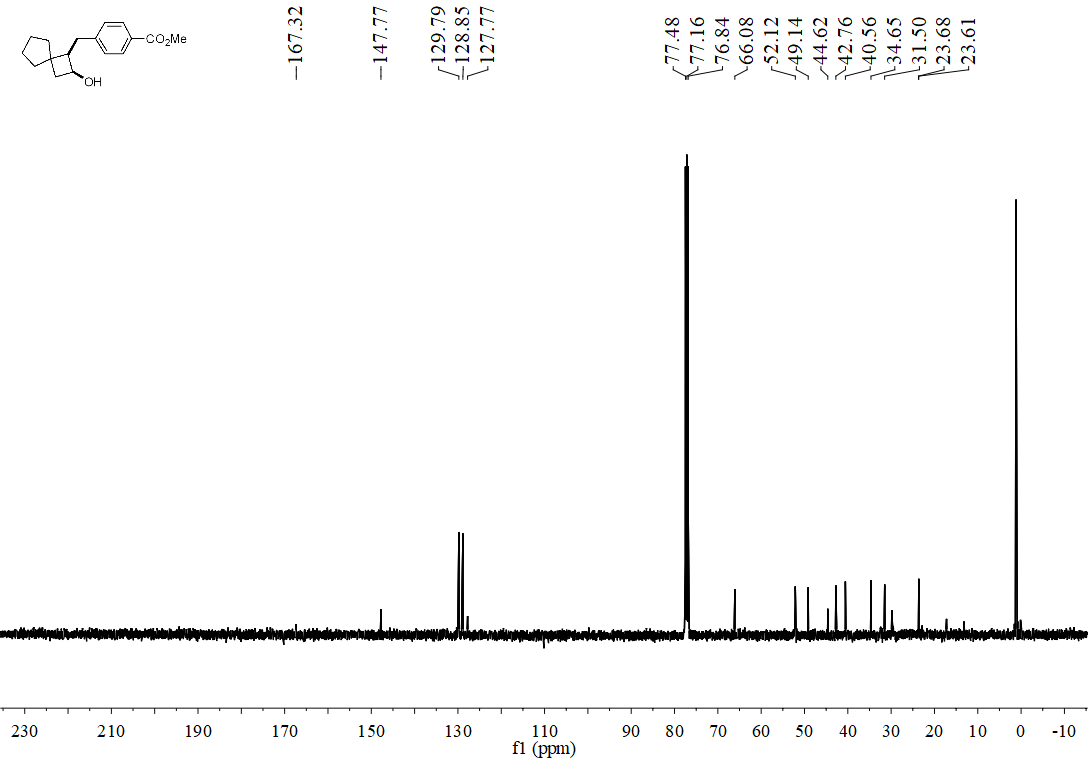
**

**Supplementary Fig. 54.** ^1^H NMR and ^13^C NMR spectrum of **3t_1_**.

**
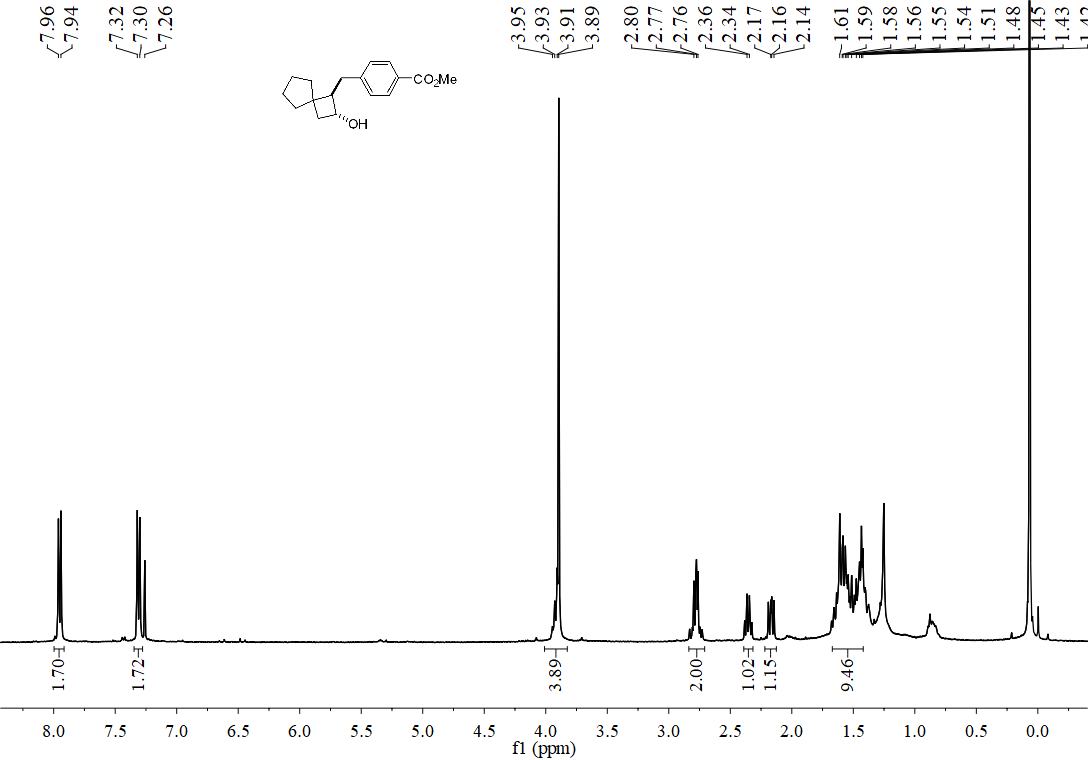
**

**
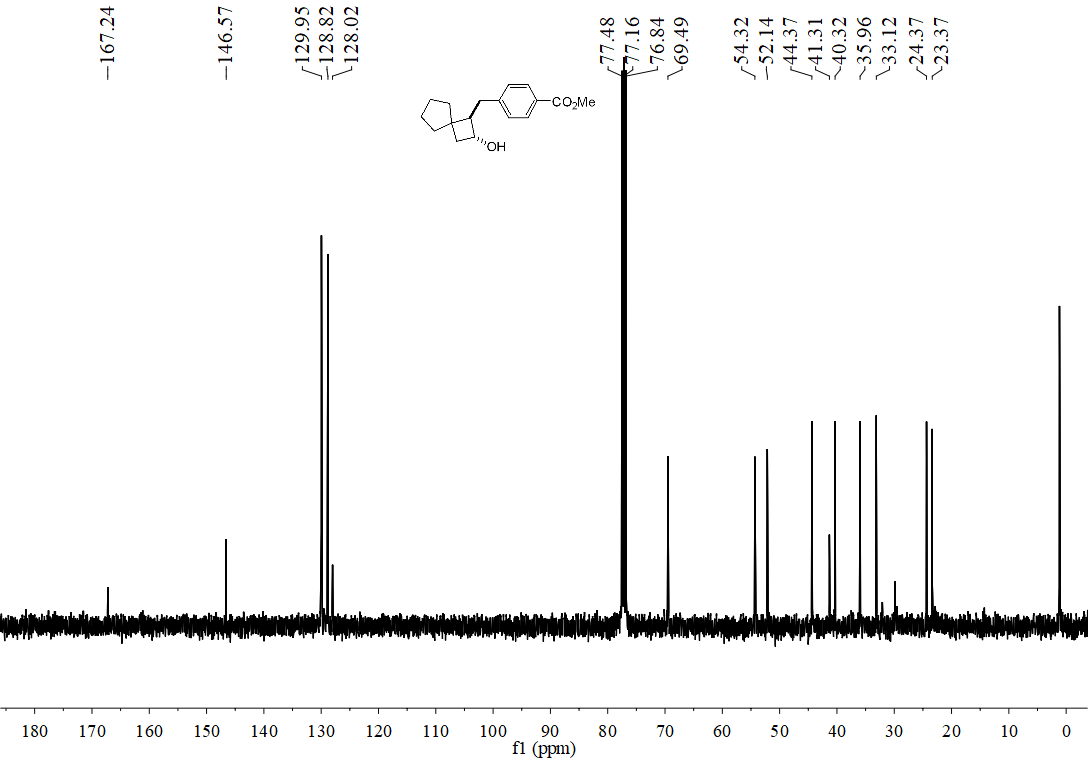
**

**Supplementary Fig. 55.** ^1^H NMR and ^13^C NMR spectrum of **3t_2_**.

**Supplementary Fig. 56.** ^1^H NMR and ^13^C NMR spectrum of **3u_1_**.

**Supplementary Fig. 57.** ^1^H NMR and ^13^C NMR spectrum of **3u_2_**.

**Supplementary Fig. 58.** ^1^H NMR and ^13^C NMR spectrum of **3v_1_**.

**Supplementary Fig. 59.** ^1^H NMR and ^13^C NMR spectrum of **3v_2_**.

**Supplementary Fig. 60.** ^1^H NMR and ^13^C NMR spectrum of **3w_1_**.

**Supplementary Fig. 61.** ^1^H NMR and ^13^C NMR spectrum of **3w_2_**.

**Supplementary Fig. 62.** ^1^H NMR and ^13^C NMR spectrum of **3x_1_**.

**Supplementary Fig. 63.** ^1^H NMR and ^13^C NMR spectrum of **3x_2_**.

**Supplementary Fig. 64.** ^1^H NMR and ^13^C NMR spectrum of **3y_1_**.

**Supplementary Fig. 65.** ^1^H NMR and ^13^C NMR spectrum of **3y_2_**.

**Supplementary Fig. 66.** ^1^H NMR and ^13^C NMR spectrum of **3z_1_**.

**Supplementary Fig. 67.** ^1^H NMR and ^13^C NMR spectrum of **3z_2_**.

**Supplementary Fig. 68.** ^1^H NMR and ^13^C NMR spectrum of **3aa**.

**Supplementary Fig. 69.** ^1^H NMR and ^13^C NMR spectrum of **4**.

**Supplementary Fig. 70.** ^19^F NMR spectrum of **4**.

**Supplementary Fig. 71.** ^1^H NMR and ^13^C NMR spectrum of **5**.

**Supplementary Fig. 72.** NOESY spectrum of **3l_1_**.

**Supplementary Fig. 73.** NOESY spectrum of **3l_2._**

**Supplementary Fig. 74.** H-H COSY and NOESY spectrum of **3p**.

**Supplementary Fig. 75.** NOESY spectrum of **3r_1_**.

**Supplementary Fig. 76.** NOESY spectrum of **3v_1_**.

**Supplementary Fig. 77.** NOESY spectrum of **3v_2._**

**Supplementary Fig. 78.** NOESY spectrum of **3w_1._**

**Supplementary Fig. 79.** NOESY spectrum of **3w_2._**

**Supplementary Fig. 80.** NOESY spectrum of **3y_1._**

**Supplementary Fig. 81.** NOESY spectrum of **3y_2._**

**Supplementary Fig. 82.** NOESY spectrum of **3z_1._**

**Supplementary Fig. 83.** NOESY spectrum of **3z_2._**

**Supplementary Fig. 84.** NOESY spectrum of **3aa_._**

1. Supplementary references
2. Chuentragool, P. *et al.* Aliphatic radical relay Heck reaction at unactivated C(sp^3^)−H sites of alcohols. *Angewandte Chemie International Edition* **58**, 1794-1798 (2019).
3. Kurandina, D. *et al.* Transition-metal- and light-free directed amination of remote unactivated C(sp^3^)–H bonds of alcohols. *Journal of the American Chemical Society* **141**, 8104-8109 (2019).
4. Parasram, M., Iaroshenko, V. O. & Gevorgyan, V. Endo-selective Pd-catalyzed silyl methyl Heck reaction. *Journal of the American Chemical Society* **136**, 17926-17929 (2014).
5. Zhao, H., Fan, X., Yu, J. & Zhu, C. Silver-catalyzed ring-opening strategy for the synthesis of β- and γ-fluorinated ketone. *Journal of the American Chemical Society* **137**, 3490-3493 (2015).
6. Ziadi, A. & Martin, R. Ligand-accelerated Pd-catalyzed ketone γ-arylation via C–C cleavage with aryl chlorides. *Organic Letters* **14**, 1266-1269 (2012).
7. Michelet, B., Deldaele, C., Kajouj, S., Moucheron, C. & Evano, G. A general Copper catalyst for photoredox transformations of organic halides. *Organic Letters* **19**, 3576-3579 (2017).
8. Li, Z. & Lei, J. Visible-Light-Induced Organocatalytic Borylation of Aryl Chlorides. *Journal of the American Chemical Society* **141**, 9124-9128 (2019).
9. Cismesia, M. A. & Yoon, T. P. Characterizing chain processes in visible light photoredox catalysis. *Chemical Science* **6**, 5426-5434 (2015).
10. Wang, P.-Z. *et al*. Asymmetric three-component olefin dicarbofunctionalization enabled by photoredox and copper dual catalysis. *Nature Communications* **12**, 1815 (2021).
